# Phylogenetic, genomic, and biogeographic characterization of a novel and ubiquitous marine invertebrate-associated Rickettsiales parasite, *Candidatus* Aquarickettsia rohweri, gen. nov., sp. nov

**DOI:** 10.1038/s41396-019-0482-0

**Published:** 2019-08-05

**Authors:** J. Grace Klinges, Stephanie M. Rosales, Ryan McMinds, Elizabeth C. Shaver, Andrew A. Shantz, Esther C. Peters, Michael Eitel, Gert Wörheide, Koty H. Sharp, Deron E. Burkepile, Brian R. Silliman, Rebecca L. Vega Thurber

**Affiliations:** 1 Department of Microbiology, Oregon State University, Corvallis, OR 97331 USA; 2 Cooperative Institute for Marine and Atmospheric Studies, University of Miami, Miami, FL 33149 USA; 3 Atlantic Oceanographic and Meteorological Laboratory, National Oceanographic and Atmospheric Administration, Miami, FL 33149 USA; 4 Center of Modeling, Simulation and Interactions, Université Côte d′Azur, Nice, France; 5 Division of Marine Science and Conservation, Nicholas School of the Environment, Duke University, Beaufort, NC 28516 USA; 6 Department of Ecology, Evolution and Marine Biology, University of California, Santa Barbara, Santa Barbara, CA 93106-9610 USA; 7 Department of Biology, Pennsylvania State University, University Park, State College, PA 16802 USA; 8 Department of Environmental Science and Policy, George Mason University, Fairfax, VA 22030-4444 USA; 9 Department of Earth and Environmental Sciences, Paleontology and Geobiology, Ludwig-Maximilians-Universität München, Munich, Germany; 10 GeoBio-Center, Ludwig-Maximilians-Universität München, Munich, Germany; 11 Staatliche Naturwissenschaftliche Sammlungen Bayerns (SNSB) – Bayerische Staatssammlung für Paläontologie und Geologie, Munich, Germany; 12 Department of Biology, Marine Biology & Environmental Science, Roger Williams University, Bristol, RI 02809 USA

**Keywords:** Functional genomics, Microbial ecology, Biogeography, Symbiosis, Phylogenetics

## Abstract

Bacterial symbionts are integral to the health and homeostasis of invertebrate hosts. Notably, members of the Rickettsiales genus *Wolbachia* influence several aspects of the fitness and evolution of their terrestrial hosts, but few analogous partnerships have been found in marine systems. We report here the genome, phylogenetics, and biogeography of a ubiquitous and novel Rickettsiales species that primarily associates with marine organisms. We previously showed that this bacterium was found in scleractinian corals, responds to nutrient exposure, and is associated with reduced host growth and increased mortality. This bacterium, like other Rickettsiales, has a reduced genome indicative of a parasitic lifestyle. Phylogenetic analysis places this Rickettsiales within a new genus we define as “*Candidatus* Aquarickettsia.” Using data from the Earth Microbiome Project and SRA databases, we also demonstrate that members of “*Ca*. Aquarickettsia” are found globally in dozens of invertebrate lineages. The coral-associated “*Candidatus* A. rohweri” is the first finished genome in this new clade. “*Ca*. A. rohweri” lacks genes to synthesize most sugars and amino acids but possesses several genes linked to pathogenicity including Tlc, an antiporter that exchanges host ATP for ADP, and a complete Type IV secretion system. Despite its inability to metabolize nitrogen, “*Ca*. A. rohweri” possesses the NtrY-NtrX two-component system involved in sensing and responding to extracellular nitrogen. Given these data, along with visualization of the parasite in host tissues, we hypothesize that “*Ca*. A. rohweri” reduces coral health by consuming host nutrients and energy, thus weakening and eventually killing host cells. Last, we hypothesize that nutrient enrichment, which is increasingly common on coral reefs, encourages unrestricted growth of “*Ca*. A. rohweri” in its host by providing abundant N-rich metabolites to be scavenged.

## Introduction

Symbioses are common in marine systems, and the stability of these relationships among hosts and symbionts are often essential to each member’s health [1]. Yet symbioses can easily be disrupted by pathogens or abiotic stressors [2–4]. For example, coral reefs are habitats highly dependent on symbioses, and stressors like increased temperatures shift animal microbiomes and drive disease events [5–7], which are major causes of mortality in tropical reefs worldwide [8]. The complex nature of microbiomes and the contextual nature of disease, however, makes it difficult to incontrovertibly identify the causative agents of many epizootics. It is likely that, in addition to identifiably virulent pathogens, other bacterial species make their hosts more vulnerable to harm from subtle environmental changes. Recent research efforts have focused on identifying conditions that lead to dysbiosis between coral host and symbiont, yet few studies have been able to test the contextual roles of the host, microbe, and environment in disease susceptibility [9]. Understanding how these microorganisms are involved in host responses to environmental conditions may provide insight into the elusive causes of marine invertebrate disease.

Our recent study of the endangered Caribbean staghorn coral, *Acropora cervicornis*, identified a Rickettsiales-annotated OTU that increased in relative abundance from 11.4 to 87.9% of the total coral bacterial community after exposure to elevated inorganic nutrients [3]. Furthermore, we found a strong negative correlation between the relative abundance of this Rickettsiales OTU and coral growth [3]. We also showed that members of this taxonomic group are associated with increased tissue loss and mortality in three other species of corals after chronic exposure to elevated nitrate and phosphate [2].

The order Rickettsiales is one of several microbial groups being investigated as a disease agent of corals, but the high prevalence of these bacteria in both visually healthy and diseased corals renders their etiological role unclear [10, 11]. Rickettsiales spp. have been identified in corals suffering from white-band disease type I (WBD I), an epizootic which resulted in the near extirpation of acroporid corals from the Caribbean [7, 8, 10], with up to 95% of the population destroyed since the disease was first documented in the 1980s [8, 10]. Histological samples of both healthy and WBD-afflicted acroporid corals are often dominated by Rickettsiales (or *Rickettsia*)-like organisms (RLOs) [10, 12], but Di Lauro [13] found that the number of RLO aggregates in WBD-infected tissue was positively correlated with worsening coral tissue conditions.

Members of the order Rickettsiales are typically obligate intracellular parasites, often associated with invertebrate vectors and sometimes pathogenic to humans. They have highly reduced genomes, averaging 1.1–1.5 Mb [14–17], with varying amounts of pseudogenization and repetitive regions [16, 18, 19]. Three families are currently defined within Rickettsiales: *Rickettsiaceae, Anaplasmataceae*, and “*Candidatus* Midichloriaceae” [14, 20–22]. *Rickettsiaceae* consists of bacteria that induce spotted fever rickettsiosis in humans [19, 23], while members of *“Ca*. Midichloriaceae” have been shown to infect protists and the mitochondria of metazoans [24–26]. The family *Anaplasmataceae* consists of four genera: *Anaplasma, Ehrlichia, Neorickettsia*, and *Wolbachia* [20].

We report here the genome assembly, phylogenetics, and biogeography of the Rickettsiales OTU identified in our previous studies. For this organism, we propose the novel genus and species “*Candidatus* Aquarickettsia rohweri,” which is named for the association of members of this genus with aquatic organisms and Dr. Forest Rohwer, whose group first described this taxon in corals. This study places “*Ca*. A. rohweri” in the family “*Ca*. Midichloriaceae”, and assesses genome features related to its host dependency and potential virulence mechanisms. The presence of genes for secretion and metabolite acquisition as well as genes involved in sensing extracellular nutrient concentrations suggests that “*Ca*. Aquarickettsia” exploits its host for necessary nutrients, energy, and amino acids. Last, we report that members of “*Ca*. Aquarickettsia” are found not only in corals but also in other aquatic organisms, including sea anemones, sponges, hydrozoans, placozoans, ctenophores, and protists worldwide.

## Materials and methods

### Coral RLO enrichment experiment and coral histology

Following the methods of Shaver et al. [3], we performed nutrient enrichment on fragments of *A. cervicornis* in the field. In brief, we obtained 40 fragments of *A*. *cervicornis* from the Coral Restoration Foundation’s coral nursery in Key Largo, Florida. Fragments were transported by boat to Pickles Reef, Florida, and secured to mounting structures using All Fix Marine Epoxy®. Corals were allowed to acclimate for 1 week before 20 fragments were exposed to 70 g of Osmocote 19-6-12 Smart-Release® Plant fertilizer in mesh bags that were attached ~5 cm below corals. The remaining 20 fragments were maintained under ambient conditions at the site to serve as unenriched controls. Enrichment was maintained for 4 weeks with fertilizer bags replaced after 2 weeks. Shaver et al. [3] demonstrated effective nutrient enrichment using this treatment for up to 8 weeks.

Due to mortality or breakage, 34 coral fragments of the initial 40 were sampled for histological analysis. Fragments were placed in 50-mL centrifuge tubes filled with a formaldehyde-based fixative composed of 1:4 parts Z-Fix Concentrate (Z-Fix Concentrate, Anatech, Ltd.) and Instant Ocean® seawater. Centrifuge tubes were capped, tightly sealed using Parafilm, and shipped to the Histology Laboratory at George Mason University for analysis using methods from Miller et al. [12]. and Price and Peters [27]. In brief, after decalcification with Formical-2000, trimmed subsamples were processed into paraffin blocks. Sections (at 5-μm thickness) were mounted on glass slides, stained with Harris’s hematoxylin and 1% alcoholic eosin and a Giemsa procedure that distinguishes rickettsia from other Gram-negative bacteria [27], and were then visually assessed using an Olympus BX43 compound microscope [12]. Tissue parameters, including cellular integrity, *Symbiodiniaceae* abundance, and tissue components (e.g. epidermal and mesenterial mucocytes) were assessed using a previously established numerical system [12]. Image analysis with Olympus cellSens software was conducted to count numbers of dividing algal symbionts and to obtain gastrodermis thickness in pixel lengths.

### Fluorescence in situ hybridization

The protocol for fluorescence in situ hybridization (FISH) was adapted from a previously described method [28]. Three paraffin-embedded *A. cervicornis* specimens from the high-nutrient treatments were sectioned onto glass slides at a thickness of 10–15 μm. Sections were deparaffinized 2× for 10 min in xylene, 2× for 10 min in 95% ethanol, rinsed once in distilled water, and air dried. FISH with end-labeled oligonucleotide probes was performed in a humidity chamber in hybridization buffer (0.9 M NaCl, 20 mM Tris-HCl [pH 7.4], 0.01% sodium dodecyl sulfate) with 40% formamide for 2–4 h. After hybridization, the slides were incubated at 48 °C in wash buffer (0.7 M NaCl, 20 mM Tris-HCl [pH 7.4], 50 mM EDTA, 0.01% sodium dodecyl sulfate) for 20–40 min, depending on the hybridization incubation period. The wash buffer was rinsed away with distilled water, and slides were air dried and mounted in VectaShield (Vector Labs, Burlingame, CA). Slides were visualized on a Nikon Eclipse E800 epifluorescence microscope. All probes, including the general bacterial probe suite (EUB338-I,EUB338-II, and EUB338-III) that together target most known bacteria, the bacterial probe negative control (EUB338-NEG), and the sequence-specific probes designed in this study (RICK, RICKNEG), were used at a final concentration of 10 ng/μl in hybridization buffer.

### Design and application of species-specific probes

A suite of probes (EUB338-I, EUB338-II, EUB338-III) targeting most bacteria 16S rRNA sequences (EUB338-I: 5′-CTGCCTCCCGTAGGAGT-3′; EUB338-II: 5′-CAGCCACCCGTAGGTGT-3′; EUB338-III: 5′-CTGCCACCCGTAGGTGT-3′) was used for FISH [29, 30]. Probes were designed targeting the “*Candidatus* Aquarickettsia rohweri” 16S rRNA gene sequence obtained from the draft genome for use, as confirmation of their presence in *A. cervicornis* specimens. In order to ensure specificity, the probe was designed to target a hypervariable region of the 16S rRNA, and for efficiency, it targets a region of extremely high accessibility on the 16S rRNA molecule [31]. The specific oligonucleotide probe, RICK (5′-CCTCCAATTCTCCATTGG-3′), was 5′-labeled with either Alexa546 or Alexa488. Inquiry on ProbeMatch (RDP; http://rdp.cme.msu.edu/probematch/search.jsp) using 100% sequence match suggests that the probe only matches 16S sequences previously assigned to *Rickettsia* species or unclassified alphaproteobacteria in the database. A single-mismatch probe, RICKNEG (5′-CCTCCAATACTCCATTGG-3′), was designed as a negative control to confirm probe specificity and matches 0 bacterial sequences in the RDP database. Optimal formamide concentration (40%) for the specific probe was empirically determined by using the highest formamide concentration that simultaneously exhibited presence of mixed EUB338 binding and RICK binding, and absence of RICKNEG binding.

### Metagenome preparation, sequencing, and sequence quality control pipeline

An *Acropora cervicornis* fragment from ref. [3] with a relative abundance of 87.9% for one Rickettsiales OTU was selected for genome assembly. DNA extraction was performed using EZNA Tissue DNA Kit (Omega Bio-Tek), and DNA yield was quantified using the Qubit dsDNA HS Assay and analyzed on a Qubit 3.0 Fluorometer. A barcoded DNA fragment library was prepared using the Nextera XT (Illumina) sample preparation kit (Supplementary Table S1A). This sample was shotgun sequenced on an Illumina Miseq with a v3 reagent kit at OSU’s Center for Genome Research and Biotechnology (CGRB). A total of 13,785,074 metagenomic reads were sequenced with an average sequence length of 301 bp and a mean phred score of 36. Further details about the sample used for genome assembly are accessible as National Center for Biotechnology Information (NCBI) BioSample SAMN10490412.

### Genome assembly and quality assessment

The filtering and trimming of low-quality sequences and removal of adaptor sequences were conducted with FQtrim (Supplementary Table S2). The program PEAR [32] was used with default settings to merge high-quality paired-end sequences. The contaminating reads were filtered out by mapping to the human genome [33], and genomes of *Acropora digitifera* [34] (as there was no genome available for *A. cervicornis*), and the algal symbiont *Symbiodiniaceae* spp[35]., using Bowtie2 [36] (Supplementary Table S3). The remaining 13,688,870 reads (99.46%) were assembled with the de novo meta-assembler IDBA-UD, a de novo assembler [37], and SPAdes [38] were subsequently used to improve assembly. Unsupervised binning of contigs was performed with MaxBin v.2.2.1 [39] using default parameters, and VizBin was implemented to visually bin contigs further. Sequence bins were analyzed by BLAST searches [40] to the NCBI nonredundant database (NR) and to all complete bacterial genomes in RefSeq [41]. Bins with sequences identified as *Trichoplax adhaerens* were retained due to previous observations that genomic scaffolds of this organism contained sequences from Rickettsial endosymbionts that may have been horizontally transferred to the placozoan host [42]. We used SSPACE [43] to merge contigs and create scaffolds, and MaxBin was re-run (with minimum contig length 500) to confirm that all sequences belonged to a single bin. We conducted manual quality control on contigs in this final bin using BLAST, and removed contigs with identity to *Symbiodiniaceae* spp. that had erroneously been retained. CheckM [44] was used to assess assembly quality and contamination (Supplementary Table S4, Fig. S1) using marker genes that are ubiquitous and single copy within a given lineage. We elected to use marker genes from all of Alphaproteobacteria rather than exclusively Rickettsiales due to the large amount of divergence between the three families of Rickettsiales and an overrepresentation of the comparatively well-studied *Rickettsiaceae* in the marker gene database. The program Pseudo-Finder [45] was used to identify potential pseudogenes, which were left in the genome assembly but removed from calculations of coding density (see below).

### Genome annotation and comparative genomics

Prokka [46] was used to annotate the genome, and annotations were uploaded to the KAAS [47] server. KEGG orthology (KO) identifiers were assigned using BLAST+ against the KEGG GENES [48] database with the BBH (bi-directional best hit) method to assign orthologs and enable the reconstruction of KEGG pathways and BRITE hierarchies (Supplementary Table S5). KO numbers linked to the KEGG pathway maps and BRITE functional hierarchies were used to group genes into modules and assess the metabolic pathway completion. OrthoFinder 2.2.3 [49] was used to identify orthologous clusters of genes that were compared between species (listed in Supplementary Table S6) to assess shared and unique gene clusters. CGView [50] was used to generate a circular representation of the genome by providing the program with a Mauve [51] alignment of the draft genome to a reference genome (“*Ca*. Midichloria mitochondrii” strain IricVA) and gene coordinates generated by Prodigal [52] (Supplementary Table S7). EggNOG-mapper [53] was used (DIAMOND mapping, default parameters) to assign eggNOG orthologous groups (OGs), GO terms, and COG functional categories inferred from best matching OGs.

### Module completion analysis with MAPLE

The KEGG pathway completion was examined with module completion ratios of each KEGG functional module using MAPLE-2.3.0 [54]. The completion of each module is directly related to the ability of the organism to perform the function related to the module, and MAPLE corrects for existence of KOs that are shared between independent modules to provide accurate statistics for completion. We provided MAPLE with unannotated coding sequences from Prodigal, allowing us to assess different gene abundances as a measure of module robustness.

### Placozoan-associated bacterial 16S rRNA gene sequencing

A set of primers was selected to amplify the Rickettsial endosymbionts from a diversity of placozoans. The full-length Rickettsial 16S rRNA gene sequence was identified in the draft metagenomes of three placozoan species to select conserved regions to amplify an almost complete bacterial 16S fragment. Draft genomes were from *Trichoplax adhaerens* (“Grell” strain) [55], *Trichoplax* sp. H2 [56], and *Hoilungia hongkongenis* [57]. To amplify and Sanger-sequence the cleaned PCR product, two nested sets of primers were used (Supplementary Table S1b). PCR was conducted using GoTaq (#M3175; Promega) with 25 mM MgCl_2_, 10 mM each dNTP, 5 µM each primer, 1 µ Taq polymerase. The PCR program included 3 -min pre-heating (95 °C) followed by 35 cycles of 30 s heating (95 °C), 30 s annealing (63 °C), and 1 min 30 s extension (72 °C). After the cycles, a final extension step of 3 min (72 °C) completed the PCR. The Rickettsial 16S was amplified from DNA isolations of either newly isolated single individuals (see ref. [58]) or from established clonal cultures (see ref. [57]). For details on isolates see Supplementary Table S8.

### Phylogenetic analysis of coral-associated Rickettsiales

The full-length 16S rRNA region of the genome was identified using BLASTn searches against 16S rRNA regions from other Rickettsiales species from Greengenes (Version 13.8) [59] as a partial 16S rRNA sequence from this OTU already existed in the Greengenes database [3]. The trimmed sequence was aligned to 16S rRNA sequences from 79 members of Rickettsiales and 11 Alphaproteobacteria outgroups (Supplementary Table S9) using cmalign (part of the INFERNAL 1.1.1 package) [60] with a covariance model built using the Rfam seed alignment for bacterial small subunit ribosomal RNA. PhyML [61] was used for phylogenetic analysis, with 1000 bootstrap replicates and parameters selected using jModelTest [62] (Supplementary Table S10). These replicates were summarized using the Geneious 11.1.4 Consensus Tree Builder command [63]. Sequences were manually BLASTed to the NCBI RefSeq database [41], and metazoan hosts were identified from 100% identical sequences in the database with reported hosts from NCBI sequence description information. Pairwise BLAST was performed between the 16S rRNA sequence of “*Ca*. A. rohweri” and closely related sequences from “*Ca*. Midichloriaceae.”

A concatenated marker protein-based phylogeny was generated using a previously curated set of 92 orthologous, single-copy marker genes present in all bacteria [64] (Supplementary Table S11). Hmmsearch 3.1b2 [65] was used to identify these marker genes in 20 genomes within Rickettsiales and four outgroups. Sequences were aligned using MAFFT 7.310 [66] and were concatenated, excluding alignment positions that contained a gap in more than 50% of sequences. Phylogenetic reconstruction was performed using the concatenated alignments in amino acid format, as well as Dayhoff6 [67] recoded format. All analyses were performed under CAT-GTR using PhyloBayes MPI 1.5a [68]. CAT-GTR was shown to be the best-fit evolutionary model for analyzing multigene phylogenomic alignments [69–71], and Dayhoff recoding was applied to rectify the negative impact of compositional heterogeneity [70]. Two independent Markov chains were run and their convergence assessed using the PhyloBayes tools *bpcomp* and *tracecomp* to monitor maximum discrepancy in clade support (maxdiff), the effective sample size (effsize), and relative difference in posterior mean estimates (rel_diff) as summary statistics of the model. The appropriate number of samples to discard as “burnin” was independently determined first by visual inspection of parameter trace plots, and then by optimizing convergence criteria. For amino acid analyses, the maxdiff statistic was 0, while for Dayhoff6 recoded data the statistic was 0.046. The minimum effective sample size was >50 (>100 for Dayhoff6) and the maximum rel_diff statistic was <0.3 (0.1 for Dayhoff6). Node support was evaluated using posterior probabilities.

### Redbiom search for presence of “*Ca.* A. rohweri”

The Earth Microbiome Project (EMP) tool Redbiom [72] was used to search for the presence of “*Ca*. A. rohweri” and other OTUs within the genus “*Ca*. Aquarickettsia” in the Qiita database. The Rickettsiales OTU from the sample selected for “*Ca*. A. rohweri” genome assembly was determined by Shaver et al. [3] to match most closely to Greengenes [59] OTU 150441, so this and the five most closely related Greengenes OTUs (as determined using the Greengenes reference phylogeny and confirmed by BLAST to NR) were considered members of the genus. The command “redbiom search features” was used to search 173,714 16S rRNA libraries from the EMP sequencing context “Pick_closed-reference_OTUs-Greengenes-illumina-16S-v4-100nt”. This context provides the most resolution of the 16S rRNA sequence without losing samples that were not sequenced to 150 bp. Overall, 3168 EMP samples from 83 “sample types” had OTUs assigned by 97% closed-reference picking to “*Ca*. A. rohweri” (Greengenes ID 150441). Many of the categories were, however, either ambiguous (e.g., “fragment” and “sediment”), redundant (e.g., “seawater” and “ocean water”), or miscategorized (e.g., sample type listed as sponge tissue, other metadata fields listed sample as water). For clarity, we manually re-annotated these into 23 categories: by compiling the available metadata files, we were able to differentiate samples into more explanatory biomes (e.g., “coral (fragment)” and “reef sediment”). A map of the distribution of “*Ca*. A. rohweri” in corals was generated by plotting the sample collection coordinates and host genus, available as part of the EMP metadata (EMP Study ID 10895: Global Coral Microbiome Project, and EMP Study ID 10798: Palmyra Atoll Corallimorph and Bleaching Surveys). Analyses through Redbiom were confirmed by analysis using the tool IMNGS [73] to query 303,361 16S rRNA libraries in the Sequence Read Archive (SRA) database. The full-length 16S rRNA sequence was queried against the database at 97% and 99% sequence identity thresholds.

## Results and discussion

### Rickettsia-like organisms are visually abundant in nutrient-enriched corals

**Fig. 1:**
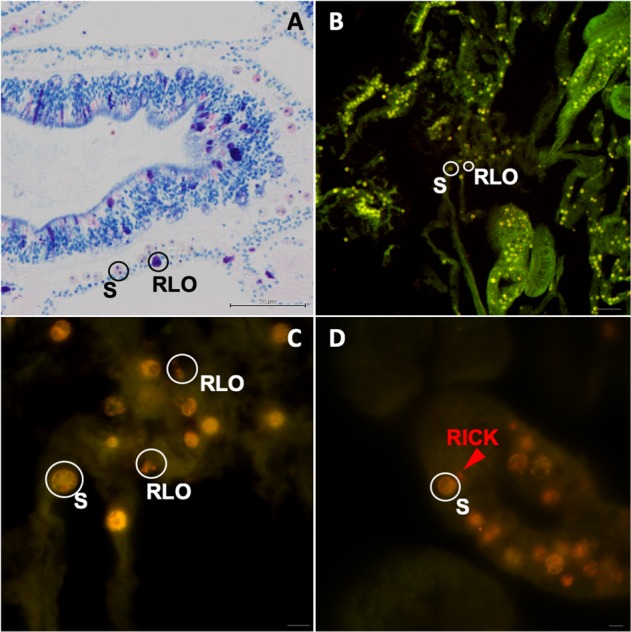
FISH on *Acropora cervicornis* sections with general and specific probes. **a** Suspected RLOs, evident in Giemsa-stained section of actinopharynx body wall, including mucocyte among *Symbiodiniaceae*-infected cells in the gastrodermis. **b**, **c** Ten micrometers thick sections, mixed EUB338-I+EUB338-II+EUB338-III (Alexa546, red) and RICK (Alexa488, green) probes co-localized to clusters of bacterial cells of similar size, morphology, and location as in the Giemsa-stained sections. **d** RICK probe localizes to cells in an aggregate (red arrow), and there is no signal from single-base mismatch probe RICKNEG (green). RLO Rickettsiales-like organism clusters, S *Symbiodiniaceae*. **a** Bar = 50 µm; **b** bar = 50 µm; **c** bar = 10 µm; **d** bar = 10 µm

Suspected RLOs were present in the Giemsa-stained coral samples, both control and nutrient-enriched (see the Supplementary Results Section 1, Fig. S1, Fig. S2). Fluorescent probes targeting most bacteria (mixed EUB338-I+EUB338-II+EUB338-III) and the Rickettsiales-like organism (RICK) co-localized on clusters of cells within the epithelia of *A. cervicornis* (Fig. 1b, c). The clusters of RLOs appeared to be intracellular and near *Symbiodiniaceae* cells, but they were not in or on the *Symbiodiniaceae* cells in the gastrodermis. The RLO-targeted probe (RICK) designed in this study is sequence specific: at 40% formamide, RICK hybridizes to cells in the clusters but no signal from RICKNEG is apparent (Fig. 1d). All images shown are from a single specimen, but the images are representative of signal localization in all examined specimens.

### Genome assembly of “*Ca.* A. rohweri” had low contamination and high completion

**Fig. 2:**
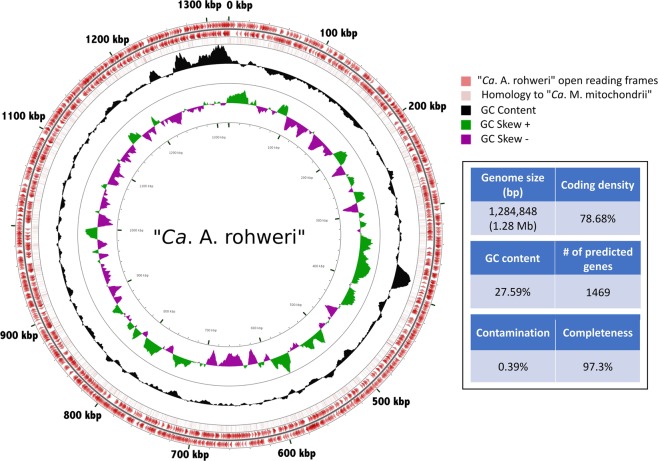
Circular representation of “*Ca*. A. rohweri” genome (red) with red arrows indicating open-reading frames (ORF), light red bars inidicating homology to “Candidatus Midichloria mitochondrii IricVA,” black histogram indicating GC content with purple and green histograms indicating GC+/− skew. GC skew is calculated as (G−C)/(G+C), with positive skew indicating overabundance of G over C and negative skew indicating overabundance of C over G

To evaluate the function of the nutrient-responsive Rickettsiales OTU in *A. cervicornis*, we conducted metagenomic sequencing, de novo genome assembly, and comparative genomics. The genome was assessed for divergence from related genomes by aligning the draft genome to a reference from “*Ca*. Midichloriaceae” [50] (Fig. 2; Fig. S3). The assembled 1.28 Mb genome was estimated at 97.3% completion, with 1469 predicted genes, a coding density of 87.6% and a low GC content of 28.38% (Fig. 2; Supplementary Table S2). Contamination was low as estimated by CheckM at 0.39% (Fig. S4), and completion was estimated at 97.3%, supported by the presence of tRNAs for all 20 proteinogenic amino acids. These genome features are characteristic of most Rickettsiales [18, 20]. At 155 contigs, an N50 of 10,860 bp, and based on completeness and contamination estimates from CheckM, this genome is classified as a “High-Quality Draft“ by MIMAG [74] standards for metagenome-assembled genomes. There were 84 potential pseudogenes identified, most of which were annotated as hypothetical genes. Of the identified pseudogenes, 56 were truncations (CDS of <65% of the average length of BLAST hits to this gene, usually caused by premature stop codon), and 28 were predicted fragmentations (genes split into multiple pseudogenes by presence of an early stop and a second start codon). The coding density without pseudogenes is 78.68%. This genome is accessible on NCBI as accession NZ_RXFM00000000.1.

### Phylogenetics supports the establishment of a new genus within “*Ca.* Midichloriaceae”

**Fig. 3:**
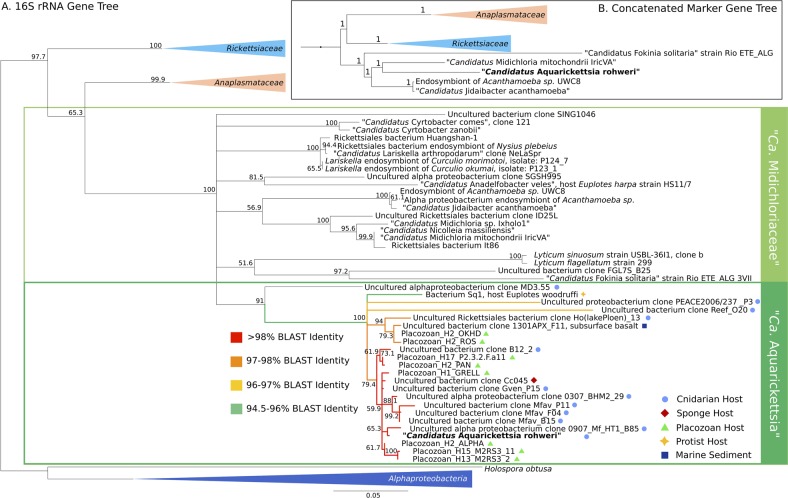
Consensus maximum likelihood and concatenated marker gene tree of Rickettsiales. **a** Phylogeny generated from bootstrapped data sets using 16S rRNA sequences and PhyML with 1000 bootstrapped replicates. Branches within the proposed genus *Aquarickettsia* are colorized by BLAST identity to the 16S rRNA sequences for “*Ca*. **a** rohweri.” Bootstrap confidence values listed at nodes, representing the certainty of that node in the phylogenetic tree, where 100 is maximum certainty. **b** Bayesian phylogeny generated from concatenated amino acid alignment of 92 orthologous, single-copy marker genes for bacteria. Node support was evaluated using posterior probabilities. Branch lengths in both phylogenies correspond to evolutionary distance. Outgroups and Rickettsiales families other than “*Ca*. Midichloriaceae” are collapsed; uncollapsed phylogenetic trees are presented as Supplementary Figs. S5 and S6

Using the recovered full-length 16S rRNA gene sequence from our coral-associated Rickettsiales, our phylogeny (Fig. 3a; Fig. S5) placed our draft genome, “*Ca*. A. rohweri,” within “*Ca*. Midichloriaceae.” Our 16S rRNA phylogeny is consistent with a recent phylogenomic tree [75], which placed “*Ca*. Midichloriaceae” as a sister clade to *Anaplasmataceae* rather than *Rickettsiaceae*. Our phylogeny disagrees with older phylogenomic trees [20, 42] on this placement but this node is supported by relatively low bootstrap values (65.3). Phylogenomic analysis (Fig. 3b; Fig. S6) further supports the placement of “*Ca*. Aquarickettsia” in “*Ca*. Midichloriaceae” (uncollapsed branches), but places “*Ca*. Midichloriaceae” as the sister group to both *Anaplasmataceae* and *Rickettsiaceae*. Strikingly, “*Ca*. A. rohweri” was part of a distinct clade along with other uncultured and uncharacterized bacteria associated with marine invertebrates. This distinct clade, present in other phylogenies [76, 77] but never formally defined, contains symbionts of the soft coral *Gorgonia ventalina*, the stony corals *Orbicella annularis, Orbicella faveolata*, and *Acropora cervicornis*, and the sponge *Cymbastela concentrica*. While most members of this group were identified in marine hosts, several species were identified in freshwater organisms, including the ciliate *Euplotes woodruffi* and freshwater cnidarian *Hydra oligactis*. “*Ca*. A. rohweri” shared species-level homology with multiple 16S rRNA sequences from endosymbionts of *Trichoplax adhaerens*, as well as with sequences from both sponge and coral hosts (Fig. 3, colorized by percentage BLAST identity). The bootstrap value for this distinct clade of marine endosymbionts was 91.

The high confidence bootstrap values joining this clade together support the assignment of a new genus to these intracellular symbionts of marine invertebrates. We propose the genus name “*Candidatus* Aquarickettsia,” with the draft genome from this study named as “*Candidatus* A. rohweri” in reference to its association with aquatic organisms and Dr. Forest Rohwer, who first described this taxon in corals [10].

### Comparative genomics confirms genome content similar to other members of Rickettsiales

**Fig. 4:**
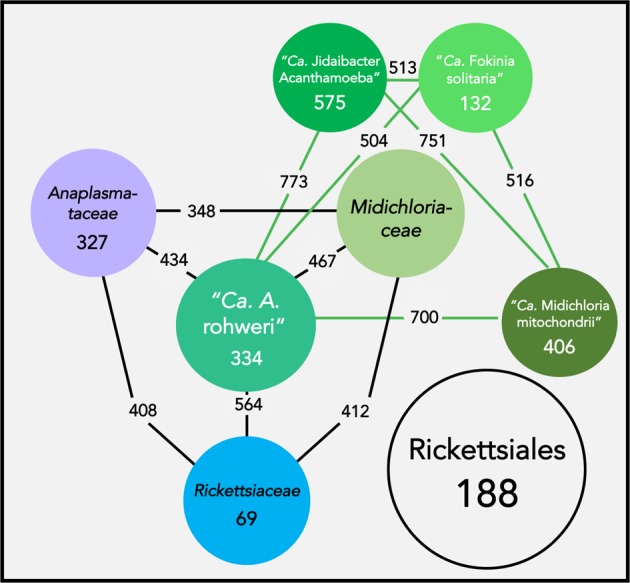
Orthologous gene sets shared by species of Rickettsiales. Network graph showing orthologous gene sets shared by species of Rickettsiales at the order level (Rickettsiales), family level (*Rickettsiaceae, Anaplasmataceae, Midichloriaceae*), and genus level (“*Ca*. A. rohweri”, “*Ca*. M. mitochondrii”, “*Ca*. J. acanthamoeba”, and “*Ca*. F. solitaria”). Numbers in black indicate shared gene sets between two compared organisms or families, or gene sets shared by an entire family sampled. Numbers in white indicate individual genes that were unique to a given organism. Orthologous gene families were identified using OrthoFinder

Based on analysis of orthologous gene sets using OrthoFinder 2.2.3 (Fig. 4), “*Candidatus* A. rohweri” shares 188 gene clusters with other species of Rickettsiales, 327 gene clusters with only members of “*Ca*. Midichloriaceae” (“*Ca*. M. mitochondrii”, “*Ca*. Fokinia solitaria”, and “*Ca*. Jidaibacter acanthamoeba”), and encodes two unique gene clusters (genes that share a similar function, but are present only in the genome of “*Ca*. A. rohweri”) as well as 324 unique genes (not assigned to any orthogroup). While 262 of the unique genes were hypotheticals, 14 of the 62 annotated unique genes (Supplementary Table S9) are involved in oxidative phosphorylation. Other unique genes include transporters, FeS cluster assembly proteins (SufB, SufC, SufD, SufE, not commonly found in Rickettsiales), and flagellar proteins FlgF and FlgL. Of the two gene clusters unique to “Ca. A. rohweri,” one was comprised 87 genes with homology to transposase IS66. Transposable elements are common in Rickettsiales, and IS66 family transposases are annotated in genomes of *Wolbachia* on NCBI (NC_010981 and NC_010981). The other unique cluster consists of hypothetical genes and ankyrin repeats. Ankyrin repeats, along with tetratricopeptide repeats (also present in the genome of “Ca. A. rohweri”), are eukaryotic-like repeat domains which were observed in the genome of “*Ca*. J. acanthamoeba” and may be involved in symbiont–host interactions [75].

### “*Ca.* A. rohweri” is auxotrophic for many essential compounds and relies on the host for metabolic byproducts

Protein prediction and metabolic network analysis revealed 627 KEGG orthology (KO) numbers matching to coding sequences. The KEGG pathways with the greatest numbers of KOs found were: ribosome (47 KOs), oxidative phosphorylation (38), carbon metabolism (34), two-component system (29), biosynthesis of amino acids (24), aminoacyl-tRNA biosynthesis (24), purine metabolism (22), and pyrimidine metabolism (22). Clusters of Orthologous Groups of proteins (COG) [78] functional category analysis mapped 962 genes and indicated that, in comparison with *Pelagibacter ubique* HTCC1062 and *Rhodobacter sphaeroides*, more genes mapped to the categories “Translation” (13% vs. 9% and 4%, respectively), “Replication and Repair” (9% vs. 4% and 4%), “Intracellular Trafficking and Secretion” (4% vs. 2% and 1%), and “Energy Production and Conversion” (10% vs. 8% and 7%), while fewer genes mapped to the categories pertaining to amino acid, nucleotide, and inorganic ion metabolism and transport (Fig. S7).

Module completion ratio analysis revealed incomplete pathways related to carbon and nitrogen metabolism (Fig. S8) and amino acid biosynthesis (Fig. S9), but the presence of a complete *rvh* Type IV secretion system (Fig. S10). Rickettsiales rely heavily on their hosts for nutrients and metabolic byproducts, and “*Ca*. A. rohweri” exhibits many of the same incomplete metabolic pathways as *Rickettsia* spp. and “*Ca*. M. mitochondrii” (Fig. S11).

### “*Ca.* A. rohweri” is deficient in most pathways involved with amino acid biosynthesis

**Fig. 5:**
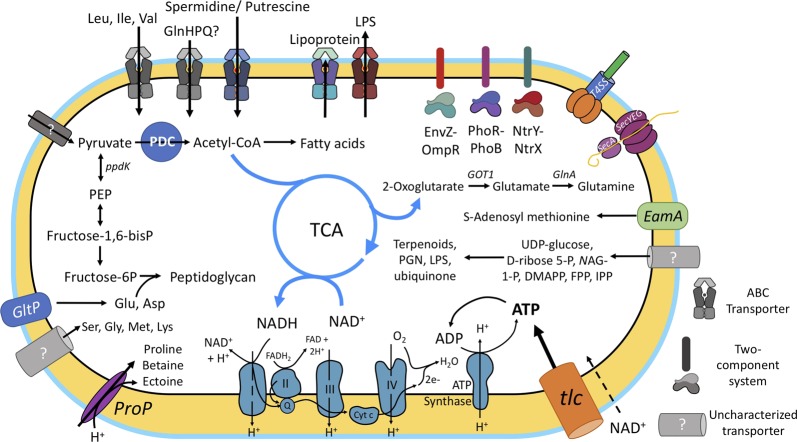
Metabolic reconstruction of “*Ca*. A. rohweri” illustrating known and uncharacterized transporters and known metabolic pathways. “*Candidatus* A. rohweri” must import many host precursors to synthesize compounds such as terpenoids, peptidoglycan, and fatty acids. While some transporters and symporters have been characterized, others are predicted (gray coloration) based on the inability of the organism to synthesize precursors and evidence of uptake by other species of Rickettsiales. DMAPP dimethylallyl pyrophosphate, EamA rickettsial S-adenosylmethionine (SAM) transporter, EnvZ-OmpR osmoregulatory two-component system, FPP *trans,trans*-farnesyl diphosphate, GlnA glutamine synthetase, GlnHPQ glutamine permease ABC transporter, GltP Glu symporter, GOT1 glutamic-oxaloacetic transaminase 1, IPP isopentenyl diphosphate, LPS lipopolysaccharide, *N*AG-1-P *N*-acetylglucosamine-1-P, NtrY-NtrX nitrogen sensing two-component system, Oxidative phosphorylation pathway: complex I NADH:ubiquinone oxidoredutase, complex II succinate:ubiquinone oxidoreductase, complex III ubiquinol:cytochrome c oxidoreductase, cyt c cytochrome c, and complex IV, cytochrome c oxidase. PDC pyruvate dehydrogenase complex, PEP phosphoenolpyruvate, PGN peptidoglycan, PhoR-PhoB phosphate and ferric iron sensing two-component system, ppdk pyruvate phosphate dikinase, ProP proline/glycine betaine transporter, SecYEG/SecA bacterial secretion system (Sec), T4SS Type IV secretion system, TCA tricarboxylic acid cycle, tlc ADP/ATP translocase gene, UDP-glucose uridine diphosphate glucose

The exploration of functional gene categories of “*Ca*. A. rohweri” provided us with an outline of this organism’s potential metabolic function. Due to the absence of most amino acid biosynthesis genes (Fig. 5), this bacterium requires supplementation of every amino acid from the host or algal symbiont except for serine, glycine, glutamate, aspartate, lysine, and threonine. Acroporid corals lack the ability to synthesize the essential amino acids or synthesize cysteine from homocysteine or serine and are thus dependent on *Symbiodiniaceae* for these compounds [34]. *Symbiodiniaceae* are, however, able to synthesize all essential amino acids with the exception of histidine and lysine [79]. The close proximity of “Ca. A. rohweri” to *Symbiodiniaceae* spp. in some FISH imagery in the gastrodermis suggests that amino acids may be acquired directly from the algal symbiont.

Experimental studies in other Rickettsiales (summarized in ref. [80]) have indicated the ability to import amino acids that cannot be synthesized. In addition, while genes (*glyA* and *ltaE*) are present to interconvert Gly to Ser and Thr (Fig. S9), the organism requires at least one of these three amino acids, and studies of *Rickettsia* show the import of Ser and Gly to be required for growth [80]. Lys, Asp, Gln, and Glu can be synthesized from oxaloacetate, but can also be imported. Proton glutamate symport protein (GltP) imports Asp and Glu, ABC transporter GlnHPQ is able to transport Gln in other Rickettsiales but is missing a gene in our existing annotations, and an uncharacterized ABC transporter is predicted to transport Lys. Another ABC transporter, ArtMPQ, can transport Arg and the proline/betaine symporter can import Pro (ProP, Fig. 5). ProP exists in the genome of “*Ca*. A. rohweri” in multiple sequence variants that may transport other amino acids. Other species of Rickettsiales have been shown to import Met, Ser, and Gly, but these transporters are uncharacterized.

### Mechanisms of energy synthesis, transport, and storage in “*Ca*. A. rohweri”

The cell membrane of Rickettsiales spp. has been shown to be permeable to host NAD+ [80]. Although “*Ca*. A. rohweri” potentially can produce ATP through oxidative phosphorylation coupled to electron transport and driven by Glu oxidation, it also possesses an ATP/ADP symporter (nucleotide translocase; Tlc1). Tlc1 allows for the import of ATP in exchange for ADP, effectively sapping the host of energy (Fig. 5). Rickettsiales spp. utilize this system to compensate for their reduced metabolism, and are thus considered energy parasites [16, 75]. Nucleotide transport proteins are essential to the lifestyle of many obligate parasites including *Chlamydia* and *Rickettsia* where they function as the main energy supply, but are also found in free-living bacteria such as Cyanobacteria [81, 82]. *Wolbachia* spp., though host-associated, lack *tlc*. Species of *Rickettsia* encode up to five copies of Tlc (Tlc1 to Tlc5); only Tlc1 functions as an ATP transporter, while Tlc4 and Tlc5 import host ribonucleotides [80]. “*Ca*. A. rohweri” encodes only one copy, with 51% amino acid identity to the Tlc1 gene of “*Ca*. J. acanthamoeba”.

While “*Ca*. A. rohweri” possess a functional pyruvate dehydrogenase complex (PDC) and tricarboxylic acid (TCA) cycle, there is no evidence of functional glycolysis/gluconeogenesis (Fig. 5). *Rickettsia* can import pyruvate from the host through an uncharacterized transporter, and import UDP-glucose as the main sugar source for lipopolysaccharide and peptidoglycan biosynthesis (Fig. 5). Unlike *Rickettsia* spp., our organism lacks the pathway to synthesize PHB, a storage molecule that retains energy to be used when host energy sources are depleted or unavailable. “*Ca*. A. rohweri” possesses three copies of EamA, which transports the cosubstrate *S*-adenosylmethionine (SAM) [80]. The presence of other rickettsial signatures (ProP, GltP, SpoT, and MdlB) [80] confirms the necessity of these genes for the obligate intracellular lifestyle of these bacteria even in diverged hosts.

### “*Candidatus* A. rohweri” can detect, but is incapable of metabolizing nitrogen species

“*Candidatus* A. rohweri” lacks any complete pathways for nitrogen metabolism (Fig. S8), and has only two genes present within this module (*gudB/rocG* glutamate dehydrogenase, *glnA* glutamine synthetase), which are utilized for other purposes. Despite an inability to metabolize nitrogen, “*Ca*. A. rohweri” does possess the NtrY-NtrX two-component system for regulating nitrate metabolism (Fig. 5). NtrY is a transmembrane protein that allows the cell to sense extracellular nitrogen levels, while NtrX is normally involved in regulating *nif-*genes involved with nitrogen fixation [83, 84], which the genome of “*Ca*. A. rohweri” does not encode. The function of this system is poorly understood, but these genes are upregulated by uptake of Pro and Gln from the host cell in other Rickettsiales systems and are likely involved with cell proliferation within the host [83]. Both Rickettsiales and *Pelagibacter* encode the NtrY-NtrX two-component system as well as the EnvZ-OmpR system (Fig. 5) for the control of osmotic stress [17, 85]. The presence of two-component regulatory systems enables these organisms to sense environmental changes and rapidly respond to stimuli by activating or repressing certain genes [84, 85]. These systems have been implicated not only in environmental response but also in the ability of pathogenic bacteria to infect and survive in their hosts, making them advantageous for both intracellular and free-living bacteria [17]. Unlike *Rickettsia* spp. and “*Ca*. M. mitochondrii,” “*Ca*. A. rohweri” possesses the PhoR-PhoB two-component system for detecting inorganic phosphate limitation, which may also play a role in responding to nutrient enrichment and depletion.

### Rickettsiales vir homolog T4SS may be involved in host attachment and infection

Due to their role in human disease, the mechanisms of pathogenicity in *Rickettsiaceae* are well described, but homologs in “*Candidatus* Midichloriaceae” are not. All *Rickettsiaceae* genomes sequenced thus far encode a reduced Type IV secretion system (T4SS) known as Rickettsiales *vir* homolog (*rvh*), which compared with canonical T4SSs lacks a homolog of *virB5*, the gene encoding the minor pilus subunit [15, 86–89]. T4SSs are annotated in the genomes of “*Ca*. M. mitochondrii,” which has one *rvh* T4SS [90], and “*Ca*. J. acanthamoeba,” which has three T4SS clusters, only one of which is related to *rvh* [75]. T4SSs are cell envelope-spanning complexes through which bacterial cells secrete or take up macromolecules [87, 91, 92]. While “*Ca*. A. rohweri” has annotations for all necessary components of the *rvh* T4SS (Fig. S10), homology to *rvh* genes from other Rickettsiales varied. Trbl VirB10 was the most diverged gene, with a maximum of 52.7% amino acid identity (to “*Ca*. M. mitochondrii”), while VirB11 was the most conserved at 76.8% amino acid identity (to “*Ca*. J. acanthamoeba”) (Supplementary Table S12). Nonetheless, “*Ca*. A. rohweri” appears to retain infective capabilities as *Aquarickettsia* spp. were visualized in coral mucocytes (Fig. 1).

In other species of bacteria such as *Agrobacterium tumefaciens* [91, 92], T4SSs are involved in host cell attachment, DNA transfer, and secretion of virulence factors directly into host cells. A similar role has been proposed for *rvh*, and the function of this system has been demonstrated in *Ehrlichia* and *Anaplasma* to secrete effector proteins [93, 94]. Current data suggest that *rvh* T4SS played a pivotal role in the transition from an extracellular lifestyle to an obligate intracellular lifestyle [17, 87], as it is absent from free-living members of Alphaproteobacteria including *Pelagibacter*.

### Unconventional mechanisms of pathogenicity in “*Ca*. A. rohweri”

The coral-associated “*Ca*. A. rohweri” possesses many genes involved in host signaling that may play a role in recognition and phagocytosis by the host. “*Ca*. A. rohweri” possesses a nearly complete set of genes for flagellar assembly despite its endosymbiotic nature. While “*Ca*. J. Acanthamoeba” and “*Ca*. Fokinia solitaria,” endosymbiotic members of “*Ca*. Midichloriaceae,” possess a full set of genes for flagellar assembly [75, 95], TEM imagery of “*Ca*. Fokinia solitaria” demonstrated the lack of a flagellum despite the presence of necessary genes [95]. Flagellin, encoded by the gene fliC, is a known pathogen-associated molecular pattern, recognized by Toll-like receptor 5, a component of innate immune systems of both plants and animals [9]. Upon detection by the host, flagellin causes the release of nuclear factor NF-κB, a key element of infection response that controls DNA transcription, cytokine production, and cell survival [96]. “*Ca*. A. rohweri” possesses genes to produce and export peptidoglycan and lipopolysaccharides (Fig. 5), also known pathogen-associated molecular patterns [97]. These microbial signals may play an essential role as they trigger host phagocytosis of “*Ca*. A. rohweri,” after which the parasite is able to escape further attack once inside the mucocyte. Transcriptome analysis has indicated that corals affected by WBD increase expression genes involved in phagocytosis of foreign bacteria [5, 13]. The infection model proposed for “*Ca*. J. Acanthamoeba” and supported by TEM suggests that the parasite is taken up by phagocytosis, releases effector proteins into the host cytoplasm via the T4SS to manipulate host gene expression and signal transduction, and recruits to the host endoplasmic reticulum to escape phagolysosomal degradation [75].

The presence of the tlc system in “*Ca*. Aquarickettsia” indicates that these organisms siphon energy from their hosts which, when combined with their dependency on the host for scavenged amino acids and sugars, could rapidly deplete the host of necessary cell-building resources. “*Ca*. A. rohweri” infects coral mucocytes (as shown in Fig. 1), and we suspect that these parasites eventually starve, weaken, and kill them. The coral host must replace its mucocytes in order to maintain defenses against sedimentation and other invading microbes, and the energy expended by the host to replace these cells can lead to chronic stress on the coral. Nutrient enrichment has been shown to increase growth of *Symbiodiniaceae* spp., which may provide “*Ca*. A. rohweri” with more available resources as amino acids, sugars, and lipids are transferred from the algal symbiont to the host. This additional nutrition provided to the host by abundant *Symbiodiniaceae* spp. allows the production of more mucocytes in which “*Ca*. A. rohweri” can proliferate.

### Biogeography analysis confirms the association of “*Ca.* Aquarickettsia” with non-bilaterian metazoans worldwide

**Fig. 6:**
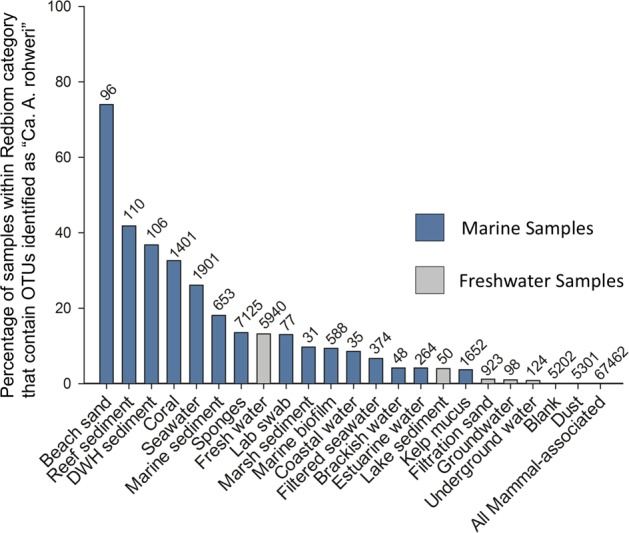
Prevalence of “*Ca*. Aquarickettsia rohweri” 16S sequence within Earth Microbiome Project samples. Bars represent the percentage of samples within each category that had sequences assigned to OTU 150441 (EMP OTU assignment performed with 97% similarity, closed-reference OTU picking with Greengenes). Numbers above bars indicate the total number of samples in that category catalogued in the EMP Qiita database. Blue bars represent samples from marine or brackish environments, and gray bars represent samples from other environments

To survey the global prevalence of this genus and identify new hosts, we used Redbiom to query the publicly available Earth Microbiome Project (EMP) database; in all, our Redbiom analysis represents an evaluation of a total of 173,714 16S V4 Greengenes Illumina 100 nt samples (Fig. 6). “*Ca*. A. rohweri” was prevalent in marine sediments (rocks and sand), seawater, and freshwater, and marine organisms such as corals, sponges, the ctenophore *Mnemiopsis*, and kelp (Fig. 6). The presence of “*Ca*. A. rohweri” in marine sand and sediments suggests either that these habitats provide a potential vector for transmission, represent “*Ca*. A. rohweri” hosted by interstitial organisms, or contain marine invertebrate eDNA [98]. “*Ca*. J. acanthamoeba” is horizontally transferred in aquatic environments [75]; both freshwater and seawater may thus play a role in transmission of “*Ca*. Aquarickettsia.” “*Ca*. A. rohweri” was absent in terrestrial samples, such as dust, groundwater, and “mammal-associated” data sets, including those from human medical samples (Fig. 6 gray bars). This analysis was repeated for OTUs within “*Ca*. Aquarickettsia” and compared to other OTUs from “*Ca*. Midichloriaceae” that were not part of the proposed genus (Supplementary Table S13).

**Fig. 7:**
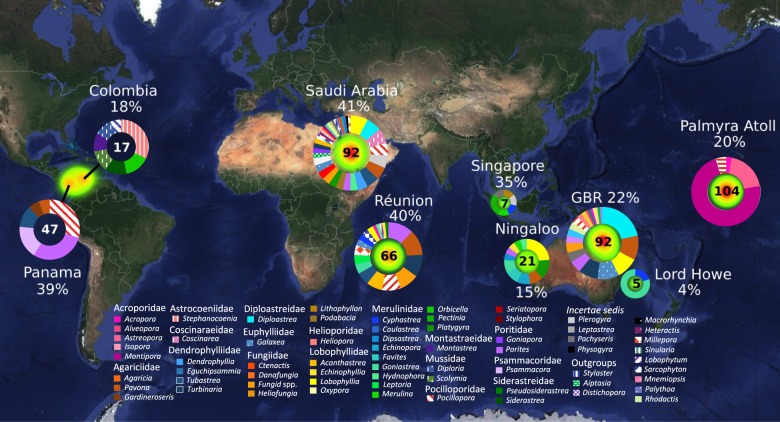
Map of the distribution of *“Ca*. A. rohweri” in coral and cnidarian outgroups. Coral samples included in map were from the Global Coral Microbiome Project (EMP Study ID 10895) and Palmyra Atoll Corallimorph and Bleaching Surveys (EMP Study ID 10798), a total of 451 samples. Ring charts show the number of samples (numeral inside ring) and the different coral host genera (exterior colored bars) in which *“Ca*. A. rohweri” was present. Percentages indicate the number of coral samples from each geographic location in which *“Ca*. A. rohweri” was identified. *“Ca*. A. rohweri” has also been identified in samples from the Caribbean which were sequenced using a different protocol and therefore not included in this analysis

Out of the 1401 total coral samples in the EMP database, 457 (32.62%) had OTUs identified as “*Ca*. A. rohweri” (i.e., >97% identity), whereas 494 (38.2%) had OTUs within “*Ca*. Aquarickettsia.” As metadata for coral samples in the EMP database included sampling coordinates and host identity, we had the capability to determine how “*Ca*. A. rohweri” was distributed globally and in which coral species it was identified (Fig. 7). We found that this OTU is present in the Pacific, Atlantic, and Indian Oceans in 51 scleractinian genera, 11 cnidarian outgroups, and a ctenophore, *Mnemiopsis*.

When we mapped the distribution of EMP sponge samples (primarily from the Sponge Microbiome Project [99]) in which “*Ca*. A. rohweri” was identified (Fig. S12), we found an even more global distribution, with samples spanning 76 sponge genera. “*Ca*. A. rohweri” was identified in sponge samples across the world, and is widespread throughout Europe (North Sea, Northern Atlantic, Mediterranean Sea), the Red Sea, the Caribbean, and Australia.

To supplement results from analysis of the EMP database, we queried the SRA database with the full-length 16S rRNA sequence of “*Ca*. A. rohweri” using IMNGS at 97% and 99% similarity thresholds (Supplementary Table S14). While metadata for the SRA database is less detailed than the EMP database, these results indicated that sequences identified as “*Ca*. A. rohweri” were found exclusively in marine metagenomes, seven of which were coral metagenomes. Although there were 12,665 marine metagenomes (including seawater, marine sediment, and plankton metagenomic samples) and 1855 coral metagenomes queried, “*Ca*. A. rohweri” was only found in 21 samples. This is likely due to our use of the full-length 16S rRNA sequence for this analysis, which is more informative than an 100 -bp fragment.

Together these data suggest that the proposed genus “*Ca*. Aquarickettsia” broadly associates with corals and with many members of the non-bilaterian metazoan phyla (Placozoa, Porifera, Cnidaria, and Ctenophora), as well as the even more ancient protists. This broad host range is not uncommon in Rickettsiales: many species of this family (notably, *Wolbachia* spp.) are able to switch hosts, infecting both vertebrate and invertebrate hosts while still maintaining virulence [75, 100].

### “Candidatus Aquarickettsia” may drive disease events under nutrient-replete conditions

**Fig. 8:**
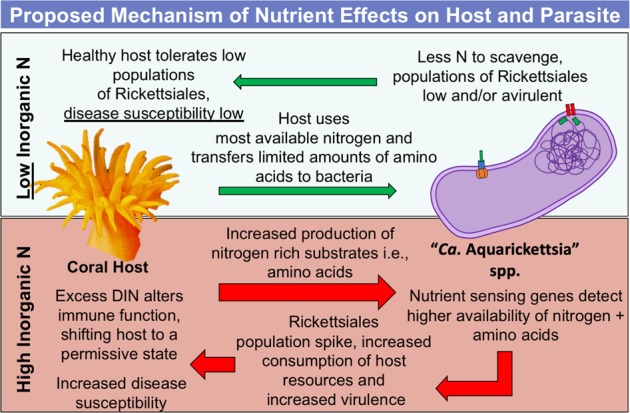
Model of predicted interactions between species of “*Candidatus* Aquarickettsia” with invertebrate host and how interactions are altered by nutrient input

We characterized the genome and metabolic capabilities of “*Ca*. A. rohweri,” a novel bacterium in a new genus of Rickettsiales that is found globally distributed in corals and sponges and also associates with protists and placozoans. As its proposed name suggests, the genus “*Ca*. Aquarickettsia” is primarily associated with marine-dwelling organisms. Based on the metabolic capabilities of “*Ca*. A. rohweri,” we postulate that this group of parasitic organisms plays a role in coral disease development. Previously, we reported evidence that inorganic nutrient exposure increased “*Ca*. A. rohweri” populations, reduced coral growth [3], and increased host tissue loss and mortality [2]. Based on this evidence and the presence of nutrient-sensing genes, we hypothesize that exposure to nitrate and ammonium leads to a transition in the role of “*Ca*. A. rohweri” from commensal to parasitic in coral hosts (Fig. 8). We hypothesize that “*Ca*. A. rohweri” detects nutrient enrichment through NtrY-NtrX, triggering other functions such as cell growth and division or the upregulation of transporters to acquire more metabolites from the host. As nutrient enrichment also allows the coral host and algal symbiont to produce more N-rich amino acids and proteins, surplus resources could easily be scavenged by the parasite. Last, we hypothesize that in nutrient-replete conditions, the presence of surplus nitrogen metabolites encourages the proliferation of this parasite to levels that may overwhelm host metabolic capabilities (Fig. 8).

Rickettsiales have been implicated in coral disease for more than 25 years. However, their presence in seemingly healthy corals has led to doubt that they are the primary pathogen responsible for white-band disease. We show here that “*Ca*. A rohweri” is globally associated with many coral hosts, and possesses the genomic capacity to parasitize the coral holobiont for amino acids and ATP. We also previously showed that in response to nutrient enrichment, these parasites proliferate and reduce coral growth. Thus through shifts in its population dynamics “*Ca*. A rohweri” may leave the host more susceptible to other opportunistic pathogens leading to disease. Alternatively, “*Ca*. A rohweri” may serve as a primary pathogen, although this still remains speculative. As nutrient pollution increasingly affects reefs, we suspect that parasites within the proposed genus will proliferate and may contribute to heightened vulnerability to disease and mortality.

## Supplementary information

Supplementary Tables

Supplementary Figure S1

Supplementary Figure S2

Supplementary Figure S3

Supplementary Figure S4

Supplementary Figure S5

Supplementary Figure S6

Supplementary Figure S7

Supplementary Figure S8

Supplementary Figure S9

Supplementary Figure S10

Supplementary Figure S11

Supplementary Figure S12
