# Supplementary material for: Phylogenetic, genomic, and biogeographic characterization of a novel and ubiquitous marine invertebrate-associated Rickettsiales parasite, Candidatus Aquarickettsia rohweri, gen. nov., sp. nov
Source: ISME J. 2019 Aug 5;13(12):2938–53. doi: 10.1038/s41396-019-0482-0 (PMC6863919; doi:10.1038/s41396-019-0482-0)
Supplement: Supplementary file 1 — Supplementary Tables [file 41396_2019_482_MOESM1_ESM.pdf]

Supplementary Table S1. A. Adaptor and index sequences used for shotgun metagenomic sequencing on Illumina MiSeq.

Adaptor Sequences	GTCTCGTGGGCTCGGAGATGTGTATAAGAGACAG, TCGTCGGCAGCGTCAGATGTGTATAAGAGACAG, AATGATACGGCGACCACCGAGATCTACACCTCTCTATTCG TCGGCAGCGTC, CAAGCAGAAGACGGCATACGAGATAGGCAGAAGTCTCG TGGGCTCGG
Index Sequences	AGGCAGAA (N703) and CTCTCTAT (S502)

Supplementary Table S1. B. Nested primer sets used to amplify the 16S rRNA sequences from multiple placozoan species.

Long product Forward primer (Midi_16S_F1): 5’-GCAAACGGGTGAGTAATACATGG-3’	(used for PCR and sequencing)
Long product Reverse primer (Midi_16S_R1): 5’-CAAACTCTCCTTGGTAAACTGCC-3’	(used for PCR and sequencing)
Nested Forward primer (Midi_16S_F2): 5’-AGATATCAGGAGGAATATCGAAGGC-3’	(used for sequencing)
Nested Reverse primer (Midi_16S_R2): 5’-ACAGCGTCAGTATTTAGCCAGAC-3’	(used for sequencing)

Supplementary Table S2. Parameters used for filtering and trimming of low-quality sequences and removal of adaptor sequences with FQtrim.

Disable automatic poly A/T trimming at read ends	true (-A)
Enable quality trimming, trim 3' end of read when average phred score drops below (minqv)	30 (-q 30), default sliding window size of 6
Minimum read length allowed	100 (-l 100)
Minimum length of suffix-prefix overlap between read and adapter that can be trimmed at read end	6 (default)
Maximum percentage of Ns allowed in a read after trimming (By default FQtrim trims the end of reads if they have Ns at that end)	5 (default)
Apply low-complexity (dust) filter and discard any read that has over 50% of its length detected as low complexity	true (-D)

Supplementary Table S3. Reference genomes used with Bowtie to remove contamination. Default Bowtie parameters were used except mismatch penalty was set to 5.

Symbiodinium spp.	Symbiodinium microadriaticum strain KB8	NCBI Tax ID: 230985 (JGI)
Acropora digitifera	assembly Adig_1.1 (scaffold level, not complete)	GCA_000222465.2 Adig_1.1
Human	Genome Reference Consortium Human GRCh38	GCA_000001405.15

Supplementary Table S4. Results from CheckM marker gene evaluation.

Genome Size	No. of Scaffolds	N50	Mean Contig Length	Longest Contig	GC Content
1284848 (1.28 Mb)	155	10860 bp	8289 bp	35498 bp	27.59%

No. of Predicted Genes	Contamination	Completeness	Coding Density
1469	0.39%	97.30%	87.65%

Supplementary Table S5. Organisms selected for BLAST comparison for optimal annotation by the KAAS server.

Abbrev.	Organism	Number of Sequences
hsa	Homo sapiens (human)	20,338
dme	Drosophila melanogaster (fruit fly)	13,929
ath	Arabidopsis thaliana (thale cress)	27,635
sce	Saccharomyces cerevisiae (budding yeast)	6,002
pfa	Plasmodium falciparum 3D7	5,307
eco	Escherichia coli K-12 MG1655	4,140
sty	Salmonella enterica subsp. enterica serovar Typhi CT18	4,473
hin	Haemophilus influenzae Rd KW20 (serotype d)	1,610
pae	Pseudomonas aeruginosa PAO1	5,572
nme	Neisseria meningitidis MC58 (serogroup B)	1,943
hpy	Helicobacter pylori 26695	1,445
mlo	Mesorhizobium japonicum MAFF 303099	7,281
bsu	Bacillus subtilis subsp. subtilis 168	4,174
sau	Staphylococcus aureus subsp. aureus N315 (MRSA/VSSA)	2,624
lla	Lactococcus lactis subsp. lactis Il1403	2,277
spn	Streptococcus pneumoniae TIGR4 (virulent serotype 4)	2,125
cac	Clostridium acetobutylicum ATCC 824	3,778
mge	Mycoplasma genitalium G37	476
mtu	Mycobacterium tuberculosis H37Rv	3,906
ctr	Chlamydia trachomatis D/UW-3/CX	887
bbu	Borrelia burgdorferi B31	1,391
syn	Synechocystis sp. PCC 6803	3,564
aae	Aquifex aeolicus	1,526
mja	Methanocaldococcus jannaschii	1,770
afu	Archaeoglobus fulgidus DSM 4304	2,407
pho	Pyrococcus horikoshii	2,061
ape	Aeropyrum pernix	1,700
rty	Rickettsia typhi Wilmington	838
rcm	Rickettsia canadensis McKiel	1,093
rbe	Rickettsia bellii RML369-C	1,429
rbo	Rickettsia bellii OSU 85-389	1,476
rco	Rickettsia conorii	1,374
rfe	Rickettsia felis	1,512
rak	Rickettsia akari	1,259
rri	Rickettsia rickettsii Sheila Smith	1,345
rrj	Rickettsia rickettsii Iowa	1,384
rpk	Rickettsia massiliae MTU5	947
rms	Rickettsia peacockii	980
raf	Rickettsia africae	1,041
mmn	Candidatus Midichloria mitochondrii	1,210
**Total**		150,107

Supplementary Table S6. Unique genes and gene clusters belonging to each of the organisms used for OrthoFinder orthologous gene analysis

Organism	Unique genes	Unique Gene Clusters
Anaplasma marginale str Florida	58	2
Anaplasma ovis str Haibei	63	2
Anaplasma phagocytophilum str HZ	164	4
Candidatus Fokinia solitaria	132	0
Candidatus Jidaibacter acanthamoeba	575	5
Candidatus Aquarickettsia rohweri	334	2
Candidatus Midichloria mitochondrii	406	1
Candidatus Neoehrlichia lotoris str RAC413	80	2
Ehrlichia canis str Jake	76	0
Ehrlichia chaffeensis str Arkansas	24	0
Ehrlichia sp HF	21	2
Neorickettsia helminthoeca str Oregon	52	1
Neorickettsia risticii str Illinois	22	0
Neorickettsia sennetsu str Miyayama	17	1
Rickettsia bellii str RML An4	211	2
Rickettsia conorii Malish7	72	0
Rickettsia prowazekii str Madrid E	20	0
Rickettsia rickettsii str Iowa	92	0
Wolbachia endosymbiont Drosophila melanogaster ASM802v1	16	0
Wolbachia pipientis ASM175266v1	6	0
Wolbachia sp wRi	127	0

Supplementary Table S7. Parameters selected for genome circularization using CGView.

Global Blast Settings	query_split_size=10000, overlap_split_size=100
Blast 1	GCA_000219355.1_ASM21935v1_genomic.fna, blastn, expect=1, Bacterial and Plant Plastid, filter=Yes
Show GenBank/EMBL features:	Yes
Show GC content	Yes
Show divider rings	Yes
Use opacity for BLAST hits	Yes
Show labels	No
Show ORFs (over 100 codons) and display them combined	Yes

Supplementary Table S8. Collected placozoan strains used for the amplification of RLO 16S rRNA sequence.

SWIMS=Swire Institute of Marine Science, The University of Hong Kong; STRI=Smithsonian Tropical Research Institute; SBR=Station Biologique de Roscoff.

Placozoan species/haplotypes were identified as described (M. Eitel and Schierwater 2010; Michael Eitel et al. 2013)

Placozoan species	Strain ID	Sampling location	Reference
Trichoplax adhaerens	GRELL	Elat, Israel	(Grell and Benwitz 1971)
Trichoplax sp. H2	ALPHA	Hong Kong: Outdoor tanks at SWIMS	This study
Trichoplax sp. H2	OKHD	Okinawa, Japan: 26.192260N 127.451455E	(M. Eitel and Schierwater 2010)
Trichoplax sp. H2	P1.2-2-S1	Hong Kong: 22.122488N 114.152511E	This study
Trichoplax sp. H2	PAN	Bocas del Toro, Panama: aquarium at the STRI	(Voigt et al. 2004)
Trichoplax sp. H2	ROS	Roscoff, France: flow-through aquarium system at SBR	(von der Chevallerie, Eitel, and Schierwater 2010; M. Eitel and Schierwater 2010)
Trichoplax sp. H2	T143C-1	Hong Kong: Outdoor tanks at SWIMS	This study
Trichoplax sp. H2	T145A-39	Hong Kong: Outdoor tanks at SWIMS	This study
Trichoplax sp. H2	T145A-48	Hong Kong: Outdoor tanks at SWIMS	This study
Trichoplax sp. H2	T162A-6	Hong Kong: Outdoor tanks at SWIMS	This study
Trichoplax sp. H2	T162C-1	Hong Kong: Outdoor tanks at SWIMS	This study
Trichoplax sp. H2	T162C-3	Hong Kong: Outdoor tanks at SWIMS	This study
Trichoplax sp. H17	P2.3-3-F.a11	Hong Kong: 22.193072N 114.173768E	This study
Hoilungia hongkongensis	M2RS3-2	22.352728N 114.251733E	(Michael Eitel et al. 2018)
Hoilungia sp. H15	M2RS3-11	22.352728N 114.251733E	This study

Supplementary Table S9. Accession numbers of sequences used to estimate 16S rRNA phylogeny. Version 1 if unspecified.

**Outgroup (11)**	**Accession No.**
Azospirillum soli strain CC-LY788	NR_145884
Lacibacterium aquatile strain LTC-2 16S	NR_125556
Pelagibius litoralis strain CL-UU02	NR_043785
Magnetospira thiophila strain MMS-1	NR_116475
Oceanibaculum pacificum strain MC2UP-L3	NR_116679
Rhodospira trueperi strain 8316	NR_036971.2
Planktomarina temperata strain RCA23	NR_117309
Temperatibacter marinus strain 5-11	NR_126192
Brucella melitensis biovar Melitensis strain 2000031283	NR_043003
Magnetococcus marinus strain MC-1	NR_074371
Holospora obtusa	HE797905
***Anaplasmataceae* (13)**	
Anaplasma marginale strain Florida	AF309867
Anaplasma ovis	AY262124
Anaplasma phagocytophilum strain Webster	NR_044762
"*Candidatus* Neoehrlichia lotoris" strain RAC413	EF633744
Ehrlichia canis strain Oklahoma	NR_118741
Ehrlichia chaffeensis strain Arkansas	NR_074500.2
Ehrlichia sp. HF	DQ647318
Neorickettsia helminthoeca	U12457
Neorickettsia risticii strain Illinois	NR_029162
Neorickettsia sennetsu strain Miyayama	NR_044746
Wolbachia endosymbiont of Drosophila melanogaster strain wMel	LC108848
Wolbachia pipientis	AY026913
Wolbachia sp. wRi	NC_012416
***Rickettsiaceae* (15)**	
Rickettsia rickettsii (strain R)	L36217
Rickettsia canadensis str. CA410	CP003304
Rickettsia slovaca str. D-CWPP	CP003375
Rickettsiaceae endosymbiont of Carteria cerasiformis rrs	LC004724
Rickettsia amblyommatis isolate An13	CP015012
Rickettsia japonica, strain YH_M	AP017602
Rickettsia africae ESF-5	CP001612
Rickettsia heilongjiangensis 054	CP002912
Rickettsia parkeri str. Portsmouth	CP003341
Rickettsia peacockii str. Rustic	CP001227
Rickettsia bellii	U11014
Rickettsia massiliae MTU5	CP000683
Candidatus Rickettsia amblyommii strain Ac37	CP012420

Rickettsia conorii strain Malish 7	NR_074480.2
Rickettsia prowazekii strain SDT1S14	JQ045833

“*Ca* . Midichloriaceae” (51)

Bacterium Sq1_host, strain Sq1 host	LN864514
Candidatus Lariskella arthropodarum clone NeLaSpr	JQ726733
Rickettsiales bacterium endosymbiont of *Nysius plebeius*	AB624350
Endosymbiont of *Acanthamoeba sp. UWC8*	CP004403
Alpha proteobacterium endosymbiont of *Acanthamoeba sp.*	KF924595
Lariskella endosymbiont of *Curculio morimotoi* , isolate P124_7	AB746413
Lariskella endosymbiont of *Curculio okumai* , isolate P123_1	AB746416
“*Candidatus* Jidaibacter acanthamoeba”	AF069962
“*Candidatus* Midichloria mitochondrii” IricVA	CP002130
“*Candidatus* Midichloria sp. Ixholo1”	FM992372
“*Candidatus* Anadelfobacter veles”, host *Euplotes harpa* strain HS11/'	FN552695
Rickettsiales bacterium It86	AF525482
“*Candidatus* Nicolleia massiliensis”	DQ788562
“*Candidatus* Cyrtobacter zanobii”	HE978250
“*Candidatus* Cyrtobacter comes” clone 121	FN552698
Lyticum sinuosum strain USBL-36I1, clone b	HF969040
Lyticum flagellatum strain 299	NR_125566
Rickettsiales bacterium Huangshan-1	AB297807
Candidatus Fokinia solitaria strain Rio ETE_ALG 3VII	KM497527
Uncultured alpha proteobacterium clone 0307_BHM2_29	JQ515644
Uncultured alpha proteobacterium clone 0907_Mf_HT1_B85	JQ516457
Uncultured alpha proteobacterium clone MD3.55	FJ425643
Uncultured alpha proteobacterium clone SGSH995	GQ347698
Uncultured bacterium clone Cc045	AY942762
Uncultured bacterium clone Gven_P15	GU118498
Uncultured bacterium clone Mfav_F04	GU118640
Uncultured bacterium clone Mfav_B15	GU118630
Uncultured bacterium clone Mfav_P11	GU118616
Uncultured bacterium clone B12_2	KU243155
Uncultured bacterium clone SING1046	HM128969
Uncultured bacterium clone FGL7S_B25	FJ437943
Uncultured bacterium clone Reef_O20	GU119441
Uncultured proteobacterium clone PEACE2006/237 _P3	EU394580
Uncultured Rickettsiales bacterium clone ID25L	EU555284
Uncultured Rickettsiales bacterium clone Ho(lakePloen)_13	EF667901
Uncultured bacterium clone 1301APX_F11	GU189017
Placozoa_H2ALPHA	
Placozoa_H1_GRELL	
Placozoa_H2_PAN	
Placozoa_H17_P2_3_2_F_a11	
Placozoa_H13_M2RS3_2	

Placozoa_H15_M2RS3_11
Placozoa_H2_OKHD
Placozoa_H2_ROS
Placozoa_H2_T162C_3
Placozoa_H2_T162C_1
Placozoa_H2_T162A_6
Placozoa_H2_T145A_48
Placozoa_H2_T145A_39
Placozoa_H2_T143C_1
Placozoa_H2_P1_2_2_S1

Supplementary Table S10. Parameters used for PhyML based on results from jModelTest.

Data type:	dna
Alphabet size:	4
Sequence format:	interleaved
Number of data sets:	1
Nb of bootstrapped data sets:	1000
Compute approximate likelihood ratio test:	no
Model name:	Custom
Proportion of invariable sites:	estimated
Number of subst. rate categs:	4
Gamma distribution parameter:	estimated
'Middle' of each rate class:	mean
Tree topology search:	Best of NNIs and SPRs
Starting tree:	BioNJ
Add random input tree:	no
Optimize branch lengths:	yes
Optimize substitution model parameters:	yes
Run ID:	GTR+I+G
Random seed:	1496382477
Subtree patterns aliasing:	no
Version:	20120412
Nucleotide equilibrium frequencies:	ML
Optimise tree topology:	yes

Supplementary Table S11. List of conserved, single copy marker genes used for concatenated marker gene phylogeny.

Gene	COG category	COG ID	HMM profile	Function
alaS	J	COG0013	TIGR00344	Alanine-tRNA ligase
argS	J	COG0018	TIGR00456	Arginine-tRNA ligase
aspS	J	COG0173	TIGR00459	Aspartate-tRNA ligase
cgtA	DL	COG0536	TIGR02729/PF01018	GTPase ObgE/CgtA
coaE	H	COG0237	TIGR00152	Dephospho-CoA kinase
cysS	J	COG0215	TIGR00435	Cysteine-tRNA ligase
dnaA	L	COG0593	TIGR00362/PF00308	Chromosomal replication initiator protein DnaA
dnaG	L	COG0358	TIGR01391	DNA primase
dnaX	L	COG2812	TIGR02397	DNA polymerase III subunit gamma
engA	R	COG1160	TIGR03594	GTPase Der
ffh	U	COG0541	TIGR00959	Signal recognition particle protein
fmt	J	COG0223	TIGR00460	Methionyl-tRNA formyltransferase
frr	J	COG0233	TIGR00496	Ribosome-recycling factor
ftsY	U	COG0552	TIGR00064	Signal recognition particle receptor FtsY
gmk	F	COG0194	TIGR03263/PF00625	Guanylate kinase
hisS	J	COG0124	TIGR00442	Histidine-tRNA ligase
ileS	J	COG0060	TIGR00392	Isoleucine-tRNA ligase 1
infB	J	COG0532	TIGR00487	Translation initiation factor IF-2
infC	J	COG0290	TIGR00168	Translation initiation factor IF-3
ksgA	J	COG0030	TIGR00755	Ribosomal RNA small subunit methyltransferase A
lepA	J	COG0481	TIGR01393	Elongation factor 4
leuS	J	COG0495	TIGR00396	Leucine-tRNA ligase
ligA	L	COG0272	TIGR00575	DNA ligase
nusA	K	COG0195	TIGR01953	Transcription termination/antitermination protein NusA
nusG	K	COG0250	TIGR00922	Transcription termination/antitermination protein NusG
pgk	G	COG0126	PF00162	Phosphoglycerate kinase
pheS	J	COG0016	TIGR00468	Phenylalanine-tRNA ligase alpha subunit
pheT	J	COG0073	TIGR00472	Phenylalanine-tRNA ligase beta subunit
prfA	J	COG0216	TIGR00019	Peptide chain release factor 1

pyrG	F	COG0504	TIGR00337	CTP synthase
recA	L	COG0468	TIGR02012	DNA recombination and repair protein
rbfA	J	COG0858	TIGR00082	30S ribosome-binding factor
rnc	K	COG0571	TIGR02191	Ribonuclease 3
rplA	J	COG0081	TIGR01169	50S ribosomal protein L1
rplB	J	COG0090	TIGR01171	50S ribosomal protein L2
rplC	J	COG0087	TIGR03625/PF00297	50S ribosomal protein L3
rplD	J	COG0088	TIGR03953	50S ribosomal protein L4
rplE	J	COG0094	PF00281	50S ribosomal protein L5
rplF	J	COG0097	TIGR03654/PF00347	50S ribosomal protein L6
rplI	J	COG0359	TIGR00158/PF01281	50S ribosomal protein L9
rplJ	J	COG0244	PF00466	50S ribosomal protein L10
rplK	J	COG0080	TIGR01632	50S ribosomal protein L11
rplL	J	COG0222	TIGR00855	50S ribosomal protein L7/L12
rplM	J	COG0102	TIGR01066	50S ribosomal protein L13
rplN	J	COG0093	TIGR01067	50S ribosomal protein L14
rplO	J	COG0200	TIGR01071	50S ribosomal protein L15
rplP	J	COG0197	TIGR01164	50S ribosomal protein L16
rplQ	J	COG0203	TIGR00059	50S ribosomal protein L17
rplR	J	COG0256	TIGR00060	50S ribosomal protein L18
rplS	J	COG0335	TIGR01024	50S ribosomal protein L19
rplT	J	COG0292	TIGR01032	50S ribosomal protein L20
rplU	J	COG0261	TIGR00061	50S ribosomal protein L21
rplV	J	COG0091	TIGR01044	50S ribosomal protein L22
rplW	J	COG0089	PF00276	50S ribosomal protein L23
rplX	J	COG0198	TIGR01079	50S ribosomal protein L24
rpmA	J	COG0211	TIGR00062	50S ribosomal protein L27
rpmC	J	COG0255	TIGR00012	50S ribosomal protein L29
rpmI	J	COG0291	TIGR00001	50S ribosomal protein L35
rpoA	K	COG0202	TIGR02027	DNA-directed RNA polymerase subunit alpha
rpoB	K	COG0085	TIGR02013	DNA-directed RNA polymerase subunit beta
rpoC	K	COG0086	TIGR02386	DNA-directed RNA polymerase subunit beta’
rpsB	J	COG0052	TIGR01011	30S ribosomal protein S2
rpsC	J	COG0092	TIGR01009	30S ribosomal protein S3
rpsD	J	COG0522	TIGR01017	30S ribosomal protein S4
rpsE	J	COG0098	TIGR01021	30S ribosomal protein S5
rpsF	J	COG0360	TIGR00166/PF01250	30S ribosomal protein S6
rpsG	J	COG0049	TIGR01029	30S ribosomal protein S7

rpsH	J	COG0096	PF00410	30S ribosomal protein S8
rpsI	J	COG0103	PF00380	30S ribosomal protein S9
rpsJ	J	COG0051	TIGR01049	30S ribosomal protein S10
rpsK	J	COG0100	TIGR03632	30S ribosomal protein S11
rpsL	J	COG0048	TIGR00981	30S ribosomal protein S12
rpsM	J	COG0099	TIGR03631	30S ribosomal protein S13
rpsO	J	COG0184	TIGR00952	30S ribosomal protein S15
rpsP	J	COG0228	TIGR00002	30S ribosomal protein S16
rpsQ	J	COG0186	TIGR03635	30S ribosomal protein S17
rpsR	J	COG0238	TIGR00165	30S ribosomal protein S18
rpsS	J	COG0185	TIGR01050	30S ribosomal protein S19
rpsT	J	COG0268	TIGR00029	30S ribosomal protein S20
secA	U	COG0653	TIGR00963	Protein translocase subunit SecA
secG	U	COG1314	TIGR00810	Protein-export membrane protein SecG
secY	U	COG0201	TIGR00967	Protein translocase subunit SecY
serS	J	COG0172	TIGR00414	Serine-tRNA ligase
smpB	O	COG0691	TIGR00086	SsrA-binding protein
tig	O	COG0544	TIGR00115	Trigger factor
tilS	J	COG0037	TIGR02432	tRNA(Ile)-lysidine synthase
truB	J	COG0130	TIGR00431	tRNA pseudouridine synthase B
tsaD	J	COG0533	TIGR03723	tRNA N6-adenosine threonylcarbamoyltransferase
tsf	J	COG0264	TIGR00116/PF00889	Elongation factor Ts
uvrB	L	COG0556	TIGR00631	UvrABC system protein B
ybeY	J	COG0319	TIGR00043	Endoribonuclease YbeY
ychF	J	COG0012	TIGR00092	Ribosome-binding ATPase YchF

Supplementary Table 12. Homology to *rvh* genes from related species. While "*Ca.* A. rohweri" has annotations for all necessary components of the *rvh* T4SS, homology to other Rickettsiales varied between genes.

VirB4

Query acc.ver	Description	Subject acc.ver
Query_207686	VirB4 family type IV secretion/conjugal transfer ATPase [Candidatu	WP_126044685.1
Query_207686	VirB4 family type IV secretion/conjugal transfer ATPase [Candidatu	WP_039454670.1
Query_207686	Type IV secretion system ATPase VirB4 [Candidatus Jidaibacter aca	KIE06097.1
Query_207686	type IV secretion system ATPase VirB4 [endosymbiont of Acanthan	AIF81852.1
Query_207686	VirB4 family type IV secretion/conjugal transfer ATPase [endosymb	WP_038540303.1
Query_207686	VirB4 family type IV secretion/conjugal transfer ATPase [Rickettsia	WP_130122451.1
Query_207686	VirB4 family type IV secretion/conjugal transfer ATPase [Candidatu	WP_041185441.1
Query_207686	VirB4 family type IV secretion/conjugal transfer ATPase [Rickettsia	WP_125216218.1
Query_207686	ype IV secretion system protein VirB4 [Alphaproteobacteria bacteri	PIR37403.1
Query_207686	type IV secretion system protein VirB4 [Alphaproteobacteria bacte	OJV14206.1
Query_207686	VirB4 family type IV secretion/conjugal transfer ATPase [Rickettsia	WP_038604773.1
Query_207686	Type IV secretion system protein virB4 [Rickettsiales bacterium Ac	AIL65799.1
Query_207686	VirB4 family type IV secretion/conjugal transfer ATPase [Rickettsia	RYE06588.1
Query_207686	VirB4 family type IV secretion/conjugal transfer ATPase [Rickettsia	WP_064463020.1
Query_207686	VirB4 family type IV secretion/conjugal transfer ATPase [Rickettsia	WP_032073826.1
Query_207686	VirB4 family type IV secretion/conjugal transfer ATPase [Rickettsia	WP_016728033.1
Query_207686	VirB4 family type IV secretion/conjugal transfer ATPase [Rickettsia	WP_012719388.1
Query_207686	type IV secretion/conjugal transfer ATPase, VirB4 family protein [R	KJV81502.1
Query_207686	VirB4 [Rickettsia massiliae MTU5]	ABV84447.1
Query_207686	VirB4 family type IV secretion/conjugal transfer ATPase [Candidatu	WP_106874398.1
Query_207686	VirB4 family type IV secretion/conjugal transfer ATPase [Rickettsia	WP_012150361.1
Query_207686	VirB4 family type IV secretion/conjugal transfer ATPase [Rickettsia	WP_012013357.1
Query_207686	VirB4 family type IV secretion/conjugal transfer ATPase [Rickettsia	WP_014362738.1
Query_207686	VirB4 family type IV secretion/conjugal transfer ATPase [Rickettsia	WP_010976818.1
Query_207686	VirB4 family type IV secretion/conjugal transfer ATPase [Rickettsia	WP_057700336.1
Query_207686	VirB4 family type IV secretion/conjugal transfer ATPase [Rickettsia	WP_041404487.1
Query_207686	VirB4 family type IV secretion/conjugal transfer ATPase [Rickettsia	WP_016769883.1
Query_207686	VirB4 family type IV secretion/conjugal transfer ATPase [Rickettsia	WP_014408196.1
Query_207686	VirB4 family type IV secretion/conjugal transfer ATPase [Rickettsia	WP_004996711.1
Query_207686	VirB4 family type IV secretion/conjugal transfer ATPase [Rickettsia	WP_010423970.1
Query_207686	VirB4 family type IV secretion/conjugal transfer ATPase [Rickettsia	WP_062810989.1
Query_207686	VirB4 family type IV secretion/conjugal transfer ATPase [Rickettsia	WP_041077585.1
Query_207686	VirB4 family type IV secretion/conjugal transfer ATPase [Rickettsia	WP_014412013.1
Query_207686	MULTISPECIES: VirB4 family type IV secretion/conjugal transfer AT	WP_014365231.1
Query_207686	VirB4 family type IV secretion/conjugal transfer ATPase [Rickettsia	WP_016925946.1
Query_207686	VirB4 family type IV secretion/conjugal transfer ATPase [Rickettsia	WP_064429383.1
Query_207686	VirB4 family type IV secretion/conjugal transfer ATPase [Rickettsia	WP_040256884.1
Query_207686	VirB4 family type IV secretion/conjugal transfer ATPase [Rickettsia	WP_032139312.1

Query_207686	VirB4 family type IV secretion/conjugal transfer ATPase [Rickettsia	WP_014273169.1
Query_207686	VirB4 family type IV secretion/conjugal transfer ATPase [Rickettsia	WP_014120508.1
Query_207686	VirB4 family type IV secretion/conjugal transfer ATPase [Rickettsia	WP_016917606.1
Query_207686	VirB4 family type IV secretion/conjugal transfer ATPase [Rickettsia	WP_012736626.1
Query_207686	VirB4 family type IV secretion/conjugal transfer ATPase [Rickettsia	WP_014391789.1
Query_207686	VirB4 family type IV secretion/conjugal transfer ATPase [Rickettsia	WP_014364395.1
Query_207686	VirB4 family type IV secretion/conjugal transfer ATPase [Rickettsia	WP_014013852.1
Query_207686	VirB4 [Rickettsia amblyommatis]	ACA97599.1
Query_207686	VirB4 family type IV secretion/conjugal transfer ATPase [Rickettsia	WP_014409876.1
Query_207686	VirB4 family type IV secretion/conjugal transfer ATPase [Rickettsia	WP_103897324.1
Query_207686	VirB4 family type IV secretion/conjugal transfer ATPase [Rickettsia	WP_045805285.1
Query_207686	VirB4 family type IV secretion/conjugal transfer ATPase [Rickettsia	WP_045804223.1
Query_207686	VirB4 family type IV secretion/conjugal transfer ATPase [Rickettsia	WP_017443436.1
Query_207686	MULTISPECIES: VirB4 family type IV secretion/conjugal transfer AT	WP_016948317.1
Query_207686	VirB4 family type IV secretion/conjugal transfer ATPase [Rickettsia	WP_011270442.1
Query_207686	Type IV secretion system protein VirB4 [Rickettsia monacensis IrR/	CDI28941.1
Query_207686	VirB4 family type IV secretion/conjugal transfer ATPase [Rickettsia	WP_046058379.1
Query_207686	VirB4 family type IV secretion/conjugal transfer ATPase [Rickettsia	WP_037214007.1
Query_207686	VirB4 family type IV secretion/conjugal transfer ATPase [Wolbachia	WP_096617108.1
Query_207686	VirB4 family type IV secretion/conjugal transfer ATPase [Rickettsia	WP_004596464.1
Query_207686	VirB4 family type IV secretion/conjugal transfer ATPase [Rickettsia	WP_014410351.1
Query_207686	VirB4 family type IV secretion/conjugal transfer ATPase [Rickettsia	WP_011477919.1
Query_207686	VirB4 family type IV secretion/conjugal transfer ATPase [Rickettsia	WP_012148251.1
Query_207686	VirB4 family type IV secretion/conjugal transfer ATPase [Rickettsia	WP_011190508.1
Query_207686	VirB4 family type IV secretion/conjugal transfer ATPase [Wolbachia	WP_017532093.1
Query_207686	VirB4 family type IV secretion/conjugal transfer ATPase [Rickettsia	WP_004599743.1
Query_207686	VirB4 family type IV secretion/conjugal transfer ATPase [Rickettsia	WP_094648641.1
Query_207686	MULTISPECIES: VirB4 family type IV secretion/conjugal transfer AT	WP_052264802.1
Query_207686	MULTISPECIES: VirB4 family type IV secretion/conjugal transfer AT	WP_007302030.1
Query_207686	VirB4 family type IV secretion/conjugal transfer ATPase [Wolbachia	WP_010402460.1
Query_207686	VirB4 family type IV secretion/conjugal transfer ATPase [Orientia t	WP_109234621.1
Query_207686	VirB4 family type IV secretion/conjugal transfer ATPase [Orientia t	WP_109490184.1
Query_207686	VirB4 family type IV secretion/conjugal transfer ATPase [Orientia t	WP_012461381.1
Query_207686	VirB4 family type IV secretion/conjugal transfer ATPase [Wolbachia	WP_064125158.1
Query_207686	VirB4 family type IV secretion/conjugal transfer ATPase [Wolbachia	WP_127463754.1
Query_207686	VirB4 family type IV secretion/conjugal transfer ATPase [Wolbachia	WP_063630671.1
Query_207686	transporter [Rickettsia endosymbiont of Culicoides newsteadi]	OZG32541.1
Query_207686	VirB4 family type IV secretion/conjugal transfer ATPase [Wolbachia	WP_077188210.1
Query_207686	VirB4 family type IV secretion/conjugal transfer ATPase [Wolbachia	WP_068652416.1
Query_207686	VirB4 family type IV secretion/conjugal transfer ATPase [Orientia t	WP_064591397.1
Query_207686	type IV secretion/conjugal transfer ATPase, VirB4 family protein [C	KJW01846.1
Query_207686	VirB4 family type IV secretion/conjugal transfer ATPase [Wolbachia	WP_006015049.1
Query_207686	VirB4 family type IV secretion/conjugal transfer ATPase [Orientia t	WP_045914472.1

Query_207686	VirB4 family type IV secretion/conjugal transfer ATPase [Wolbachi	WP_095742766.1
Query_207686	type IV secretion system protein VirB4 [Wolbachia endosymbiont c	EAL59243.1
Query_207686	VirB4 family type IV secretion/conjugal transfer ATPase [Orientia t	WP_011945113.1
Query_207686	MULTISPECIES: VirB4 family type IV secretion/conjugal transfer AT	WP_010962886.1
Query_207686	VirB4 family type IV secretion/conjugal transfer ATPase [Wolbachi	WP_064085841.1
Query_207686	VirB4 family type IV secretion/conjugal transfer ATPase [Rickettsia	RTK92049.1
Query_207686	VirB4 family type IV secretion/conjugal transfer ATPase [Wolbachi	WP_110409798.1
Query_207686	VirB4 family type IV secretion/conjugal transfer ATPase [Wolbachi	WP_015589249.1
Query_207686	VirB4 family type IV secretion/conjugal transfer ATPase [Wolbachi	WP_014868751.1
Query_207686	VirB4 family type IV secretion/conjugal transfer ATPase [Wolbachi	WP_038199157.1
Query_207686	vir-like protein B4 [Wolbachia pipientis]	ABI50362.1
Query_207686	VirB4 family type IV secretion/conjugal transfer ATPase [Wolbachi	WP_041046208.1
Query_207686	MULTISPECIES: VirB4 family type IV secretion/conjugal transfer AT	WP_065094409.1
Query_207686	VirB4 family type IV secretion/conjugal transfer ATPase [Wolbachi	WP_015587902.1
Query_207686	VirB4 family type IV secretion/conjugal transfer ATPase [Candidatu	WP_108672978.1
Query_207686	VirB4 family type IV secretion/conjugal transfer ATPase [Rickettsia	WP_017208453.1
Query_207686	VirB4 family type IV secretion/conjugal transfer ATPase [Wolbachi	WP_025264267.1
Query_207686	VirB4 [Wolbachia endosymbiont of Armadillidium vulgare]	AAX86701.1
Query_207686	VirB4 family type IV secretion/conjugal transfer ATPase [Orientia c	WP_045796973.1

% Identity	Alignment le	Mismatches	Gap opens	q. start	q. end	s. start
100	740	0	0	1	740	1
69.418	739	226	0	1	739	1
69.418	739	226	0	1	739	2
69.012	739	229	0	1	739	2
69.012	739	229	0	1	739	1
68.108	740	236	0	1	740	2
69.459	740	222	2	1	739	2
63.208	742	267	3	2	739	3
60.352	739	290	3	1	739	2
60.162	743	288	4	4	740	7
59.946	744	291	3	1	739	1
59.812	744	292	3	1	739	2
61.019	726	275	4	21	740	21
60.322	746	288	4	1	740	1
60.188	746	289	5	1	740	1
60.188	746	289	5	1	740	1
60.322	746	288	5	1	740	1
60.456	746	287	5	1	740	1
60.054	746	290	5	1	740	10
59.649	741	294	4	2	740	3
60.188	746	289	5	1	740	1
60.322	746	288	5	1	740	1
60.188	746	289	5	1	740	1
60.188	746	289	5	1	740	1
60.054	746	290	5	1	740	1
60.054	746	290	5	1	740	1
60.054	746	290	5	1	740	1
60.054	746	290	5	1	740	1
60.054	746	290	5	1	740	1
60.59	746	286	5	1	740	1
60.054	746	290	5	1	740	1
60.322	746	288	5	1	740	1
60.456	746	287	5	1	740	1
60.054	746	290	5	1	740	1
59.92	746	291	5	1	740	1
60.054	746	290	5	1	740	1
60.188	746	289	5	1	740	1
60.188	746	289	5	1	740	1

60.188	746	289	5	1	740	1
60.054	746	290	5	1	740	1
60.054	746	290	5	1	740	1
60.188	746	289	5	1	740	1
60.054	746	290	5	1	740	1
60.054	746	290	5	1	740	1
60.054	746	290	5	1	740	1
60.054	746	290	5	1	740	1
60.322	746	288	5	1	740	1
60.054	746	290	5	1	740	1
59.92	746	291	5	1	740	1
60.456	746	287	5	1	740	1
60.188	746	289	5	1	740	1
59.92	746	291	5	1	740	1
60.188	746	289	5	1	740	1
60.188	746	289	5	1	740	1
60.054	746	290	5	1	740	1
60.054	746	290	5	1	740	1
59.054	740	300	3	1	739	2
59.92	746	291	5	1	740	1
59.651	746	293	5	1	740	1
59.786	746	292	5	1	740	1
60.054	746	290	5	1	740	1
59.786	746	292	5	1	740	1
59.324	740	298	3	1	739	2
59.786	746	292	5	1	740	1
58.658	745	301	4	1	740	1
59.459	740	297	3	1	739	2
59.324	740	298	3	1	739	2
59.324	740	298	3	1	739	2
59.06	745	298	4	1	740	1
59.195	745	297	4	1	740	1
59.06	745	298	4	1	740	1
58.649	740	303	3	1	739	2
59.324	740	298	3	1	739	2
59.189	740	299	3	1	739	2
58.523	745	302	4	1	740	2
58.514	740	304	3	1	739	2
59.054	740	300	3	1	739	2
59.06	745	298	4	1	740	1
59.06	745	298	4	1	740	1
59.054	740	300	3	1	739	2
58.926	745	299	4	1	740	1

58.514	740	304	3	1	739	2
58.514	740	304	3	1	739	2
59.06	745	298	4	1	740	1
58.514	740	304	3	1	739	2
59.189	740	299	3	1	739	2
58.468	744	302	3	2	740	3
58.514	740	304	3	1	739	2
58.514	740	304	3	1	739	2
58.649	740	303	3	1	739	2
58.378	740	305	3	1	739	2
58.378	740	305	3	1	739	2
58.378	740	305	3	1	739	2
58.378	740	305	3	1	739	2
59.054	740	300	3	1	739	2
59.704	742	285	4	2	740	3
59.651	746	292	6	1	740	1
58.378	740	305	3	1	739	2
58.514	740	304	3	1	739	2
58.389	745	303	3	1	740	1

s. end	evalue	Bit score	% Positives
740	0	1534	100
739	0	1113	83.36
740	0	1113	83.36
740	0	1108	83.22
739	0	1107	83.22
741	0	1097	82.7
738	0	1090	82.7
742	0	985	79.51
737	0	968	77.81
747	0	960	77.66
742	0	958	76.61
743	0	956	76.61
744	0	946	77.69
744	0	941	76.01
744	0	940	76.54
744	0	940	76.54
744	0	939	76.41
744	0	939	76.14
753	0	939	76.27
740	0	939	76.92
744	0	939	76.41
744	0	939	76.01
744	0	939	76.41
744	0	938	76.41
744	0	938	76.41
744	0	938	76.27
744	0	938	76.41
744	0	938	76.41
744	0	938	76.41
744	0	938	76.27
744	0	937	76.54
744	0	937	76.14
744	0	937	76.14
744	0	937	76.27
744	0	937	76.27
744	0	937	76.27
744	0	937	76.14
744	0	937	76.27

744	0	937	76.27
744	0	936	76.54
744	0	936	76.14
744	0	936	76.14
744	0	936	76.27
744	0	936	76.27
744	0	936	76.41
744	0	936	76.14
744	0	936	76.41
744	0	935	76.41
744	0	935	76.41
744	0	935	76.14
744	0	935	76.14
744	0	934	76.27
744	0	934	76.01
744	0	934	76.14
744	0	932	76.14
744	0	932	76.01
739	0	931	77.03
744	0	931	76.01
744	0	931	75.87
744	0	931	76.01
744	0	929	76.01
744	0	929	75.87
739	0	929	76.76
744	0	929	75.87
743	0	929	76.64
739	0	929	76.62
739	0	928	76.62
739	0	928	76.62
743	0	928	75.97
743	0	928	75.97
743	0	927	75.97
739	0	927	76.89
739	0	927	76.62
739	0	927	76.62
744	0	926	76.64
739	0	926	76.89
739	0	926	76.49
743	0	926	75.97
743	0	926	75.97
739	0	926	76.62
743	0	926	75.97

739	0	926	76.76
739	0	926	76.76
743	0	925	75.97
739	0	925	76.76
739	0	925	76.49
744	0	925	75.4
739	0	924	76.62
739	0	924	76.62
739	0	924	76.35
739	0	923	76.62
739	0	923	76.76
739	0	923	76.62
739	0	922	76.49
739	0	922	76.35
733	0	922	75.34
743	0	920	75.6
739	0	918	76.22
739	0	915	76.08
743	0	915	75.17

Supplementary Table 12. Homology to *rvh* genes from related species. While "*Ca.* A. rohweri" has annotations for all necessary components of the *rvh* T4SS, homology to other Rickettsiales varied between genes.

VirD4

Query acc.ver	Description	Subject acc.ver
WP_12604415	type IV secretion system protein VirD4 [Candidatus Marinoinvertebra	WP_126044153.1
WP_12604415	type IV secretion system protein VirD4 [Candidatus Jidaibacter acant	WP_084212936.1
WP_12604415	putative virD4 protein [Candidatus Jidaibacter acanthamoeba]	KIE04285.1
WP_12604415	type IV secretion system protein VirD4 [endosymbiont of Acanthamo	WP_084173792.1
WP_12604415	type IV secretion system protein VirD4 [Candidatus Midichloria mito	WP_083809601.1
WP_12604415	type IV secretion system protein VirD4 [Rickettsiales endosymbiont o	WP_130122148.1
WP_12604415	hypothetical protein [Rickettsiales bacterium Ac37b]	WP_038602655.1
WP_12604415	type IV secretion system protein VirD4 [Alphaproteobacteria bacteriu	OJV16012.1
WP_12604415	hypothetical protein [Rickettsiales endosymbiont of Stachyamoeba li	WP_125216082.1
WP_12604415	type IV secretion system protein VirD4 [Ehrlichia canis]	WP_011304147.1
WP_12604415	type IV secretion system protein VirD4 [Ehrlichia minasensis]	WP_129992600.1
WP_12604415	type IV secretion system protein VirD4 [Ehrlichia minasensis]	WP_045171224.1
WP_12604415	type IV secretion system protein VirD4 [Ehrlichia chaffeensis]	WP_006010243.1
WP_12604415	type IV secretion system protein VirD4 [Ehrlichia chaffeensis]	WP_011452363.1
WP_12604415	type IV secretion system protein VirD4 [Ehrlichia ruminantium]	WP_011255230.1
WP_12604415	type IV secretion system protein VirD4 [Ehrlichia ruminantium]	WP_065433890.1
WP_12604415	type IV secretion system protein VirD4 [Ehrlichia ruminantium]	WP_065432266.1
WP_12604415	type IV secretion system protein VirD4 [Ehrlichia ruminantium]	WP_011154709.1
WP_12604415	type IV secretion system protein VirD4 [Ehrlichia ruminantium]	WP_062850784.1
WP_12604415	type IV secretion system protein VirD4 [Ehrlichia ruminantium]	WP_065433517.1
WP_12604415	type IV secretion system protein VirD4 [Wolbachia endosymbiont of	WP_014869091.1
WP_12604415	type IV secretion system protein VirD4 [Wolbachia endosymbiont of	WP_025264059.1
WP_12604415	type IV secretion system protein VirD4 [Neorickettsia helminthoeca]	WP_038559904.1
WP_12604415	primase [Wolbachia pipientis]	WP_019236953.1
WP_12604415	type IV secretion system VirD4 protein [Wolbachia endosymbiont of	CAL47250.1
WP_12604415	type IV secretion system protein VirD4 [Wolbachia endosymbiont of	WP_010407285.1
WP_12604415	type IV secretion system protein VirD4 [Wolbachia endosymbiont of	WP_010404946.1
WP_12604415	type IV secretion system protein VirD4 [Wolbachia endosymbiont of	WP_108784160.1
WP_12604415	type IV secretion system protein VirD4 [Anaplasma ovis str. Haibei]	ASI48034.1
WP_12604415	type IV secretion system protein VirD4 [Candidatus Neoehrlichia loto	WP_045809395.1
WP_12604415	type IV secretion system protein VirD4 [Wolbachia pipientis]	WP_006012545.1
WP_12604415	type IV secretion system protein VirD4 [Wolbachia endosymbiont of	WP_064086004.1
WP_12604415	type IV secretion system protein VirD4 [Ehrlichia muris]	WP_024071658.1
WP_12604415	MULTISPECIES: type IV secretion system protein VirD4 [Ehrlichia]	WP_045805004.1
WP_12604415	type IV secretion system protein VirD4 [Wolbachia endosymbiont of	WP_063631199.1
WP_12604415	MULTISPECIES: type IV secretion system protein VirD4 [Wolbachia]	WP_007302613.1
WP_12604415	type IV secretion system protein VirD4 [Anaplasma marginale str. Gy	AGZ79267.1
WP_12604415	component of type IV secretion system [Wolbachia sp. wTai]	BAA97436.1
WP_12604415	type IV secretion system protein VirD4 [Wolbachia endosymbiont of	WP_127464240.1
WP_12604415	type IV secretion system protein VirD4 [Wolbachia endosymbiont of	WP_011256482.1

WP_12604415	type IV secretion system protein VirD4 [Wolbachia endosymbiont of	WP_052265036.1
WP_12604415	VirD4 [Wolbachia endosymbiont of Armadillidium vulgare]	AAX86709.2
WP_12604415	type IV secretion system protein VirD4 [Anaplasma marginale]	WP_037330823.1
WP_12604415	type IV secretion system protein VirD4 [Anaplasma marginale]	WP_023387067.1
WP_12604415	type IV secretion system protein VirD4 [Wolbachia endosymbiont of	WP_114517613.1
WP_12604415	type IV secretion system protein VirD4 [Anaplasma marginale]	WP_118895707.1
WP_12604415	type IV secretion system protein VirD4 [Anaplasma marginale]	WP_114211673.1
WP_12604415	type IV secretion system protein VirD4 [Wolbachia endosymbiont of	WP_088415172.1
WP_12604415	type IV secretion system protein VirD4 [Anaplasma centrale]	WP_012880285.1
WP_12604415	type IV secretion system protein VirD4 [Anaplasma marginale]	WP_118890728.1
WP_12604415	type IV secretion system protein VirD4 [Neorickettsia risticii]	WP_015816577.1
WP_12604415	MULTISPECIES: type IV secretion system protein VirD4 [Anaplasma]	WP_011114675.1
WP_12604415	type IV secretion system protein VirD4 [Anaplasma marginale]	WP_042893100.1
WP_12604415	type IV secretion system protein VirD4 [Anaplasma marginale]	WP_037352898.1
WP_12604415	type IV secretion system protein VirD4 [Neorickettsia sennetsu]	WP_011452121.1
WP_12604415	type IV secretion system protein VirD4 [Ehrlichia sp. HF]	WP_044195693.1
WP_12604415	type IV secretion system protein VirD4 [Wolbachia endosymbiont of	WP_015588554.1
WP_12604415	type IV secretion system protein VirD4 [Wolbachia endosymbiont of	WP_064125076.1
WP_12604415	primase [Wolbachia endosymbiont of Diaphorina citri]	WP_017532548.1
WP_12604415	type IV secretion system protein VirD4 [Neorickettsia sp. 179522]	WP_067979916.1
WP_12604415	type IV secretory system Conjugative DNA transfer family protein [Eh	KJV65772.1
WP_12604415	type IV secretion system protein VirD4 [Wolbachia endosymbiont of	WP_068651314.1
WP_12604415	type IV secretion system protein VirD4 [Wolbachia pipientis]	WP_096097178.1
WP_12604415	type IV secretion system protein VirD4 [Wolbachia pipientis]	WP_096616658.1
WP_12604415	type IV secretion system protein VirD4 [Anaplasma phagocytophilum	WP_044143784.1
WP_12604415	type IV secretion system protein VirD4 [Anaplasma phagocytophilum	WP_044105598.1
WP_12604415	type IV secretion system VirD4 protein [Wolbachia endosymbiont of	CAL47251.1
WP_12604415	type IV secretion system protein VirD4 [Anaplasma phagocytophilum	WP_011451400.1
WP_12604415	type IV secretion system component VirD4 [Anaplasma phagocytoph	WP_021800099.1
WP_12604415	type IV secretory system Conjugative DNA transfer family protein [Ca	KJV69362.1
WP_12604415	type IV secretion system protein VirD4 [Wolbachia pipientis]	WP_070064791.1
WP_12604415	type IV secretion system protein virD4 [Wolbachia endosymbiont of I	CAJ41436.1
WP_12604415	Conjugal transfer protein TraG [Candidatus Arcanobacter lacustris]	KKB96705.1
WP_12604415	type IV secretion system protein VirD4 [Orientia tsutsugamushi]	WP_064644231.1
WP_12604415	type IV secretion system protein VirD4 [Anaplasma phagocytophilum	WP_064670093.1
WP_12604415	type IV secretion system protein VirD4 [Anaplasma phagocytophilum	WP_064660095.1
WP_12604415	type IV secretion system component VirD4 [Orientia tsutsugamushi]	WP_047220078.1
WP_12604415	type IV secretion system protein VirD4 [Orientia tsutsugamushi]	WP_109227115.1
WP_12604415	type IV secretion system protein VirD4 [Anaplasma phagocytophilum	AFY26818.1
WP_12604415	MULTISPECIES: type IV secretion system protein VirD4 [Wolbachia]	WP_007549026.1
WP_12604415	Type IV secretion system protein VirD4 [Anaplasma phagocytophilum	CEG20749.1
WP_12604415	type IV secretory system Conjugative DNA transfer family protein [Ne	AHX11634.1
WP_12604415	type IV secretory system Conjugative DNA transfer family protein [Eh	AHX04561.1
WP_12604415	type IV secretory system Conjugative DNA transfer family protein [Ar	KJZ98613.1
WP_12604415	type IV secretion system protein VirD4 [Candidatus Midichloria mitoc	AEI88750.1

WP_12604415	type IV secretion system protein VirD4 [Orientia tsutsugamushi]	WP_108840314.1
WP_12604415	type IV secretion system component VirD4 [Orientia tsutsugamushi]	WP_045912172.1
WP_12604415	type IV secretion system protein VirD4 [Orientia tsutsugamushi]	WP_012462029.1
WP_12604415	type IV secretion system protein VirD4 [Anaplasma phagocytophilum	WP_044152943.1
WP_12604415	type IV secretion system protein VirD4 [Orientia tsutsugamushi]	SPR02169.1
WP_12604415	type IV secretion system protein VirD4 [Orientia tsutsugamushi]	WP_011944234.1
WP_12604415	type IV secretion system component VirD4 [Orientia tsutsugamushi]	WP_045914593.1
WP_12604415	type IV secretion system protein VirD4 [Orientia tsutsugamushi]	WP_109489707.1
WP_12604415	type IV secretion system protein VirD4 [Orientia tsutsugamushi]	WP_064591814.1
WP_12604415	type IV secretory system Conjugative DNA transfer family protein [A	KJV68600.1
WP_12604415	type IV secretory system Conjugative DNA transfer family protein [A	KJV60577.1
WP_12604415	type IV secretory system Conjugative DNA transfer family protein [A	KJV66510.1
WP_12604415	type IV secretion system protein VirD4 [Wolbachia pipientis]	WP_077188467.1
WP_12604415	vir-like protein D4 [Wolbachia pipientis]	ABI50360.1
WP_12604415	MULTISPECIES: type IV secretion system protein VirD4 [Wolbachia]	WP_065094645.1

% Identity	Alignment le	Mismatches	Gap opens	q. start	q. end	s. start
100	624	0	0	1	624	1
74.099	583	149	1	5	585	9
74.099	583	149	1	5	585	13
73.928	583	150	1	5	585	9
79.368	538	108	2	56	593	3
72.758	591	152	2	9	590	9
73.252	572	141	4	6	571	2
71.034	580	163	3	9	585	8
69.1	589	176	5	3	588	2
66.24	625	181	5	5	620	6
66.721	619	185	4	5	615	6
66.937	617	183	4	5	613	6
68.547	585	172	2	5	581	6
68.547	585	172	2	5	581	6
67.617	596	187	2	5	594	6
67.617	596	187	2	5	594	6
67.617	596	187	2	5	594	6
67.617	596	187	2	5	594	6
67.617	596	187	2	5	594	6
67.617	596	187	2	5	594	6
64.8	625	216	2	1	621	1
64.64	625	217	2	1	621	1
70.415	578	149	3	8	572	4
65.7	621	206	4	5	621	4
64.48	625	218	2	1	621	1
65.539	621	207	4	5	621	4
65.539	621	207	4	5	621	4
65.378	621	208	4	5	621	4
66.828	618	189	6	1	605	1
71.178	569	156	4	6	568	7
65.539	621	207	4	5	621	4
65.539	621	207	4	5	621	4
69.019	581	176	1	5	581	6
64.865	629	212	2	5	624	6
65.378	621	208	4	5	621	4
65.378	621	208	4	5	621	4
67.608	602	183	3	1	591	1
65.378	621	208	4	5	621	4
65.217	621	209	4	5	621	4
67.055	601	192	3	1	597	1

65.378	621	208	4	5	621	4
64.734	621	212	3	5	621	4
67.608	602	183	3	1	591	1
68.007	597	179	3	1	586	1
64.895	621	211	4	5	621	4
68.007	597	179	3	1	586	1
68.007	597	179	3	1	586	1
65.902	610	203	3	1	606	1
67.608	602	183	3	1	591	1
68.007	597	179	3	1	586	1
67.219	604	189	3	8	606	4
68.007	597	179	3	1	586	1
68.007	597	179	3	1	586	1
68.007	597	179	3	1	586	1
66.557	607	188	3	8	606	4
68.675	581	178	1	5	581	6
65.169	623	206	5	5	621	4
66.172	606	201	2	5	606	4
64.734	621	212	3	5	621	4
67.384	604	188	3	8	606	4
71.481	547	151	1	83	624	2
64.687	623	209	5	5	621	4
65.217	621	209	4	5	621	4
66.007	606	202	2	5	606	4
67.78	599	177	8	5	596	4
65.756	622	194	9	5	619	4
67.174	591	190	2	1	587	1
67.78	599	177	8	5	596	4
67.78	599	177	8	5	596	4
78.024	496	109	0	73	568	43
64.107	638	186	8	1	624	1
64.677	620	215	2	5	620	5
70.37	567	151	5	8	568	5
69.841	567	170	1	2	567	4
67.613	599	178	8	5	596	4
67.446	599	179	8	5	596	4
69.841	567	170	1	2	567	4
69.665	567	171	1	2	567	4
67.446	599	179	8	5	596	4
65.512	606	205	2	5	606	4
68.197	588	172	7	16	596	4
77.53	494	111	0	79	572	3
75.835	509	123	0	73	581	42
68.197	588	172	7	16	596	4
84.211	456	71	1	138	593	1

69.489	567	172	1	2	567	4
69.312	567	173	1	2	567	4
69.312	567	173	1	2	567	4
69.338	574	166	6	5	571	4
69.489	567	172	1	2	567	4
69.312	567	173	1	2	567	4
69.312	567	173	1	2	567	4
69.312	567	173	1	2	567	4
69.312	567	173	1	2	567	4
72.14	542	139	4	61	596	38
72.14	542	139	4	61	596	26
72.14	542	139	4	61	596	38
65.017	606	208	2	5	606	4
65.017	606	208	2	5	606	4
65.017	606	208	2	5	606	4

s. end	evalue	Bit score	% Positives
624	0	1290	100
591	0	908	84.56
595	0	907	84.56
591	0	905	84.56
537	0	899	88.1
599	0	899	84.43
567	0	867	84.44
585	0	858	83.1
587	0	845	81.49
609	0	833	77.6
611	0	833	78.68
609	0	832	78.77
586	0	830	80.68
586	0	830	80.68
601	0	829	80.54
601	0	829	80.54
601	0	829	80.54
601	0	829	80.54
601	0	828	80.54
601	0	828	80.54
625	0	828	77.76
625	0	828	77.76
572	0	827	80.8
621	0	825	78.26
625	0	825	77.6
621	0	824	78.1
621	0	823	78.1
621	0	823	78.1
615	0	823	77.99
573	0	823	83.3
621	0	823	78.1
621	0	823	78.1
586	0	823	81.58
634	0	822	77.9
621	0	822	78.1
621	0	822	78.1
601	0	822	78.9
621	0	821	77.94
621	0	821	77.94
599	0	821	79.2

621	0	821	77.94
621	0	821	78.42
601	0	820	78.9
596	0	820	79.4
621	0	820	78.74
596	0	820	79.4
596	0	820	79.4
609	0	820	78.2
601	0	820	78.9
596	0	820	79.4
603	0	820	79.47
596	0	819	79.4
596	0	819	79.4
596	0	819	79.4
603	0	819	79.08
586	0	819	81.07
621	0	819	78.17
609	0	818	78.22
621	0	818	77.94
603	0	818	79.14
548	0	818	83.91
621	0	818	78.33
621	0	817	77.78
609	0	817	78.38
593	0	816	80.3
613	0	816	78.78
591	0	816	79.86
593	0	816	80.3
593	0	815	80.3
538	0	815	88.71
609	0	815	77.12
624	0	815	77.58
560	0	814	81.13
570	0	813	80.6
593	0	813	80.13
593	0	813	80.13
570	0	813	80.6
570	0	813	80.6
593	0	813	80.13
609	0	812	77.89
583	0	811	80.78
496	0	811	88.06
550	0	811	87.62
583	0	810	80.78
455	0	810	91.45

570	0	810	80.25
570	0	810	80.25
570	0	810	80.25
574	0	810	81.53
570	0	809	80.25
570	0	809	80.25
570	0	809	80.25
570	0	809	80.25
570	0	809	80.25
573	0	808	84.13
561	0	808	84.13
573	0	808	84.13
609	0	808	77.72
609	0	807	77.72
609	0	807	77.72

Supplementary Table 12. Homology to *rvh* genes from related species. While "*Ca.* A. rohweri" has annotations for all necessary components of the *rvh* T4SS, homology to other Rickettsiales varied between genes.

TrbI VirB10

Query acc.ve	Description	Subject acc.ver
WP_1260448	TrbI/VirB10 family protein [Candidatus Marinoinvertebrata rohwerii]	WP_126044823.1
WP_1260448	TrbI/VirB10 family protein [Candidatus Midichloria mitochondrii]	WP_013950964.1
WP_1260448	TrbI/VirB10 family protein [Rickettsiales endosymbiont of Peranema t	WP_130122150.1
WP_1260448	TrbI/VirB10 family protein [Candidatus Jidaibacter acanthamoeba]	WP_084212938.1
WP_1260448	Type IV secretion system protein VirB10 [Candidatus Jidaibacter acan	KIE04287.1
WP_1260448	type IV secretion system protein VirB10 [endosymbiont of Acanthamo	AIF81509.1
WP_1260448	TrbI/VirB10 family protein [endosymbiont of Acanthamoeba sp. UWC	WP_084173791.1
WP_1260448	hypothetical protein BGO27_04620 [Alphaproteobacteria bacterium 3	OJV13476.1
WP_1260448	TrbI/VirB10 family protein [Rickettsiales endosymbiont of Stachyamo	WP_125216831.1
WP_1260448	Type IV secretion system protein PtlG [Candidatus Arcanobacter lacus	KKB96703.1
WP_1260448	TrbI/VirB10 family protein [Sphingobacteriaceae bacterium]	RYE15760.1
WP_1260448	TrbI/VirB10 family protein [Alphaproteobacteria bacterium]	TAE80179.1
WP_1260448	TrbI/VirB10 family protein [Candidatus Fokinia solitaria]	WP_108673047.1
WP_1260448	TrbI/VirB10 family protein [Alphaproteobacteria bacterium]	TAE32155.1
WP_1260448	TrbI/VirB10 family protein [Rickettsiales bacterium Ac37b]	WP_038602661.1
WP_1260448	hypothetical protein COV36_00490 [Alphaproteobacteria bacterium C	PIR34606.1
WP_1260448	hypothetical protein A2048_08730 [Deltaproteobacteria bacterium GV	OGP07540.1
WP_1260448	TrbI/VirB10 family protein [Ehrlichia sp. HF]	WP_044195687.1
WP_1260448	hypothetical protein DI582_08085 [Azospirillum brasilense]	PZP84701.1
WP_1260448	bacterial conjugation TrbI-like family protein [Candidatus Neoehrlichia	KJV69360.1
WP_1260448	hypothetical protein [Candidatus Neoehrlichia lotoris]	WP_045809394.1
WP_1260448	TrbI/VirB10 family protein [Ehrlichia muris]	WP_024071660.1
WP_1260448	TrbI/VirB10 family protein [Anaplasma centrale]	WP_012880283.1
WP_1260448	TrbI/VirB10 family protein [Anaplasma ovis]	WP_075139319.1
WP_1260448	hypothetical protein A3D15_05170 [Alphaproteobacteria bacterium RI	OFW81273.1
WP_1260448	TrbI/VirB10 family protein [Anaplasma marginale]	WP_037330825.1
WP_1260448	MULTISPECIES: TrbI/VirB10 family protein [Anaplasma]	WP_010266574.1
WP_1260448	TrbI/VirB10 family protein [Anaplasma marginale]	WP_037348700.1
WP_1260448	VirB10 protein (virB10) [Anaplasma marginale str. Florida]	ACM49812.1
WP_1260448	VirB10 protein [Anaplasma marginale str. St. Maries]	AAV87106.1
WP_1260448	hypothetical protein [Ehrlichia minasensis]	WP_045171226.1
WP_1260448	TrbI/VirB10 family protein [Anaplasma marginale]	WP_041641675.1
WP_1260448	hypothetical protein U370_04935 [Anaplasma marginale str. Dawn]	AGZ80060.1
WP_1260448	Type IV secretion system protein virB10 [Anaplasma phagocytophilum	SCV65242.1
WP_1260448	TrbI/VirB10 family protein [Anaplasma phagocytophilum]	WP_021800101.1
WP_1260448	TrbI/VirB10 family protein [Rhizobium sp. 24NR]	WP_128445548.1
WP_1260448	TrbI/VirB10 family protein [Ehrlichia chaffeensis]	WP_006010239.1
WP_1260448	VirB10 [Ehrlichia chaffeensis]	AAM00413.1
WP_1260448	MULTISPECIES: TrbI/VirB10 family protein [Ehrlichia]	WP_045805002.1
WP_1260448	conjugal transfer protein TrbI [Ensifer sp. AP48]	WP_110880355.1

WP_1260448	type VI secretion protein [Neorickettsia sennetsu]	WP_011452123.1
WP_1260448	hypothetical protein [Ehrlichia ruminantium]	WP_065432268.1
WP_1260448	TrbI/VirB10 family protein [Anaplasma phagocytophilum]	WP_064670091.1
WP_1260448	TrbI/VirB10 family protein [Anaplasma phagocytophilum]	WP_064660097.1
WP_1260448	hypothetical protein [Ehrlichia ruminantium]	WP_065433546.1
WP_1260448	TrbI/VirB10 family protein [Anaplasma phagocytophilum]	WP_044152945.1
WP_1260448	TrbI/VirB10 family protein [Anaplasma phagocytophilum]	WP_011451402.1
WP_1260448	bacterial conjugation TrbI-like family protein [Anaplasma phagocytopl	KJV67435.1
WP_1260448	TrbI/VirB10 family protein [Anaplasma phagocytophilum]	WP_045890009.1
WP_1260448	TrbI/VirB10 family protein [Anaplasma phagocytophilum]	WP_044143786.1
WP_1260448	Type IV secretion system protein virB10 [Anaplasma phagocytophilum	SCV64305.1
WP_1260448	TrbI/VirB10 family protein [Anaplasma phagocytophilum]	WP_060757784.1
WP_1260448	hypothetical protein [Ehrlichia ruminantium]	WP_065433891.1
WP_1260448	conjugal transfer protein [Alphaproteobacteria bacterium]	PCI98723.1
WP_1260448	hypothetical protein [Ehrlichia ruminantium]	WP_011255231.1
WP_1260448	hypothetical protein X737_26180 [Mesorhizobium sp. L48C026A00]	ESZ12967.1
WP_1260448	TrbI/VirB10 family protein [Mesorhizobium sp. L48C026A00]	WP_084833833.1
WP_1260448	TrbI/VirB10 family protein [Ehrlichia canis]	WP_011304149.1
WP_1260448	hypothetical protein [Ehrlichia ruminantium]	WP_062850785.1
WP_1260448	TrbI/VirB10 family protein [Sinorhizobium fredii]	WP_037433938.1
WP_1260448	hypothetical protein [Ehrlichia ruminantium]	WP_011154711.1
WP_1260448	TrbI/VirB10 family protein [Rhizobiales bacterium]	WP_112947981.1
WP_1260448	TrbI/VirB10 family protein [Neorickettsia risticii]	WP_015816579.1
WP_1260448	TrbI/VirB10 family protein [Mesorhizobium sp.]	RWO94217.1
WP_1260448	TrbI/VirB10 family protein [Mesorhizobium amorphae]	WP_040583967.1
WP_1260448	conjugal transfer protein [Mesorhizobium amorphae CCNWGS0123]	EHH05196.1
WP_1260448	conjugal transfer protein TrbI [Rhizobiales bacterium]	WP_115097645.1
WP_1260448	TrbI/VirB10 family protein [Mesorhizobium sp. L2C066B000]	WP_023819760.1
WP_1260448	TrbI/VirB10 family protein [Mesorhizobium sp. Root172]	WP_056567614.1
WP_1260448	MULTISPECIES: TrbI/VirB10 family protein [Mesorhizobium]	WP_123170497.1
WP_1260448	TrbI/VirB10 family protein [Mesorhizobium waimense]	WP_120018525.1
WP_1260448	TrbI/VirB10 family protein [Mesorhizobium sp.]	RWC25261.1
WP_1260448	TrbI/VirB10 family protein [Rhizobium tubonense]	WP_111160813.1
WP_1260448	TrbI/VirB10 family protein [Mesorhizobium sp. M2A.F.Ca.ET.029.05.1	RVC91820.1
WP_1260448	conjugal transfer protein TrbI [Pseudochrobactrum sp. AO18b]	WP_022710645.1
WP_1260448	TrbI/VirB10 family protein [Mesorhizobium sp.]	RWF52477.1
WP_1260448	TrbI/VirB10 family protein [Mesorhizobium sp. M7A.F.Ca.ET.027.02.1	RVD08384.1
WP_1260448	type IV secretion protein VirB10 [Ochrobactrum sp. LM19]	AJW30008.1
WP_1260448	bacterial conjugation TrbI-like family protein [Neorickettsia helminthc	AHX11636.1
WP_1260448	TrbI/VirB10 family protein [Mesorhizobium sp. ICMP 18942]	WP_095204881.1
WP_1260448	TrbI/VirB10 family protein [Bosea sp. AS-1]	WP_089172751.1
WP_1260448	TrbI/VirB10 family protein [Mesorhizobium erdmanii]	WP_027054598.1
WP_1260448	TrbI/VirB10 family protein [Mesorhizobium sp.]	RWC83310.1
WP_1260448	conjugal transfer protein [Mesorhizobium japonicum MAFF 303099]	BAB54640.1
WP_1260448	TrbI/VirB10 family protein [Rhizobium sp. BK376]	WP_132527609.1

WP_1260448	hypothetical protein TW83_12570 [Paracoccus sp. S4493]	KJZ30786.1
WP_1260448	conjugal transfer protein TrbI [Rhizobiales bacterium]	WP_112155548.1
WP_1260448	TrbI/VirB10 family protein [Mesorhizobium japonicum]	WP_044552215.1
WP_1260448	TrbI/VirB10 family protein [Bosea sp. Tri-44]	WP_129160451.1
WP_1260448	TrbI/VirB10 family protein [Bosea vaviloviae]	WP_069694315.1
WP_1260448	conjugal transfer protein TrbI [Rhizobiales bacterium]	WP_112191667.1
WP_1260448	TrbI/VirB10 family protein [Agrobacterium sp. 13-2099-1-2]	WP_065703505.1
WP_1260448	conjugal transfer protein [Pseudomonas stutzeri]	PZR79492.1
WP_1260448	TrbI/VirB10 family protein [Rhizobiales bacterium]	WP_112695626.1
WP_1260448	TrbI/VirB10 family protein [Neorickettsia helminthoeca]	WP_038560672.1
WP_1260448	TrbI/VirB10 family protein [Bradyrhizobium sp. SK17]	WP_100957128.1
WP_1260448	conjugal transfer protein [Rhizobiales bacterium]	WP_113480298.1
WP_1260448	TrbI/VirB10 family protein [Agrobacterium tumefaciens]	WP_025591926.1
WP_1260448	TrbI/VirB10 family protein [Mesorhizobium sp.]	RWA81728.1
WP_1260448	MULTISPECIES: TrbI/VirB10 family protein [Bradyrhizobiaceae]	WP_044407314.1

% Identity	Alignment le	Mismatches	Gap opens	q. start	q. end	s. start
100	470	0	0	1	470	1
52.721	294	128	4	167	457	196
50.345	290	134	3	171	459	11
48.621	290	140	3	169	457	167
48.621	290	140	3	169	457	171
47.931	290	142	3	169	457	165
47.931	290	142	3	169	457	166
32.11	436	233	10	35	457	27
45.299	234	114	4	238	457	186
46.729	214	109	3	245	457	211
46.602	206	99	4	256	457	2
40.824	267	147	5	191	456	168
33.989	356	216	5	109	456	79
40.449	267	148	5	191	456	168
41.155	277	140	6	182	457	152
38.356	292	164	5	169	457	121
37.591	274	135	6	183	456	98
33.945	436	229	13	45	457	26
43.779	217	115	3	241	457	80
33.721	430	224	13	45	457	27
33.721	430	224	13	45	457	26
31.057	454	247	11	37	457	16
44.783	230	119	4	229	457	217
41.328	271	134	8	190	457	187
43.056	216	118	1	242	457	228
44.348	230	115	5	231	457	216
44.348	230	115	5	231	457	216
44.348	230	115	5	231	457	216
44.348	230	115	5	231	457	220
44.348	230	115	5	231	457	220
31.857	474	249	14	16	457	4
44.348	230	115	5	231	457	216
44.348	230	115	5	231	457	220
29.279	444	254	9	38	457	21
30	450	242	12	38	457	21
41.071	224	127	2	238	456	213
31.866	477	246	13	16	457	4
31.866	477	246	13	16	457	4
30.737	475	216	15	37	457	16
31.94	335	188	9	127	456	80

28.543	494	275	13	13	468	4
30.425	447	259	13	37	457	17
28.894	443	256	8	38	457	21
28.668	443	257	8	38	457	21
30.425	447	259	13	37	457	17
29.333	450	247	11	38	457	21
29.556	450	246	12	38	457	21
40.455	220	127	3	239	457	192
40.455	220	127	3	239	457	212
40.455	220	127	3	239	457	212
40.455	220	127	3	239	457	212
40	220	128	3	239	457	212
30.022	453	259	13	37	457	17
40.187	214	103	3	243	456	196
30.787	445	258	13	37	457	17
41.228	228	120	5	240	456	189
41.228	228	120	5	240	456	210
42.797	236	110	5	239	457	207
30.425	447	259	13	37	457	17
31.875	320	182	8	142	456	91
30.425	447	259	13	37	457	17
29.455	404	227	10	53	456	34
36.626	243	121	5	244	468	186
41.589	214	114	3	253	456	208
38.053	226	125	3	241	456	205
38.053	226	125	3	241	456	197
33.531	337	183	11	123	456	92
38.053	226	125	3	241	456	205
38.053	226	125	3	241	456	205
38.053	226	125	3	241	456	205
38.053	226	125	3	241	456	198
37.611	226	126	3	241	456	205
35.455	220	134	1	245	456	192
37.611	226	126	3	241	456	98
37.915	211	114	3	246	456	194
37.611	226	126	3	241	456	96
37.118	229	129	3	238	456	202
28.986	414	237	8	46	456	30
29.495	495	266	15	16	467	6
38.053	226	125	3	241	456	202
35.586	222	132	3	245	456	200
38.053	226	125	3	241	456	205
37.611	226	126	3	241	456	205
37.168	226	127	3	241	456	163
30.495	364	187	9	116	456	75

40.465	215	100	5	245	454	212
40.278	216	109	6	243	456	195
37.168	226	127	3	241	456	205
35.193	233	118	3	245	456	200
37.22	223	131	3	243	456	202
40.278	216	109	6	243	456	195
39.352	216	111	5	243	456	196
36.323	223	129	3	245	456	155
29.469	414	242	8	44	456	25
36.015	261	127	7	231	467	172
36.771	223	127	4	245	456	201
33.884	242	149	2	226	456	179
39.815	216	110	6	243	456	195
37.383	214	118	1	243	456	185
36.404	228	133	3	241	456	194

s. end	evalue	Bit score	% Positives
470	0	939	100
481	5.07E-87	284	71.77
291	2.46E-81	263	67.24
448	9.50E-81	267	67.93
452	1.37E-80	267	67.93
446	6.01E-79	262	68.28
447	6.73E-79	262	68.28
412	7.77E-52	190	50.69
419	2.19E-51	189	61.11
420	1.73E-50	187	69.63
200	2.77E-50	180	67.48
424	3.44E-50	186	60.3
423	5.81E-50	186	55.9
424	1.18E-49	185	60.3
406	2.51E-49	184	57.4
399	4.87E-49	182	53.42
335	6.63E-49	181	56.93
425	1.99E-48	182	49.54
289	6.36E-48	176	61.75
412	4.81E-46	175	51.4
411	5.73E-46	175	51.4
436	1.13E-43	169	48.02
439	1.43E-43	169	61.3
435	4.09E-43	167	57.93
438	8.13E-43	167	61.11
435	9.21E-43	166	61.3
435	9.50E-43	166	61.3
435	1.01E-42	166	61.3
439	1.14E-42	166	61.3
439	1.29E-42	166	61.3
435	2.64E-42	166	47.05
435	2.94E-42	165	60.87
439	3.63E-42	165	60.87
428	2.55E-41	162	47.97
427	2.92E-41	162	49.11
436	2.99E-41	163	57.59
436	3.42E-41	162	45.7
436	3.86E-41	162	45.7
431	1.71E-40	160	46.32
379	8.59E-40	157	51.64

457	1.19E-39	158	45.95
437	2.36E-39	157	48.1
427	2.91E-39	157	47.4
427	3.39E-39	157	47.86
437	3.47E-39	157	48.1
429	4.02E-39	156	48
429	4.83E-39	156	48.22
408	7.67E-39	155	61.36
428	1.37E-38	155	60.91
428	1.38E-38	155	60.91
428	1.41E-38	155	60.91
428	1.71E-38	155	60.91
443	1.85E-38	155	47.46
384	3.16E-38	153	61.21
435	3.39E-38	154	48.31
413	6.51E-38	154	56.58
434	7.70E-38	154	56.58
434	1.89E-37	152	55.93
437	1.99E-37	152	47.87
379	2.31E-37	151	51.88
437	2.46E-37	152	47.87
379	2.70E-37	151	47.52
413	2.89E-37	151	56.79
420	6.23E-37	151	57.01
425	6.70E-37	151	58.41
417	6.99E-37	150	58.41
390	8.12E-37	150	50.74
425	9.07E-37	150	58.41
425	9.63E-37	150	58.41
425	1.23E-36	150	58.41
418	1.43E-36	150	57.08
425	2.12E-36	149	57.96
411	2.27E-36	149	56.36
318	2.29E-36	146	57.96
387	2.34E-36	148	59.72
316	2.48E-36	147	57.96
425	2.53E-36	149	58.08
389	3.31E-36	148	45.65
460	3.57E-36	149	46.06
422	4.12E-36	149	57.08
420	8.63E-36	148	60.81
425	1.02E-35	147	57.08
425	1.03E-35	147	57.96
383	1.08E-35	147	57.96
395	1.30E-35	147	48.35

403	1.44E-35	146	60
392	1.55E-35	146	58.33
425	2.31E-35	147	57.96
420	2.69E-35	146	56.22
424	2.79E-35	146	59.19
392	2.81E-35	145	58.33
393	2.83E-35	145	58.33
375	3.47E-35	145	60.09
389	6.22E-35	144	46.14
416	6.53E-35	145	55.17
420	6.65E-35	145	60.54
420	7.07E-35	145	56.61
392	7.72E-35	144	58.33
382	1.00E-34	144	55.14
421	1.33E-34	144	58.77

Supplementary Table 12. Homology to *rvh* genes from related species. While "*Ca.* A. rohweri" has annotations for all necessary components of the *rvh* T4SS, homology to other Rickettsiales varied between genes.

VirB11

Query acc.ve	Description	Subject acc.ver
WP_1260448	P-type DNA transfer ATPase VirB11 [Candidatus Marinoinvertebrata r	WP_126044822.1
WP_1260448	Type IV secretion system protein VirB11 [Candidatus Jidaibacter acan	KIE04286.1
WP_1260448	P-type DNA transfer ATPase VirB11 [Candidatus Jidaibacter acanthan	WP_039458872.1
WP_1260448	P-type DNA transfer ATPase VirB11 [endosymbiont of Acanthamoeba	WP_038539497.1
WP_1260448	P-type DNA transfer ATPase VirB11 [Candidatus Midichloria mitochor	WP_013950963.1
WP_1260448	P-type DNA transfer ATPase VirB11 [Rickettsiales endosymbiont of Pe	WP_130122149.1
WP_1260448	P-type DNA transfer ATPase VirB11 [Rickettsia endosymbiont of Culic	WP_094648715.1
WP_1260448	MULTISPECIES: P-type DNA transfer ATPase VirB11 [Rickettsia]	WP_012151688.1
WP_1260448	P-type DNA transfer ATPase VirB11 [Rickettsia bellii]	WP_011477676.1
WP_1260448	P-type DNA transfer ATPase VirB11 [Rickettsia typhi]	WP_011190748.1
WP_1260448	Type IV secretion system protein VirB11 [Candidatus Arcanobacter la	KKB96704.1
WP_1260448	P-type DNA transfer ATPase VirB11 [Rickettsia bellii]	WP_045798975.1
WP_1260448	P-type DNA transfer ATPase VirB11 [Rickettsia bellii]	WP_045799528.1
WP_1260448	P-type DNA transfer ATPase VirB11 [Rickettsia felis]	WP_011270800.1
WP_1260448	P-type DNA transfer ATPase VirB11 [Rickettsia tamurae]	WP_032139524.1
WP_1260448	P-type DNA transfer ATPase VirB11 [Rickettsia asembonensis]	WP_041079409.1
WP_1260448	P-type DNA transfer ATPase VirB11 [Rickettsia hoogstraalii]	WP_040257360.1
WP_1260448	P-type DNA transfer ATPase VirB11 [Rickettsia felis]	WP_045820724.1
WP_1260448	P-type DNA transfer ATPase VirB11 [Rickettsia sp. wb]	WP_068945257.1
WP_1260448	P-type DNA transfer ATPase VirB11 [Orientia chuto]	WP_045797532.1
WP_1260448	P-type DNA transfer ATPase VirB11 [Rickettsia prowazekii]	WP_004597371.1
WP_1260448	P-type DNA transfer ATPase VirB11 [Rickettsia endosymbiont of Ixode	WP_045804050.1
WP_1260448	P-type DNA transfer ATPase VirB11 [Rickettsia monacensis]	WP_023508008.1
WP_1260448	MULTISPECIES: P-type DNA transfer ATPase VirB11 [spotted fever gro	WP_037214280.1
WP_1260448	P-type DNA transfer ATPase VirB11 [Rickettsia hoogstraalii str. RCCE3	KJV81194.1
WP_1260448	P-type DNA transfer ATPase VirB11 [Rickettsia sibirica]	WP_004996187.1
WP_1260448	P-type DNA transfer ATPase VirB11 [Rickettsia sibirica]	WP_016728114.1
WP_1260448	P-type DNA transfer ATPase VirB11 [Rickettsia montanensis]	WP_014410092.1
WP_1260448	P-type DNA transfer ATPase VirB11 [Ehrlichia canis]	WP_011304148.1
WP_1260448	P-type DNA transfer ATPase VirB11 [Rickettsia africae]	WP_012719547.1
WP_1260448	P-type DNA transfer ATPase VirB11 [Rickettsia helvetica]	WP_010423477.1
WP_1260448	P-type DNA transfer ATPase VirB11 [Orientia tsutsugamushi]	WP_045918245.1
WP_1260448	P-type DNA transfer ATPase VirB11 [Orientia tsutsugamushi]	WP_011944233.1
WP_1260448	P-type DNA transfer ATPase VirB11 [Rickettsia heilongjiangensis]	WP_014014093.1
WP_1260448	MULTISPECIES: P-type DNA transfer ATPase VirB11 [spotted fever gro	WP_014120655.1
WP_1260448	P-type DNA transfer ATPase VirB11 [Rickettsia aeschlimannii]	WP_032074043.1
WP_1260448	P-type DNA transfer ATPase VirB11 [Orientia tsutsugamushi]	WP_047220077.1
WP_1260448	P-type DNA transfer ATPase VirB11 [Ehrlichia minasensis]	WP_045171225.1
WP_1260448	P-type DNA transfer ATPase VirB11 [Orientia tsutsugamushi]	WP_109234422.1
WP_1260448	P-type DNA transfer ATPase VirB11 [Rickettsia parkeri]	WP_014410529.1

WP_1260448	P-type DNA transfer ATPase VirB11 [Rickettsia amblyommatis]	WP_014392038.1
WP_1260448	P-type DNA transfer ATPase VirB11 [Rickettsia raoultii]	WP_064463394.1
WP_1260448	P-type DNA transfer ATPase VirB11 [Rickettsia peacockii]	WP_012736766.1
WP_1260448	P-type DNA transfer ATPase VirB11 [Orientia tsutsugamushi]	WP_045912171.1
WP_1260448	P-type DNA transfer ATPase VirB11 [Rickettsia honei]	WP_016916606.1
WP_1260448	MULTISPECIES: P-type DNA transfer ATPase VirB11 [spotted fever gro	WP_012150601.1
WP_1260448	P-type DNA transfer ATPase VirB11 [Rickettsia fournieri]	WP_103897249.1
WP_1260448	P-type DNA transfer ATPase VirB11 [Rickettsia australis]	WP_014412973.1
WP_1260448	P-type DNA transfer ATPase VirB11 [Rickettsia slovaca]	WP_014273332.1
WP_1260448	P-type DNA transfer ATPase VirB11 [Rickettsia conorii]	WP_016926113.1
WP_1260448	P-type DNA transfer ATPase VirB11 [Rickettsia canadensis]	WP_012148953.1
WP_1260448	P-type DNA transfer ATPase VirB11 [Rickettsia conorii]	WP_010977044.1
WP_1260448	P-type DNA transfer ATPase VirB11 [Rickettsia sp. Tenjiku01]	WP_064429003.1
WP_1260448	P-type DNA transfer ATPase VirB11 [Rickettsia endosymbiont of Proe	WP_062811334.1
WP_1260448	P-type DNA transfer ATPase VirB11 [Rickettsia canadensis]	WP_014364095.1
WP_1260448	P-type DNA transfer ATPase VirB11 [Rickettsia rhipicephali]	WP_014408410.1
WP_1260448	P-type DNA transfer ATPase VirB11 [Rickettsiales bacterium Ac37b]	WP_038602658.1
WP_1260448	MULTISPECIES: P-type DNA transfer ATPase VirB11 [spotted fever gro	WP_014366022.1
WP_1260448	P-type DNA transfer ATPase VirB11 [Ehrlichia muris]	WP_024071659.1
WP_1260448	P-type DNA transfer ATPase VirB11 [Rickettsiaceae bacterium]	RYE05972.1
WP_1260448	P-type DNA transfer ATPase VirB11 [Rickettsia massiliae]	WP_041404587.1
WP_1260448	VirB11 [Rickettsia massiliae MTU5]	ABV84643.1
WP_1260448	P-type DNA transfer ATPase VirB11 [Rickettsia akari]	WP_012149359.1
WP_1260448	MULTISPECIES: P-type DNA transfer ATPase VirB11 [Ehrlichia]	WP_045805003.1
WP_1260448	P-type DNA transfer ATPase VirB11 [Ehrlichia ruminantium]	WP_011154710.1
WP_1260448	P-type DNA transfer ATPase VirB11 [Rickettsiales endosymbiont of St	WP_125216832.1
WP_1260448	P-type DNA transfer ATPase VirB11 [Ehrlichia ruminantium]	WP_065433518.1
WP_1260448	P-type DNA transfer ATPase VirB11 [Ehrlichia sp. HF]	WP_044195690.1
WP_1260448	P-type DNA transfer ATPase VirB11 [Ehrlichia ruminantium]	WP_065432267.1
WP_1260448	P-type DNA transfer ATPase VirB11 [Rickettsia gravesii]	WP_024547252.1
WP_1260448	VirB11 [Rickettsia amblyommatis]	ABK15689.2
WP_1260448	P-type DNA transfer ATPase VirB11 [Alphaproteobacteria bacterium 3	OJV13475.1
WP_1260448	P-type DNA transfer ATPase VirB11 [Ehrlichia chaffeensis]	WP_006010241.1
WP_1260448	P-type DNA transfer ATPase VirB11 [Wolbachia endosymbiont of Ben	WP_108784159.1
WP_1260448	P-type DNA transfer ATPase VirB11 [Wolbachia endosymbiont of Tric	WP_068651312.1
WP_1260448	VirB11 [Ehrlichia chaffeensis]	AAM00414.1
WP_1260448	P-type DNA transfer ATPase VirB11 [Wolbachia endosymbiont of Cim	WP_041044922.1
WP_1260448	VirB11 [Wolbachia endosymbiont of Porcellio variabilis]	ACO52142.1
WP_1260448	P-type DNA transfer ATPase VirB11 [Wolbachia endosymbiont of Fols	WP_110409418.1
WP_1260448	P-type DNA transfer ATPase VirB11 [Wolbachia endosymbiont of Nas	WP_010404943.1
WP_1260448	MULTISPECIES: P-type DNA transfer ATPase VirB11 [Wolbachia]	WP_007302612.1
WP_1260448	P-type DNA transfer ATPase VirB11 [Occidentia massiliensis]	WP_019230978.1
WP_1260448	VirB11 [Wolbachia endosymbiont of Armadillidium vulgare]	AAX86708.1
WP_1260448	VirB11 [Wolbachia endosymbiont of Segestria florentina]	ACO52147.1
WP_1260448	VirB11 [Wolbachia endosymbiont of Ligia oceanica]	ACO52136.1

WP_1260448	MULTISPECIES: P-type DNA transfer ATPase VirB11 [Wolbachia]	WP_019236952.1
WP_1260448	type IV secretion system component VirB11 [Wolbachia endosymbion	AIF71180.1
WP_1260448	VirB11 [Wolbachia endosymbiont of Porcellio dilatatus petiti]	ACO52139.1
WP_1260448	P-type DNA transfer ATPase VirB11 [Wolbachia pipientis]	WP_006012542.1
WP_1260448	VirB11 [Wolbachia endosymbiont of Porcellionides pruinosus]	ACO52143.1
WP_1260448	VirB11 [Wolbachia endosymbiont of Porcellio dispar]	ACO52140.1
WP_1260448	P-type DNA transfer ATPase VirB11 [Alphaproteobacteria bacterium I	OFW81362.1
WP_1260448	P-type DNA transfer ATPase VirB11 [Wolbachia endosymbiont of Cyli	WP_114517614.1
WP_1260448	VirB11 [Wolbachia endosymbiont of Dysdera erythrina]	ACO52146.1
WP_1260448	P-type DNA transfer ATPase VirB11 [Wolbachia endosymbiont of Dros	WP_015588553.1
WP_1260448	component of type IV secretion system [Wolbachia sp. wTai]	BAA97435.1
WP_1260448	VirB11 [Wolbachia endosymbiont of Armadillidium album]	ACO52128.1
WP_1260448	MULTISPECIES: P-type DNA transfer ATPase VirB11 [Wolbachia]	WP_065094644.1
WP_1260448	MULTISPECIES: P-type DNA transfer ATPase VirB11 [Wolbachia]	WP_010962302.1
WP_1260448	P-type DNA transfer ATPase VirB11 [Alphaproteobacteria bacterium I	OFX11536.1

% Identity	Alignment le	Mismatches	Gap opens	q. start	q. end	s. start	
100	327	0	0	1	327	1	
76.758	327	76	0	1	327	1	
76.923	325	75	0	3	327	1	
76.615	325	76	0	3	327	1	
77.399	323	73	0	3	325	1	
76.78	323	75	0	3	325	1	
73.148	324	87	0	1	324	1	
73.231	325	87	0	1	325	1	
72.923	325	88	0	1	325	1	
72.308	325	90	0	1	325	1	
73.913	322	84	0	4	325	3	
72.923	325	88	0	1	325	1	
72.615	325	89	0	1	325	1	
72.308	325	90	0	1	325	1	
72.308	325	90	0	1	325	1	
72.308	325	90	0	1	325	1	
72.308	325	90	0	1	325	1	
72	325	91	0	1	325	1	
73.457	324	86	0	1	324	1	
71.429	322	92	0	4	325	3	
71.385	325	93	0	1	325	1	
72	325	91	0	1	325	1	
72	325	91	0	1	325	1	
72	325	91	0	1	325	1	
72	325	91	0	1	325	1	
72	325	91	0	1	325	1	
72	325	91	0	1	325	1	
71.692	325	92	0	1	325	1	
71.254	327	94	0	1	327	1	
71.692	325	92	0	1	325	1	
72	325	91	0	1	325	1	
72.36	322	89	0	4	325	3	
72.36	322	89	0	4	325	3	
71.385	325	93	0	1	325	1	
71.385	325	93	0	1	325	1	
71.692	325	92	0	1	325	1	
72.36	322	89	0	4	325	3	
71.254	327	94	0	1	327	1	
72.36	322	89	0	4	325	3	
71.692	325	92	0	1	325	1	

71.385	325	93	0	1	325	1
71.385	325	93	0	1	325	1
71.385	325	93	0	1	325	1
72.05	322	90	0	4	325	3
71.385	325	93	0	1	325	1
71.385	325	93	0	1	325	1
71.077	325	94	0	1	325	1
71.692	325	92	0	1	325	1
71.385	325	93	0	1	325	1
71.385	325	93	0	1	325	1
71.692	325	92	0	1	325	1
71.385	325	93	0	1	325	1
71.385	325	93	0	1	325	1
71.385	325	93	0	1	325	1
71.692	325	92	0	1	325	1
71.077	325	94	0	1	325	1
72.05	322	90	0	4	325	3
70.769	325	95	0	1	325	1
70.031	327	98	0	1	327	1
71.207	323	93	0	3	325	1
70.462	325	96	0	1	325	1
70.462	325	96	0	1	325	14
70.462	325	96	0	1	325	1
69.419	327	100	0	1	327	1
69.725	327	99	0	1	327	1
72.36	322	89	0	4	325	3
69.419	327	100	0	1	327	1
69.725	327	99	0	1	327	1
69.419	327	100	0	1	327	1
73.016	315	85	0	1	315	1
71.924	317	89	0	1	317	1
71.429	322	92	0	4	325	3
69.725	327	99	0	1	327	1
70.769	325	95	0	3	327	1
70.154	325	97	0	3	327	1
69.419	327	100	0	1	327	1
69.846	325	98	0	3	327	1
70.769	325	95	0	3	327	1
70.154	325	97	0	3	327	1
70.462	325	96	0	3	327	1
70.462	325	96	0	3	327	1
70.588	323	95	0	3	325	1
70.497	322	95	0	3	324	1
70.462	325	96	0	3	327	1
69.846	325	98	0	3	327	1

70.122	328	92	2	3	327	1
70.154	325	97	0	3	327	1
69.846	325	98	0	3	327	1
70.154	325	97	0	3	327	1
70.122	328	92	2	3	327	1
69.846	325	98	0	3	327	1
70.186	322	96	0	4	325	3
70.462	325	96	0	3	327	1
69.846	325	98	0	3	327	1
70.154	325	97	0	3	327	1
69.846	325	98	0	3	327	1
70.186	322	96	0	3	324	1
69.538	325	99	0	3	327	1
69.538	325	99	0	3	327	1
70.186	322	96	0	4	325	3

s. end	evalue	Bit score	% Positives
327	0	670	100
327	0	530	88.99
325	0	528	89.23
325	0	527	89.23
323	0	519	87
323	0	518	88.85
324	4.04E-175	497	84.88
325	9.42E-175	496	84
325	1.52E-174	496	84
325	2.05E-174	496	84.62
324	3.69E-174	495	84.16
325	5.20E-174	494	83.69
325	2.46E-173	493	83.69
325	2.57E-173	493	84
325	2.57E-173	493	84
325	2.81E-173	493	84
325	3.03E-173	493	84
325	5.13E-173	492	84
324	5.98E-173	491	83.33
324	6.27E-173	492	86.02
325	1.48E-172	491	84.31
325	1.58E-172	491	83.69
325	1.58E-172	491	83.69
325	1.60E-172	491	83.69
325	1.77E-172	491	84
325	1.81E-172	491	83.69
325	1.81E-172	491	83.69
325	2.11E-172	491	84
327	2.26E-172	490	83.49
325	2.32E-172	490	83.69
325	2.89E-172	490	83.69
324	2.91E-172	490	84.47
324	3.32E-172	490	84.47
325	4.06E-172	490	83.69
325	4.53E-172	489	83.69
325	4.84E-172	489	83.69
324	4.85E-172	489	84.16
327	4.97E-172	489	83.49
324	6.20E-172	489	84.16
325	6.65E-172	489	83.69

325	1.11E-171	489	83.69
325	1.19E-171	489	83.69
325	1.72E-171	488	83.69
324	1.77E-171	488	84.16
325	1.90E-171	488	83.69
325	2.03E-171	488	83.69
325	2.12E-171	488	83.69
325	2.17E-171	488	83.69
325	2.19E-171	488	83.69
325	2.92E-171	488	83.69
325	3.05E-171	488	83.08
325	3.48E-171	488	83.69
325	3.87E-171	488	83.69
325	4.72E-171	487	83.69
325	5.63E-171	487	83.08
325	6.01E-171	487	83.38
324	6.32E-171	487	83.85
325	1.10E-170	486	83.38
327	1.91E-170	486	83.18
323	3.25E-170	485	83.9
325	1.35E-169	483	83.08
338	2.98E-169	483	83.08
325	5.18E-169	482	82.77
327	1.03E-168	481	82.87
327	1.76E-168	481	81.65
324	1.99E-168	480	83.23
327	2.41E-168	480	81.96
327	2.94E-168	480	82.87
327	4.12E-168	479	81.65
315	1.19E-166	475	83.49
317	1.89E-166	475	83.28
324	3.09E-166	475	81.99
327	5.25E-165	472	83.79
325	7.92E-165	471	82.15
325	8.74E-165	471	82.15
327	1.75E-164	470	83.49
325	1.88E-164	470	82.15
325	1.99E-164	470	81.23
325	2.07E-164	470	82.15
325	3.21E-164	469	82.15
325	7.23E-164	469	81.85
323	8.83E-164	469	80.8
322	9.40E-164	468	81.99
325	9.61E-164	469	81.85
325	1.17E-163	468	82.46

325	2.44E-163	468	82.32
325	2.93E-163	467	81.85
325	2.93E-163	467	82.15
325	3.75E-163	467	81.85
325	3.94E-163	467	82.32
325	4.03E-163	467	82.15
324	5.56E-163	467	82.61
325	5.85E-163	466	80.92
325	7.12E-163	466	81.54
325	1.03E-162	466	81.85
325	1.08E-162	466	81.85
322	1.15E-162	466	82.3
325	1.20E-162	466	81.54
325	1.44E-162	466	81.54
324	1.47E-162	466	82.61

Supplementary Table 12. Homology to *rvh* genes from related species. While "*Ca.* A. rohweri" has annotations for all necessary components of the *rvh* T4SS, homology to other Rickettsiales varied between genes.

VirB9

Query acc.ve	Description	Subject acc.ver
WP_1260445	P-type conjugative transfer protein VirB9 [Candidatus Marinoinverteb	WP_126044579.1
WP_1260445	P-type conjugative transfer protein VirB9 [endosymbiont of Acantham	WP_038539680.1
WP_1260445	P-type conjugative transfer protein VirB9 [Candidatus Jidaibacter acar	WP_039455241.1
WP_1260445	putative type IV secretion system protein [Candidatus Jidaibacter acai	KIE05965.1
WP_1260445	P-type conjugative transfer protein VirB9 [Rickettsiales endosymbiont	WP_130122312.1
WP_1260445	P-type conjugative transfer protein VirB9 [Candidatus Midichloria mit	WP_013951379.1
WP_1260445	P-type conjugative transfer protein VirB9 [Candidatus Fokinia solitaria	WP_108673052.1
WP_1260445	P-type conjugative transfer protein VirB9 [Rickettsiales bacterium Ac3	WP_044578096.1
WP_1260445	P-type conjugative transfer protein VirB9 [Rickettsiales endosymbiont	WP_125216029.1
WP_1260445	P-type conjugative transfer protein VirB9 [Azospirillum brasilense]	PZP84455.1
WP_1260445	P-type conjugative transfer protein VirB9 [Alphaproteobacteria bacter	PIR31945.1
WP_1260445	P-type conjugative transfer protein VirB9 [Occidentia massiliensis]	WP_019231305.1
WP_1260445	P-type conjugative transfer protein VirB9 [Rickettsiaceae bacterium]	RYE05781.1
WP_1260445	Type IV secretion system protein VirB9 [Candidatus Phycorickettsia tra	AVP87324.1
WP_1260445	P-type conjugative transfer protein VirB9 [Candidatus Phycorickettsia	WP_106874192.1
WP_1260445	P-type conjugative transfer protein VirB9 [Alphaproteobacteria bacter	PIR38655.1
WP_1260445	P-type conjugative transfer protein VirB9 [Rickettsia endosymbiont of	WP_094649371.1
WP_1260445	P-type conjugative transfer protein VirB9 [Alphaproteobacteria bacter	OJV15832.1
WP_1260445	P-type conjugative transfer protein VirB9 [Rhodospirillales bacterium	OYW13908.1
WP_1260445	P-type conjugative transfer protein VirB9 [Rickettsia raoultii]	WP_064463384.1
WP_1260445	P-type conjugative transfer protein VirB9 [Rickettsia monacensis]	WP_023508002.1
WP_1260445	P-type conjugative transfer protein VirB9 [Rickettsia parkeri]	WP_014410525.1
WP_1260445	P-type conjugative transfer protein VirB9 [Anaplasma ovis]	WP_075138652.1
WP_1260445	P-type conjugative transfer protein VirB9 [Rickettsia aeschlimannii]	WP_032074039.1
WP_1260445	conjugal transfer protein [Anaplasma marginale]	AGC65497.1
WP_1260445	P-type conjugative transfer protein VirB9 [Rickettsia philipii]	WP_014364553.1
WP_1260445	MULTISPECIES: P-type conjugative transfer protein VirB9 [spotted fev	WP_016770014.1
WP_1260445	P-type conjugative transfer protein VirB9 [Rickettsia akari]	WP_012149353.1
WP_1260445	MULTISPECIES: P-type conjugative transfer protein VirB9 [Rickettsia]	WP_008580257.1
WP_1260445	P-type conjugative transfer protein VirB9 [Rickettsia rickettsii]	WP_012150596.1
WP_1260445	P-type conjugative transfer protein VirB9 [Rickettsia sibirica]	WP_004996199.1
WP_1260445	MULTISPECIES: P-type conjugative transfer protein VirB9 [spotted fev	WP_010977039.1
WP_1260445	P-type conjugative transfer protein VirB9 [Rickettsia australis]	WP_014412979.1
WP_1260445	P-type conjugative transfer protein VirB9 [Rickettsia rickettsii]	WP_014363083.1
WP_1260445	P-type conjugative transfer protein VirB9 [Rickettsia sp. Tenjiku01]	WP_064428998.1
WP_1260445	P-type conjugative transfer protein VirB9 [Rickettsia argasii]	WP_045805409.1
WP_1260445	P-type conjugative transfer protein VirB9 [Rickettsia japonica]	WP_014120650.1
WP_1260445	P-type conjugative transfer protein VirB9 [Rickettsia heilongjiangensis	WP_014014087.1
WP_1260445	P-type conjugative transfer protein VirB9 [Rickettsia asembonensis]	WP_041079420.1
WP_1260445	P-type conjugative transfer protein VirB9 [Rickettsia helvetica]	WP_010423483.1

WP_1260445	P-type conjugative transfer protein VirB9 [Rickettsia gravesii]	WP_017443173.1
WP_1260445	P-type conjugative transfer protein VirB9 [Rickettsia amblyommatis]	WP_045800243.1
WP_1260445	P-type conjugative transfer protein VirB9 [Rickettsia canadensis]	WP_014364098.1
WP_1260445	P-type conjugative transfer protein VirB9 [Rickettsia massiliae]	WP_041404585.1
WP_1260445	VirB9 [Rickettsia massiliae MTU5]	ABV84637.1
WP_1260445	P-type conjugative transfer protein VirB9 [Rickettsia endosymbiont of	WP_062811328.1
WP_1260445	P-type conjugative transfer protein VirB9 [Anaplasma marginale]	WP_011114110.1
WP_1260445	P-type conjugative transfer protein VirB9 [Rickettsia montanensis]	WP_014410087.1
WP_1260445	MULTISPECIES: P-type conjugative transfer protein VirB9 [Anaplasma	WP_010266984.1
WP_1260445	P-type conjugative transfer protein VirB9 [Rickettsia prowazekii]	WP_004597362.1
WP_1260445	P-type conjugative transfer protein VirB9 [Rickettsia honei]	WP_016916601.1
WP_1260445	P-type conjugative transfer protein VirB9 [Rickettsia typhi]	WP_011190742.1
WP_1260445	P-type conjugative transfer protein VirB9 [Anaplasma marginale]	WP_114211742.1
WP_1260445	P-type conjugative transfer protein VirB9 [Rickettsiales bacterium]	PCJ29633.1
WP_1260445	P-type conjugative transfer protein VirB9 [Rickettsia peacockii]	WP_012736761.1
WP_1260445	P-type conjugative transfer protein VirB9 [Rickettsia canadensis]	WP_012148958.1
WP_1260445	P-type conjugative transfer protein VirB9 [Rickettsia bellii]	WP_011477671.1
WP_1260445	P-type conjugative transfer protein VirB9 [Rickettsia bellii]	WP_041808388.1
WP_1260445	P-type conjugative transfer protein VirB9 [Rickettsiales bacterium]	RTK92103.1
WP_1260445	P-type conjugative transfer protein VirB9 [Anaplasma centrale]	WP_012881051.1
WP_1260445	MULTISPECIES: P-type conjugative transfer protein VirB9 [unclassified	WP_016948045.1
WP_1260445	P-type conjugative transfer protein VirB9 [Wolbachia endosymbiont o	WP_108783925.1
WP_1260445	Type IV secretion system protein PtlF precursor [Wolbachia endosymb	OJH31119.1
WP_1260445	MULTISPECIES: P-type conjugative transfer protein VirB9 [Wolbachia]	WP_006014058.1
WP_1260445	P-type conjugative transfer protein VirB9 [Alphaproteobacteria bacter	TAE82972.1
WP_1260445	P-type conjugative transfer protein VirB9 [Wolbachia endosymbiont o	WP_114517929.1
WP_1260445	P-type conjugative transfer protein VirB9 [Alphaproteobacteria bacter	TAE33698.1
WP_1260445	MULTISPECIES: P-type conjugative transfer protein VirB9 [Wolbachia]	WP_007302273.1
WP_1260445	P-type conjugative transfer protein VirB9 [Anaplasma phagocytophilu	WP_064659608.1
WP_1260445	MULTISPECIES: P-type conjugative transfer protein VirB9 [Wolbachia]	WP_064125428.1
WP_1260445	MULTISPECIES: P-type conjugative transfer protein VirB9 [Wolbachia]	WP_006280053.1
WP_1260445	P-type conjugative transfer protein VirB9 [Wolbachia pipientis]	WP_096641481.1
WP_1260445	hypothetical protein A3D15_05215 [Alphaproteobacteria bacterium R	OFW80548.1
WP_1260445	P-type conjugative transfer protein VirB9 [Wolbachia endosymbiont o	WP_110409834.1
WP_1260445	P-type conjugative transfer protein VirB9 [Anaplasma phagocytophilu	WP_045890059.1
WP_1260445	P-type conjugative transfer protein VirB9 [Wolbachia endosymbiont o	WP_015588119.1
WP_1260445	MULTISPECIES: P-type conjugative transfer protein VirB9 [Wolbachia]	WP_010082330.1
WP_1260445	MULTISPECIES: P-type conjugative transfer protein VirB9 [Wolbachia]	WP_006280123.1
WP_1260445	P-type conjugative transfer protein VirB9 [Wolbachia endosymbiont o	WP_044471313.1
WP_1260445	P-type conjugative transfer protein VirB9 [Anaplasma phagocytophilu	WP_011450238.1
WP_1260445	P-type conjugative transfer protein VirB9 [Anaplasma phagocytophilu	WP_060758019.1
WP_1260445	P-type conjugative transfer protein VirB9 [Wolbachia endosymbiont o	WP_095866610.1
WP_1260445	P-type conjugative transfer protein VirB9 [Wolbachia endosymbiont o	WP_015589382.1
WP_1260445	MULTISPECIES: P-type conjugative transfer protein VirB9 [Wolbachia]	WP_017531859.1
WP_1260445	P-type conjugative transfer protein VirB9 [Anaplasma phagocytophilu	WP_044142394.1

WP_1260445	P-type conjugative transfer protein VirB9 [Wolbachia pipientis]	WP_077188364.1
WP_1260445	P-type conjugative transfer protein VirB9 [Wolbachia endosymbiont o	WP_038249762.1
WP_1260445	P-type conjugative transfer protein VirB9 [Neorickettsia helminthoeca	WP_051579510.1
WP_1260445	MULTISPECIES: conjugal transfer protein TrbG [Ehrlichia]	WP_045804899.1
WP_1260445	P-type conjugative transfer protein VirB9 [Ehrlichia chaffeensis str. He	AHX03354.1
WP_1260445	VirB9 [Rickettsia bellii OSU 85-389]	ABV78839.1
WP_1260445	P-type conjugative transfer protein VirB9 [Ehrlichia chaffeensis]	WP_006010514.1
WP_1260445	P-type conjugative transfer protein VirB9 [Wolbachia endosymbiont o	WP_041045008.1
WP_1260445	P-type conjugative transfer protein VirB9 [Wolbachia endosymbiont o	WP_127463927.1
WP_1260445	PREDICTED: uncharacterized protein LOC105557738 [Vollenhovia eme	XP_011860446.1
WP_1260445	P-type conjugative transfer protein VirB9 [Candidatus Neoehrlichia lot	WP_045808970.1
WP_1260445	P-type conjugative transfer protein VirB9 [Ehrlichia sp. HF]	WP_044194118.1
WP_1260445	P-type conjugative transfer protein VirB9 [Orientia tsutsugamushi]	WP_011944406.1
WP_1260445	P-type conjugative transfer protein VirB9 [Rickettsia felis str. Pedreira	KJV58021.1
WP_1260445	P-type conjugative transfer protein VirB9 [Orientia tsutsugamushi]	WP_064591492.1

% Identity	Alignment le	Mismatches	Gap opens	q. start	q. end	s. start
100	246	0	0	1	246	1
72.072	222	62	0	13	234	22
69.697	231	67	1	4	234	16
69.697	231	67	1	4	234	17
65.254	236	80	1	2	235	9
66.809	235	65	1	14	235	19
54.348	230	96	2	14	234	24
55.963	218	93	2	18	235	30
52.402	229	107	2	18	245	23
50.446	224	108	3	13	235	18
47.5	240	119	5	3	237	14
49.774	221	106	3	18	234	27
51.628	215	99	3	24	234	30
48.78	246	116	5	1	239	11
48.78	246	116	5	1	239	10
48.927	233	109	5	19	242	28
46.473	241	120	4	2	234	4
46.215	251	127	5	1	246	8
50.228	219	104	4	19	235	38
46.502	243	119	4	2	234	4
46.502	243	119	4	2	234	4
46.091	243	120	4	2	234	4
45.299	234	112	3	18	235	24
46.091	243	120	4	2	234	4
47.009	234	108	3	18	235	19
46.091	243	120	4	2	234	4
46.091	243	120	4	2	234	4
45.679	243	121	4	2	234	4
46.091	243	120	4	2	234	4
46.091	243	120	4	2	234	4
46.091	243	120	4	2	234	4
46.091	243	120	4	2	234	4
46.091	243	120	4	2	234	4
46.091	243	120	4	2	234	4
46.091	243	120	4	2	234	4
46.091	243	120	4	2	234	4
46.091	243	120	4	2	234	4
46.091	243	120	4	2	234	4
46.091	243	120	4	2	234	4
46.667	240	117	4	2	234	10

45.679	243	121	4	2	234	4
45.679	243	121	4	2	234	4
45.679	243	121	4	2	234	4
46.091	243	120	4	2	234	4
46.091	243	120	4	2	234	10
46.281	242	119	4	2	233	4
47.009	234	108	3	18	235	24
45.679	243	121	4	2	234	4
46.154	234	110	3	18	235	24
46.694	242	118	4	2	233	4
45.679	243	121	4	2	234	4
46.281	242	119	4	2	233	4
46.154	234	110	3	18	235	24
47.964	221	109	3	19	234	24
45.679	243	121	4	2	234	4
45.267	243	122	4	2	234	4
46.121	232	117	3	10	234	15
45.726	234	119	3	8	234	11
46.748	246	121	5	1	237	4
45.726	234	111	3	18	235	24
45.69	232	118	3	10	234	15
44.156	231	115	3	18	234	23
44.351	239	119	3	11	235	16
44.156	231	115	3	18	234	23
44.889	225	119	3	14	234	17
45.188	239	115	4	11	234	16
44.889	225	119	3	14	234	27
43.723	231	116	3	18	234	23
44.492	236	111	5	18	235	24
43.277	238	121	3	11	234	16
43.277	238	121	3	11	234	16
43.277	238	121	3	11	234	16
46.93	228	117	3	9	233	30
44.538	238	118	3	11	234	16
44.444	234	114	5	18	235	24
42.857	238	122	3	11	234	16
43.277	238	121	3	11	234	16
43.277	238	121	3	11	234	16
43.277	238	121	3	11	234	16
44.444	234	114	5	18	235	24
44.444	234	114	5	18	235	24
43.277	238	121	3	11	234	16
43.277	238	121	3	11	234	16
43.29	231	117	3	18	234	23
44.068	236	112	5	18	235	24

43.277	238	121	3	11	234	16
43.277	238	121	3	11	234	16
44.589	231	116	3	14	235	20
44.77	239	116	4	19	245	25
46.725	229	110	3	19	235	25
48.571	210	100	3	32	234	3
46.725	229	110	3	19	235	36
42.857	238	122	3	11	234	16
42.857	231	118	3	18	234	23
42.449	245	127	3	4	234	134
46.186	236	99	4	19	233	25
44.534	247	123	4	1	235	9
44.813	241	127	3	2	237	12
49.057	212	100	3	30	234	1
44.398	241	128	3	2	237	12

s. end	evalue	Bit score	% Positives
246	0	506	100
243	2.41E-119	350	86.04
243	1.11E-117	345	83.55
244	1.15E-117	345	83.55
244	2.45E-116	342	83.9
253	1.36E-110	328	77.45
253	1.48E-87	270	75.22
244	4.85E-84	260	76.15
250	2.16E-81	254	73.8
239	1.61E-76	241	70.09
251	2.50E-74	236	70.83
246	5.75E-74	234	69.68
243	6.29E-74	234	71.16
253	9.60E-74	234	67.07
252	1.01E-73	234	67.07
259	1.24E-73	234	67.38
243	1.71E-72	231	67.63
255	1.84E-72	231	67.33
253	4.80E-71	228	71.69
245	1.39E-70	226	66.26
245	3.05E-70	225	66.26
245	4.46E-70	224	66.26
257	5.07E-70	225	66.67
245	6.67E-70	224	66.26
252	6.73E-70	224	66.24
245	6.82E-70	224	66.26
245	7.04E-70	224	66.26
245	7.20E-70	224	66.26
245	7.28E-70	224	66.26
245	7.36E-70	224	66.26
245	7.52E-70	224	66.26
245	7.52E-70	224	66.26
245	7.77E-70	224	66.26
245	7.86E-70	224	66.26
245	8.12E-70	224	66.26
245	8.85E-70	224	66.26
245	9.56E-70	224	66.26
245	1.02E-69	224	66.26
245	1.09E-69	223	66.26
245	1.24E-69	223	66.67

245	1.25E-69	223	66.26
245	1.32E-69	223	66.67
245	1.41E-69	223	66.26
245	1.43E-69	223	66.26
251	1.47E-69	223	66.26
244	1.58E-69	223	66.12
257	2.13E-69	223	66.67
245	2.52E-69	223	66.26
257	2.99E-69	223	66.24
244	3.31E-69	222	66.12
245	4.44E-69	222	65.84
244	5.95E-69	222	66.12
257	6.12E-69	222	66.67
243	1.03E-68	221	68.33
245	1.04E-68	221	65.84
245	1.30E-68	221	65.84
245	1.30E-68	221	68.1
243	1.33E-68	221	67.95
248	2.35E-68	220	66.26
257	2.55E-68	221	66.67
245	1.61E-67	218	67.67
253	3.79E-67	218	69.26
254	3.91E-67	218	66.95
253	5.03E-67	217	68.83
240	1.16E-66	216	68
253	1.23E-66	216	66.95
250	1.58E-66	216	68
253	2.28E-66	215	68.83
257	5.39E-66	215	65.68
253	6.40E-66	214	66.39
253	7.70E-66	214	66.39
253	8.58E-66	214	65.55
256	9.15E-66	216	66.23
253	1.07E-65	214	65.13
257	1.29E-65	214	66.67
253	1.46E-65	213	67.23
253	1.60E-65	213	65.97
253	1.67E-65	213	65.97
253	1.72E-65	213	65.97
257	1.73E-65	214	66.67
257	1.80E-65	213	66.67
253	1.82E-65	213	65.97
253	2.52E-65	213	65.97
253	2.75E-65	213	68.4
257	3.07E-65	213	65.25

253	3.68E-65	212	65.97
253	3.77E-65	212	65.55
247	4.87E-65	212	66.67
259	5.18E-65	212	66.95
253	5.84E-65	212	67.25
211	7.07E-65	210	69.05
264	8.46E-65	212	67.25
253	1.10E-64	211	65.97
253	1.40E-64	211	68.4
378	1.64E-64	215	65.31
253	1.73E-64	211	63.98
253	3.32E-64	210	65.59
251	3.54E-64	209	63.9
211	3.62E-64	208	66.98
251	4.04E-64	209	63.9

Supplementary Table 12. Homology to *rvh* genes from related species. While "*Ca.* A. rohweri" has annotations for all necessary components of the *rvh* T4SS, homology to other Rickettsiales varied between genes.

VirB3

Query acc.ve	Description	Subject acc.ver
WP_126044€	type IV secretion system protein VirB3 [Candidatus Marinoinvertebrat	WP_126044686.1
WP_126044€	type IV secretion system protein VirB3 [Candidatus Midichloria mitocl	WP_041185442.1
WP_126044€	type IV secretion system protein VirB3 [Candidatus Jidaibacter acanth	WP_038540306.1
WP_126044€	type IV secretion system protein VirB3 [Rickettsiales endosymbiont of	WP_130122452.1
WP_126044€	type IV secretion system protein VirB3 [Wolbachia pipientis]	WP_038199158.1
WP_126044€	VirB3 [Wolbachia endosymbiont of Segestria florentina]	ACO52123.1
WP_126044€	VirB3 [Wolbachia endosymbiont of Drosophila yakuba]	ACO52101.1
WP_126044€	MULTISPECIES: type VI secretion protein [Wolbachia]	WP_010962887.1
WP_126044€	type IV secretion system protein VirB3 [Wolbachia pipientis]	WP_070064713.1
WP_126044€	type IV secretion system protein VirB3 [Candidatus Fokinia solitaria]	WP_108672977.1
WP_126044€	type IV secretion system protein VirB3 [Wolbachia endosymbiont of C	WP_114517208.1
WP_126044€	MULTISPECIES: type VI secretion protein [Wolbachia]	WP_017532092.1
WP_126044€	VirB3 [Wolbachia endosymbiont of Armadillidium vulgare]	AAX86700.2
WP_126044€	MULTISPECIES: type VI secretion protein [Wolbachia]	WP_007548615.1
WP_126044€	type IV secretion system protein VirB3 [Rickettsiales endosymbiont of	WP_125216219.1
WP_126044€	type IV secretion system protein VirB3 [Wolbachia endosymbiont of B	WP_108784517.1
WP_126044€	MULTISPECIES: type VI secretion protein [Wolbachia]	WP_006015054.1
WP_126044€	MULTISPECIES: type VI secretion protein [Wolbachia]	WP_006279216.1
WP_126044€	type IV secretion system protein VirB3 [Wolbachia endosymbiont of T	WP_068652414.1
WP_126044€	VirB3 [Wolbachia endosymbiont of Delia radicum]	ACO52099.1
WP_126044€	VirB3 [Wolbachia endosymbiont of Musca domestica]	ACO52122.1
WP_126044€	MULTISPECIES: type VI secretion protein [Wolbachia]	WP_014868750.1
WP_126044€	hypothetical protein [Wolbachia endosymbiont of Drosophila simulans	WP_010082402.1
WP_126044€	type IV secretion system protein VirB3 [Wolbachia endosymbiont of B	WP_127463755.1
WP_126044€	type IV secretion system protein VirB3 [Wolbachia endosymbiont of C	WP_041046206.1
WP_126044€	MULTISPECIES: type VI secretion protein [Wolbachia]	WP_011256995.1
WP_126044€	type IV secretion system protein VirB3 [Wolbachia endosymbiont of F	WP_110409799.1
WP_126044€	type VI secretion protein [Ehrlichia ruminantium]	WP_011155206.1
WP_126044€	Conserved hypothetical protein [Ehrlichia ruminantium str. Gardel]	CAI27993.1
WP_126044€	Type IV secretory pathway, VirB3-like protein [Ehrlichia canis str. Jake	AAZ68570.1
WP_126044€	Type IV secretory pathway, VirB3-like protein [Ehrlichia minasensis]	CEI85307.1
WP_126044€	MULTISPECIES: type IV secretion system protein VirB3 [canis group]	WP_044261987.1
WP_126044€	type IV secretion system protein VirB3 [Candidatus Neoehrlichia lotor	WP_045808800.1
WP_126044€	type IV secretion system protein VirB3 [Rickettsia endosymbiont of Cu	WP_094648608.1
WP_126044€	type IV secretion system protein VirB3 [Alphaproteobacteria bacteriu	OFW80368.1
WP_126044€	type IV secretion system protein VirB3 [Ehrlichia sp. Wisconsin_h]	OUC04657.1
WP_126044€	MULTISPECIES: type VI secretion protein [Ehrlichia]	WP_024072156.1
WP_126044€	type IV secretion system protein VirB3 [Ehrlichia sp. HF]	WP_084475730.1
WP_126044€	type IV secretion system protein VirB3 [Rickettsiales bacterium Ac37k	WP_038603729.1
WP_126044€	type VI secretion protein [Ehrlichia chaffeensis]	WP_006009897.1

WP_1260446	type IV secretion system protein VirB3 [Anaplasma ovis]	WP_075139070.1
WP_1260446	type VI secretion protein [Neorickettsia risticii]	WP_015816677.1
WP_1260446	type VI secretion protein [Neorickettsia sennetsu]	WP_011452237.1
WP_1260446	type IV secretion system protein VirB3 [Candidatus Xenolissoclinum p	ETO91782.1
WP_1260446	type IV secretion system protein VirB3 [Neorickettsia sp. 179522]	WP_067980147.1
WP_1260446	type VI secretion protein [Anaplasma centrale]	WP_012880607.1
WP_1260446	MULTISPECIES: type VI secretion protein [Anaplasma]	WP_010265020.1
WP_1260446	VirB3 [Anaplasma marginale str. Florida]	ACM49450.1
WP_1260446	type IV secretion system protein VirB3 [Neorickettsia helminthoeca]	WP_038560093.1
WP_1260446	type VI secretion protein [Anaplasma phagocytophilum]	WP_011450501.1
WP_1260446	type IV secretion system protein VirB3 [Rickettsia argasii]	WP_045805284.1
WP_1260446	MULTISPECIES: type VI secretion protein [spotted fever group]	WP_014013851.1
WP_1260446	type IV secretion system protein VirB3 [Rickettsia monacensis]	WP_023507283.1
WP_1260446	type VI secretion protein [Rickettsia sibirica]	WP_004996714.1
WP_1260446	MULTISPECIES: type VI secretion protein [Rickettsia]	WP_011477926.1
WP_1260446	hypothetical protein BGO27_01745 [Alphaproteobacteria bacterium 3	OJV14205.1
WP_1260446	type IV secretion system protein VirB3 [Rickettsia fournieri]	WP_045812724.1
WP_1260446	MULTISPECIES: type VI secretion protein [spotted fever group]	WP_010976817.1
WP_1260446	MULTISPECIES: type VI secretion protein [spotted fever group]	WP_012013356.1
WP_1260446	type VI secretion protein [Rickettsia parkeri]	WP_014410350.1
WP_1260446	type VI secretion protein [Rickettsia amblyommatis]	WP_014391790.1
WP_1260446	type IV secretion system protein VirB3 [Rickettsia bellii]	WP_045799866.1
WP_1260446	type VI secretion protein [Rickettsia montanensis]	WP_014409875.1
WP_1260446	MULTISPECIES: type IV secretion system protein VirB3 [Rickettsia]	WP_032139311.1
WP_1260446	MULTISPECIES: type VI secretion protein [spotted fever group]	WP_008579695.1
WP_1260446	type VI secretion protein [Rickettsia helvetica]	WP_010423973.1
WP_1260446	type VI secretion protein [Rickettsia typhi]	WP_011190509.1
WP_1260446	type VI secretion protein [Rickettsia prowazekii]	WP_004596465.1
WP_1260446	MULTISPECIES: type VI secretion protein [spotted fever group]	WP_012152425.1
WP_1260446	type IV secretion system protein VirB3 [Rickettsiaceae bacterium]	RYE05937.1
WP_1260446	type IV secretion system protein VirB3 [Alphaproteobacteria bacteriu	PIR37404.1
WP_1260446	type IV secretion system protein VirB3 [Rickettsia aeschlimannii]	WP_032073825.1
WP_1260446	type VI secretion protein [Rickettsia prowazekii]	WP_004599742.1
WP_1260446	type VI secretion protein [Rickettsia canadensis]	WP_012148250.1
WP_1260446	type VI secretion protein [Rickettsia canadensis]	WP_014363557.1
WP_1260446	type IV secretion system protein VirB3 [Orientia chuto]	WP_045796972.1
WP_1260446	type IV secretion system protein VirB3 [Rickettsiales bacterium]	RTK92048.1
WP_1260446	type VI secretion protein [Orientia tsutsugamushi]	WP_012461380.1
WP_1260446	type IV secretion system protein VirB3 [Orientia tsutsugamushi]	WP_109234622.1
WP_1260446	type VI secretion protein [Orientia tsutsugamushi]	WP_011945112.1
WP_1260446	type IV secretion system protein VirB3 [Rickettsia endosymbiont of Pr	WP_062810990.1
WP_1260446	hypothetical protein DI582_04005 [Azospirillum brasilense]	PZP86070.1
WP_1260446	type VI secretion protein [Occidentia massiliensis]	WP_019230791.1
WP_1260446	type VI secretion protein [Sandarakinorhabdus limnophila]	WP_022678818.1
WP_1260446	type VI secretion protein [Gammaproteobacteria bacterium]	RLA54433.1

WP_1260446	type VI secretion protein ['Sphingomonas ginsengisoli' Hoang et al. 20	WP_114228241.1
WP_1260446	type VI secretion protein [Altererythrobacter sp. HME9302]	WP_115365364.1
WP_1260446	type VI secretion protein [Erythrobacter sp. HKB08]	WP_128891767.1
WP_1260446	type VI secretion protein [Altererythrobacter sp. AY-3R]	WP_129525293.1
WP_1260446	type VI secretion protein [Sphingobium yanoikuyae]	WP_037521645.1
WP_1260446	type IV secretion system protein VirB3 [Alphaproteobacteria bacteriu	PIR32614.1
WP_1260446	type IV secretion system protein VirB3 [Candidatus Phycorickettsia tra	WP_106874399.1
WP_1260446	type VI secretion protein [Porphyrobacter sp. LM 6]	WP_069310763.1
WP_1260446	type VI secretion protein [Erythrobacter sp. YH-07]	WP_115416534.1
WP_1260446	MULTISPECIES: type VI secretion protein [Altererythrobacter]	WP_119514376.1
WP_1260446	type VI secretion protein [Phenylobacterium zucineum]	PZQ56382.1
WP_1260446	type VI secretion protein [Sphingosinicella sp. BN140058]	WP_129383959.1
WP_1260446	type VI secretion protein [Novosphingobium sp. B-7]	WP_022678388.1
WP_1260446	type VI secretion protein [Novosphingobium sp. AAP1]	WP_082350267.1
WP_1260446	type VI secretion protein [Alphaproteobacteria bacterium]	RYY92360.1

% Identity	Alignment le	Mismatches	Gap opens	q. start	q. end	s. start
100	96	0	0	1	96	1
72.34	94	26	0	1	94	1
67.708	96	31	0	1	96	1
61.458	96	37	0	1	96	1
59.14	93	37	1	3	94	4
59.14	93	37	1	3	94	4
59.14	93	37	1	3	94	4
59.14	93	37	1	3	94	4
59.14	93	37	1	3	94	4
55.789	95	42	0	1	95	1
59.14	93	37	1	3	94	4
56.989	93	40	0	2	94	3
58.065	93	38	1	3	94	4
58.065	93	38	1	3	94	4
56.383	94	41	0	3	96	4
58.065	93	38	1	3	94	4
58.065	93	38	1	3	94	4
56.989	93	39	1	3	94	4
58.065	93	38	1	3	94	4
56.989	93	39	1	3	94	4
56.989	93	39	1	3	94	4
56.989	93	40	0	2	94	3
56.989	93	39	1	3	94	4
55.914	93	41	0	2	94	3
56.989	93	39	1	3	94	4
56.989	93	39	1	3	94	4
56.989	93	39	1	3	94	4
53.684	95	43	1	1	94	1
53.684	95	43	1	1	94	3
53.684	95	43	1	1	94	5
53.684	95	43	1	1	94	5
53.684	95	43	1	1	94	1
51.579	95	45	1	1	94	1
54.839	93	42	0	1	93	1
52.083	96	44	2	1	94	1
52.632	95	44	1	1	94	2
52.632	95	44	1	1	94	1
51.579	95	45	1	1	94	1
59.14	93	36	2	3	94	5
51.579	95	45	1	1	94	1

50	94	46	1	2	94	3
54.737	95	42	1	3	96	4
54.737	95	42	1	3	96	4
52.688	93	43	1	3	94	4
54.839	93	41	1	3	94	4
47.872	94	48	1	2	94	3
46.809	94	49	1	2	94	3
47.312	93	48	1	3	94	11
52.632	95	44	1	3	96	4
46.316	95	50	1	1	94	1
47.872	94	49	0	1	94	1
47.872	94	49	0	1	94	1
47.872	94	49	0	1	94	1
47.872	94	49	0	1	94	1
50.538	93	46	0	1	93	1
48.421	95	48	1	3	96	4
47.872	94	49	0	1	94	1
47.872	94	49	0	1	94	1
47.872	94	49	0	1	94	1
47.872	94	49	0	1	94	1
47.872	94	49	0	1	94	1
50.538	93	46	0	1	93	1
47.872	94	49	0	1	94	1
46.809	94	50	0	1	94	1
46.809	94	50	0	1	94	1
47.872	94	49	0	1	94	1
47.872	94	49	0	1	94	1
46.809	94	50	0	1	94	1
46.809	94	50	0	1	94	1
48.936	94	48	0	1	94	1
48.387	93	47	1	3	94	4
46.809	94	50	0	1	94	1
46.809	94	50	0	1	94	1
48.387	93	48	0	1	93	1
48.387	93	48	0	1	93	1
42.553	94	54	0	1	94	1
44.681	94	52	0	1	94	1
40.86	93	55	0	1	93	1
40.86	93	55	0	1	93	1
40.86	93	55	0	1	93	1
48.235	85	44	0	10	94	1
46.316	95	50	1	3	96	4
42.553	94	54	0	1	94	1
41.758	91	53	0	5	95	7
34.783	92	60	0	4	95	5

40.217	92	55	0	4	95	3
38.947	95	58	0	1	95	1
40	95	57	0	1	95	1
38.462	91	56	0	5	95	1
38.71	93	57	0	1	93	1
46.939	98	47	3	2	94	4
42.222	90	52	0	4	93	3
38.947	95	58	0	1	95	1
36.842	95	60	0	1	95	1
46.914	81	43	0	13	93	12
40.659	91	54	0	5	95	4
37.634	93	58	0	3	95	2
35.789	95	61	0	1	95	1
36.842	95	60	0	1	95	1
39.785	93	56	0	3	95	2

s. end	evalue	Bit score	% Positives
96	4.23E-64	197	100
94	6.32E-44	147	81.91
96	9.29E-43	144	79.17
96	4.98E-40	137	79.17
96	4.39E-33	119	76.34
96	4.89E-33	119	76.34
96	6.65E-33	119	76.34
96	9.98E-33	118	76.34
96	1.23E-32	118	76.34
95	1.65E-32	118	71.58
96	2.29E-32	117	75.27
95	2.46E-32	117	74.19
96	6.08E-32	116	75.27
96	6.29E-32	116	75.27
97	6.81E-32	116	72.34
96	8.45E-32	116	75.27
96	1.37E-31	115	75.27
96	1.78E-31	115	75.27
96	1.97E-31	115	75.27
96	2.19E-31	115	75.27
96	2.24E-31	115	75.27
95	2.57E-31	115	72.04
96	3.29E-31	114	74.19
95	5.40E-31	114	73.12
96	6.42E-31	114	74.19
96	7.56E-31	114	75.27
96	9.73E-31	113	73.12
95	7.98E-29	108	73.68
97	8.01E-29	108	73.68
99	1.17E-28	108	73.68
99	1.17E-28	108	73.68
95	1.31E-28	108	73.68
95	1.34E-28	108	72.63
93	2.46E-28	107	72.04
96	4.20E-28	107	73.96
96	4.74E-28	107	73.68
95	5.31E-28	106	73.68
95	5.73E-28	106	73.68
96	9.66E-28	106	70.97
95	1.35E-27	105	72.63

96	3.40E-27	104	73.4
98	4.10E-27	104	67.37
98	5.16E-27	104	67.37
96	8.63E-27	103	70.97
96	1.09E-26	103	67.74
96	2.47E-26	102	73.4
96	3.10E-26	102	73.4
103	5.44E-26	102	74.19
98	6.39E-26	101	66.32
95	3.43E-25	99.8	71.58
94	5.54E-25	99	69.15
94	6.74E-25	99	69.15
94	1.00E-24	98.6	69.15
94	1.01E-24	98.2	69.15
93	1.01E-24	98.2	68.82
98	1.05E-24	98.6	70.53
94	1.23E-24	98.2	69.15
94	1.39E-24	98.2	69.15
94	1.43E-24	97.8	69.15
94	1.43E-24	97.8	69.15
94	1.57E-24	97.8	69.15
93	1.67E-24	97.8	68.82
94	1.91E-24	97.8	69.15
94	2.20E-24	97.4	69.15
94	2.40E-24	97.4	69.15
94	2.53E-24	97.4	68.09
94	2.83E-24	97.4	69.15
94	9.53E-24	95.9	69.15
94	9.53E-24	95.9	69.15
94	1.09E-23	95.9	70.21
96	1.49E-23	95.5	68.82
94	1.84E-23	95.1	69.15
94	3.28E-23	94.7	68.09
93	8.40E-23	93.6	66.67
93	1.67E-22	92.8	66.67
94	2.11E-22	92.4	69.15
94	5.95E-22	91.3	65.96
93	6.86E-22	91.3	70.97
93	1.02E-21	90.9	70.97
93	2.05E-21	90.1	69.89
85	7.31E-21	88.2	69.41
98	8.82E-21	88.6	64.21
94	1.29E-20	87.8	67.02
97	6.86E-19	83.6	61.54
96	1.31E-18	82.8	61.96

94	1.42E-18	82.8	60.87
95	2.46E-18	82	56.84
95	3.24E-18	82	57.89
91	5.24E-18	81.3	62.64
93	7.42E-18	80.9	60.22
101	1.36E-17	80.5	62.24
92	1.73E-17	80.1	64.44
95	1.98E-17	79.7	55.79
95	2.01E-17	79.7	56.84
92	2.96E-17	79.3	61.73
94	3.12E-17	79.3	59.34
94	3.23E-17	79.3	56.99
95	3.28E-17	79.3	58.95
95	3.35E-17	79.3	57.89
94	3.53E-17	79.3	60.22

Supplementary Table S13. Summary of sample categories that had the greatest number of samples with OTUs identified as "*Ca.* Midichloriaceae." Rows in white were phylogenetically assigned to "*Ca* . Marinoinvertebrata," rows in grey fell outside of the genus.

GG OTU ID	Closest NCBI	Top Sample Categories	Host full name
150441	"Ca. Aquarickettsia rohweri," *Acropora cervicornis*	Sponge tissue 961, freshwater 781, seawater 496, coral 457, marine sediment 118, beach sand 71, kelp mucus 61	"Ca. A. rohweri"
256955	EF667901, Hydra	freshwater 1720, sponge tissue 631, seawater 455, water 243, seawater mesocosm 166, filtration sand 163, marine sediment 121	Uncultured Rickettsiales bacterium clone Ho(lakePloen)_13
365956	FJ425643, Orbicella faveolata	sponge tissue 198, seawater 49, coral 44, fragment 28	Uncultured alpha proteobacterium clone MD3.55
347118	EU394580, particle-associated in seawater	sponge tissue 169, seawater 104	Uncultured proteobacterium clone PEACE2006/237 _P3
590270	GU118640, Orbicella faveolata	coral 48, sponge tissue 28, seawater 10	Uncultured bacterium clone Mfav_F04
589550	GU119441, reef water	sponge tissue 3, marine sediment 2, swab 2, filter 1	Uncultured bacterium clone Reef_O20
663562	HM128969, Kelike Lake (Tibet)	fresh water 1616, water 301, filtration sand 76	Uncultured alpha proteobacterium clone SING1046
585787	GQ302530, cold spring sediment	soil 624, unknown source 241, freshwater 142, filtration sand 72, peat 62	Uncultured alpha proteobacterium clone sw-xj63
1132275	AB624350, Nysius plebeius	dust 342	Candidatus Lariskella arthropodarum, Rickettsiales bacterium endosymbiont of Nysius plebeius
5951	AF069962 Jidaibacter acanthamoeba, Acanthamoeba sp. UWC36	soil 137, freshwater 130	Candidatus Jidaibacter acanthamoeba
560977	EU555284, diseased rainbow trout	filtration sand 115, water 89, freshwater 44, filtered water 15	Uncultured Rickettsiales bacterium clone ID25L
4476653	FM992372, Ixodes holocyclus	freshwater 199, water 35	Candidatus Midichloria sp. Ixholo1

4236006	JN515431, microbial mat in hypersaline pond	photosynthetic mat 165	Uncultured organism clone SBZF_8564
810074	GQ480088, activated sludge from wastewater treatment plant	water 75, freshwater 45	Uncultured bacterium clone BXHB42
736450	FN552698, Euplotes harpa strain BOD18	water 9, freshwater 8, coral 3, sponge tissue 2, fragment 1	Candidatus Cyrtobacter comes partial 16S rRNA gene, clone 121
768789	FN552695, Euplotes harpa strain HS11/7	fresh water 9, freshwater sediment 3, water 2, wetland soil 2, peat 1, biofilm 1	Candidatus Anadelfobacter veles, host strain HS11/7

Supplementary Table S14. SRA database was queried with the full-length 16S rRNA sequence of "Ca. A. rohweri" using IMNGS at 97% and 99% similarity thresholds.

IMNGS Sample ID	No. Seqs. in sample	Sample Description	99%	97%	Rel. Abund. In Sample
SRR6401683	88954	marine metagenome	2033	2033	2.285450907
SRR6401547	86021	marine metagenome	1983	1983	2.305251043
SRR3145938	10887	coral metagenome	828	830	7.605400937
SRR6401721	51707	marine metagenome	329	329	0.636277487
SRR6401360	19612	marine metagenome	147	147	0.749541097
SRR6401804	39905	marine metagenome	39	39	0.097732114
SRR6401684	73878	marine metagenome	33	33	0.044668237
SRR6401616	86255	marine metagenome	10	10	0.011593531
SRR6401692	25716	marine metagenome	8	8	0.031109037
SRR6401654	43923	marine metagenome	4	4	0.009106846
SRR3145887	5672	coral metagenome	3	3	0.052891396
SRR3145895	3092	coral metagenome	2	2	0.064683053
SRR3145920	3745	coral metagenome	2	2	0.053404539
SRR6401365	57287	marine metagenome	2	2	0.003491193
SRR6401462	42464	marine metagenome	2	2	0.004709872
SRR2984259	8874	marine metagenome	1	1	0.011268875
SRR3145838	8896	coral metagenome	1	1	0.011241007
SRR3145841	7110	coral metagenome	1	1	0.014064698
SRR3145894	6676	coral metagenome	1	1	0.014979029
SRR6401540	2157	marine metagenome	1	1	0.046360686
SRR6401807	35151	marine metagenome	1	1	0.002844869
DRR000776	380581	human gut metagenome	0	0	0
