# Supplementary figures and images for: Phylogenetic, genomic, and biogeographic characterization of a novel and ubiquitous marine invertebrate-associated Rickettsiales parasite, Candidatus Aquarickettsia rohweri, gen. nov., sp. nov
Source: ISME J. 2019 Aug 5;13(12):2938–53. doi: 10.1038/s41396-019-0482-0 (PMC6863919; doi:10.1038/s41396-019-0482-0)

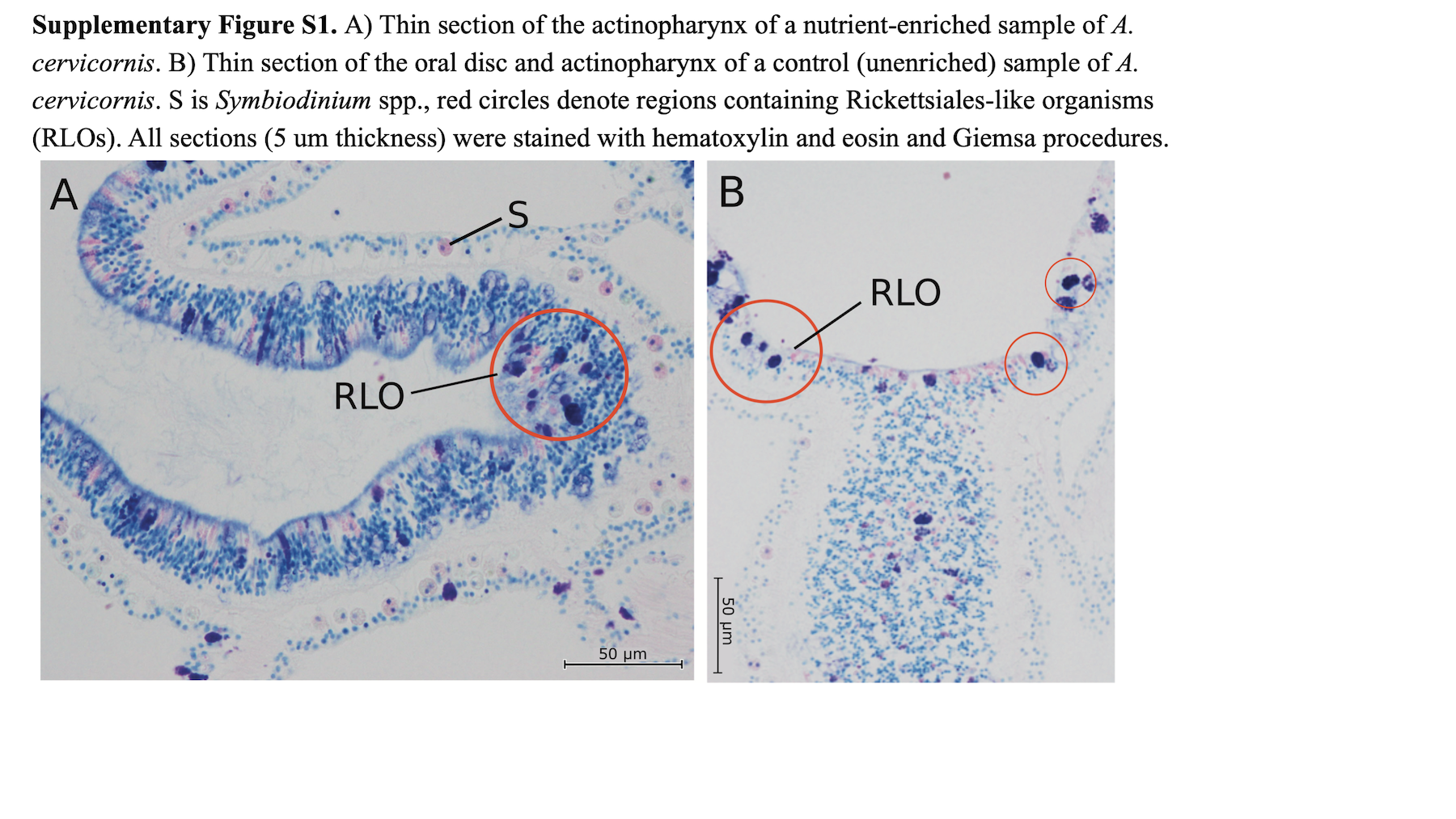

Supplement: Supplementary file 2 — Supplementary Figure S1 [file 41396_2019_482_MOESM2_ESM.tif]

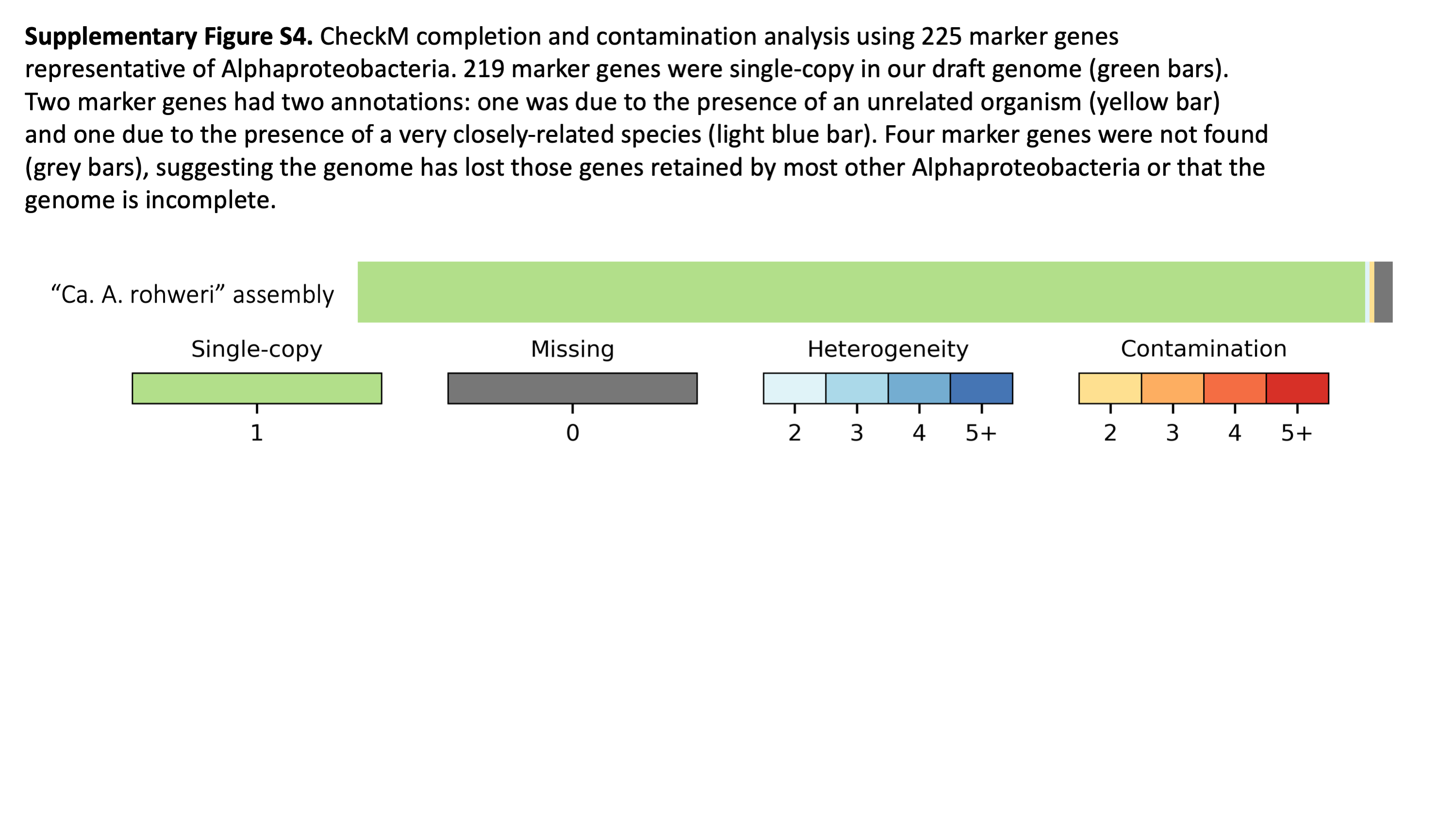

Supplement: Supplementary file 5 — Supplementary Figure S4 [file 41396_2019_482_MOESM5_ESM.tif]

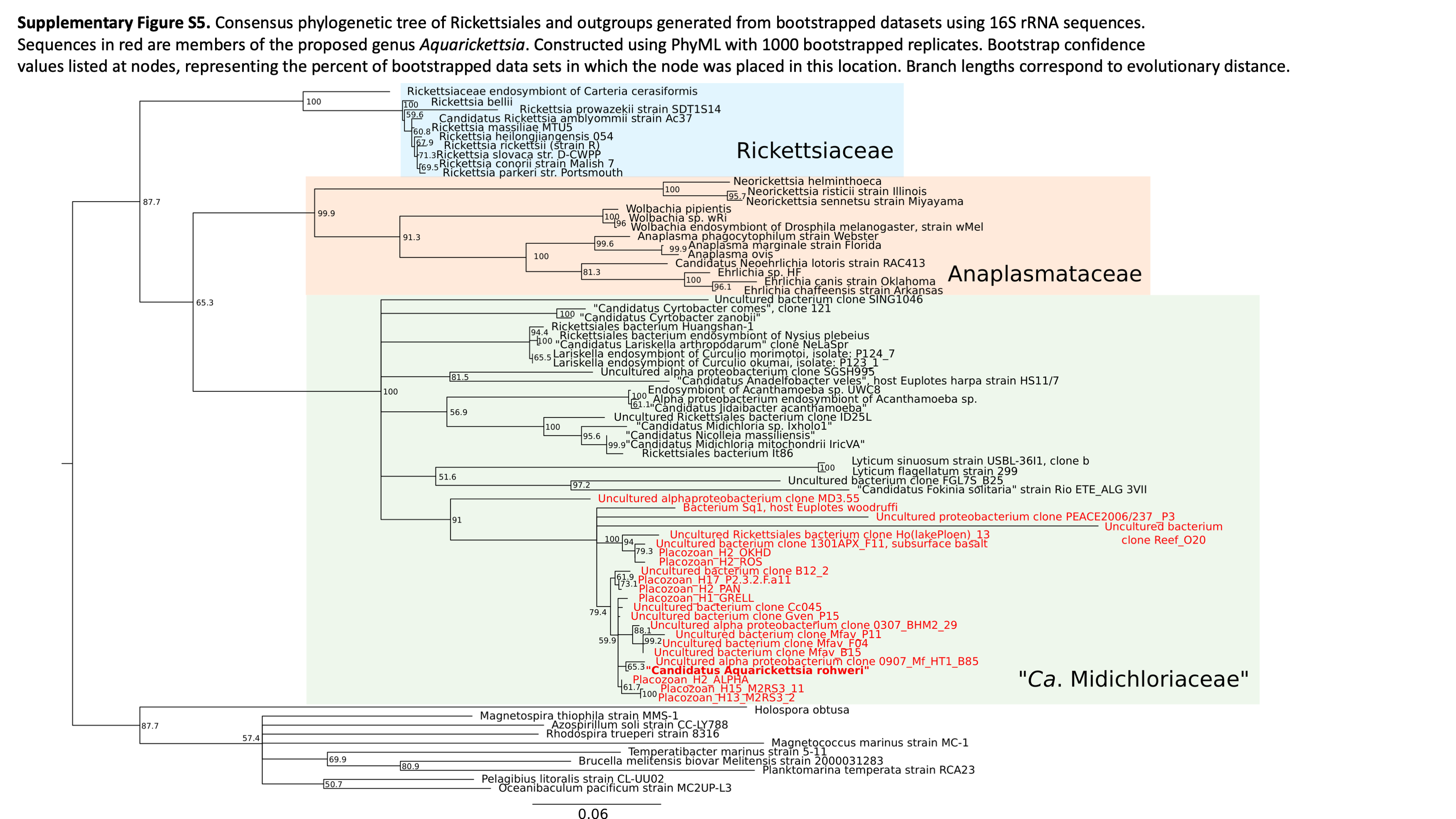

Supplement: Supplementary file 6 — Supplementary Figure S5 [file 41396_2019_482_MOESM6_ESM.tif]
